# Systematic Review of Cerebral Phenotypes Associated With Monogenic Cerebral Small‐Vessel Disease

**DOI:** 10.1161/JAHA.121.025629

**Published:** 2022-06-14

**Authors:** Ed Whittaker, Sophie Thrippleton, Liza Y. W. Chong, Victoria G. Collins, Amy C. Ferguson, David E. Henshall, Emily Lancastle, Tim Wilkinson, Blair Wilson, Kirsty Wilson, Cathie Sudlow, Joanna Wardlaw, Kristiina Rannikmäe

**Affiliations:** ^1^ Medical School University of Edinburgh Edinburgh United Kingdom; ^2^ Centre for Medical Informatics Usher Institute University of Edinburgh Edinburgh United Kingdom; ^3^ Centre for Clinical Brain Sciences University of Edinburgh Edinburgh United Kingdom; ^4^ NHS Greater Glasgow and Clyde Glasgow United Kingdom; ^5^ NHS Lothian Edinburgh United Kingdom; ^6^ BHF Data Science Centre London United Kingdom; ^7^ UK Dementia Research Institute Centre University of Edinburgh Edinburgh United Kingdom

**Keywords:** Mendelian, radiological features, small‐vessel disease, stroke, systematic review, Genetics, Cerebrovascular Disease/Stroke

## Abstract

**Background:**

Cerebral small‐vessel disease (cSVD) is an important cause of stroke and vascular dementia. Most cases are multifactorial, but an emerging minority have a monogenic cause. While *NOTCH3* is the best‐known gene, several others have been reported. We aimed to summarize the cerebral phenotypes associated with these more recent cSVD genes.

**Methods and Results:**

We performed a systematic review (PROSPERO [International Prospective Register of Systematic Reviews]: CRD42020196720), searching Medline/Embase (conception to July 2020) for any language publications describing *COL4A1/2*, *TREX1*, *HTRA1*, *ADA2*, or *CTSA* pathogenic variant carriers. We extracted data about individuals’ characteristics and clinical and vascular radiological cerebral phenotypes. We summarized phenotype frequencies per gene, comparing patterns across genes. We screened 6485 publications including 402, and extracted data on 390 individuals with *COL4A1*, 123 with *TREX1*, 44 with *HTRA1* homozygous, 41 with *COL4A2*, 346 with *ADA2*, 82 with *HTRA1* heterozygous, and 14 with *CTSA*. Mean age ranged from 15 (*ADA2*) to 59 years (*HTRA1* heterozygotes). Clinical phenotype frequencies varied widely: stroke, 9% (*TREX1*) to 52% (*HTRA1* heterozygotes); cognitive features, 0% (*ADA2*) to 64% (*HTRA1* homozygotes); and psychiatric features, 0% (*COL4A2*; *ADA2*) to 57% (*CTSA*). Among individuals with neuroimaging, vascular radiological phenotypes appeared common, ranging from 62% (*ADA2*) to 100% (*HTRA1* homozygotes; *CTSA*). White matter lesions were the most common pathology, except in *ADA2* and *COL4A2* cases, where ischemic and hemorrhagic lesions dominated, respectively.

**Conclusions:**

There appear to be differences in cerebral manifestations across cSVD genes. Vascular radiological changes were more common than clinical neurological phenotypes, and present in the majority of individuals with reported neuroimaging. However, these results may be affected by age and biases inherent to case reports. In the future, better characterization of associated phenotypes, as well as insights from population‐based studies, should improve our understanding of monogenic cSVD to inform genetic testing, guide clinical management, and help unravel underlying disease mechanisms.

**Nonstandard Abbreviations and Acronyms**
cSVD: cerebral small‐vessel disease
HetZ: heterozygous
HomZ: homozygous or compound heterozygous
ICH: intracerebral hemorrhage
OMIM: Online Mendelian Inheritance in Man
PROSPERO: International Prospective Register of Systematic Reviews
PVSs: perivascular spaces
VEP: Variant Effect Predictor
WMLs: white matter lesions

**Clinical Perspective**

**What Is New?**

- We present a large systematic review allowing comparisons to be made across the cerebral manifestations of several cerebral small‐vessel disease genes, following a comprehensive search strategy including abstracts and foreign‐language papers.
- Neuroimaging appears particularly important in detecting early or otherwise clinically asymptomatic disease (radiological vascular phenotypes were more common than clinical neurological phenotypes).
- Cognitive involvement appeared even more frequently than clinical stroke for several genes.

**What Are the Clinical Implications?**

- The findings summarized here have clinical implications for the diagnosis of these rare genetic diseases, especially in conjunction with similar summaries of their extracerebral phenotypes published elsewhere, potentially allowing more informed clinical management of symptoms and disease progression.
- There may be a role for radiological screening for earlier diagnosis in patients and at‐risk family members, but more research is needed to explore this further.
- The frequency profile of clinical cerebral phenotypes associated with monogenic cerebral small‐vessel diseases suggests that it is important to consider a broad spectrum of manifestations when identifying potential patients for genetic testing.

Cerebral small‐vessel disease (cSVD) is recognized as an important cause of stroke and vascular cognitive impairment worldwide. The term *cSVD* describes a group of pathological processes that affect the small arteries, arterioles, venules, and capillaries within the brain.^1^ Features of cSVD on neuroimaging include subcortical infarcts, white matter lesions (WMLs), deep intracerebral hemorrhage (ICH), enlarged perivascular spaces (PVSs), cerebral microbleeds, and brain atrophy.^2^ Despite the increase in cSVD burden among an aging population, the underlying disease mechanisms are incompletely understood, and therapeutic options limited, with vascular risk factor management remaining the mainstay of cSVD prevention and treatment.^3^

While the majority of cSVD cases are thought to result from the interaction of multiple genetic variants and environmental factors, an important minority of cases are monogenic, that is, caused by a pathogenic rare variant in a single gene. *NOTCH3* (Notch Receptor 3) is the best known of these genes and is implicated in cerebral autosomal dominant arteriopathy with subcortical infarcts and leukoencephalopathy.^4^ However, since NOTCH3 was first described in 1996, several additional cSVD genes have been identified, including *COL4A1* (Collagen, Type Iv, Alpha‐1), *TREX1* (3‐Prime Repair Exonuclease 1), *HTRA1* (HTRA Serine Peptidase 1), *COL4A2* (Collagen, Type Iv, Alpha‐2), *ADA2* (Adenosine Deaminase 2) and, most recently, *CTSA* (Cathepsin A). Pathogenic rare variants in these genes have been associated with various clinical phenotypes alongside cSVD, including extracerebral manifestations (Table 1), as well as certain radiological features seen on neuroimaging.^5^

**Table 1:** Modes of Inheritance and Extracerebral Features for Each Gene

Gene	Mode of inheritance	Extracerebral features
*COL4A1/COL4A2*	AD	Retinal artery tortuosity*; cataract; kidney cysts; hematuria; muscle cramps and raised creatinine kinase; anterior segment defects; arrhythmia; Raynaud phenomenon; hemolytic anemia
*TREX1*	AD	Retinal vasculopathy; nephropathy; liver disease; Raynaud phenomenon; skin lesions
*HTRA1*	AR/AD	Hair loss; degenerative spine disease; back pain
*ADA2*	AR	Inflammation; skin involvement; liver disease; nephropathy; splenomegaly; myalgia; hematological features
*CTSA*	AR	Hypertension; dry mouth/eyes; muscle cramps

AD indicates autosomal dominant; and AR, autosomal recessive.

* The relationship between this phenotype and the gene is classed as provisional in the Online Mendelian Inheritance in Man (OMIM) database. Otherwise, all phenotype‐genotype relationships are classed as established in OMIM or were taken from the first reporting where not included in the OMIM database (*CTSA*).

Better characterization of these rare disorders, including which radiological and clinical phenotypes are associated with specific genes, can inform genetic testing and counseling, including the appropriate selection of patients and screening of family members. This knowledge can also aid in the management of affected individuals, for example, by guiding appropriate screening for certain associated phenotypes. Furthermore, an improved understanding of monogenic cSVD may offer insights into the disease mechanisms underlying sporadic cSVD, as there is increasing evidence to suggest an overlap of disease pathways involved in both sporadic and monogenic disease.^6^, ^7^, ^8^ Observations from large‐scale genetic association studies have also shown common variation in monogenic cSVD genes to be associated with sporadic cSVD. Examples include *COL4A2* single‐nucleotide polymorphisms’ association with lacunar ischemic stroke and deep ICH, *HTRA1* single‐nucleotide polymorphism association with ischemic stroke, and possibly association of *NOTCH3* single‐nucleotide polymorphisms with WMLs.^9^, ^10^, ^11^, ^12^

We undertook a systematic literature review with the aim of identifying all reported individuals with putative pathogenic rare variants in any of the following monogenic cSVD genes: *COL4A1*, *TREX1*, *HTRA1*, *COL4A2*, *ADA2* and *CTSA*. We aimed to summarize and compare both clinical and vascular radiological cerebral phenotypes associated with each monogenic cSVD gene.

## METHODS

As a systematic review based on data from published studies, this work does not require approval from an ethical standards committee.

### Transparency and Openness Promotion Statement

The authors declare that all supporting data are available within the article (and its supplemental material).

### Registration

We have registered a PROSPERO (International Prospective Register of Systematic Reviews) protocol (ID: CRD42020196720) at https://www.crd.york.ac.uk/prospero/display_record.php?ID=CRD42020196720.^13^ We followed the Preferred Reporting Items for Systematic Reviews and Meta‐analyses guidelines.^14^

### Search Strategy

We searched the MEDLINE and EMBASE databases using OvidSP (from conception to July 2020) for publications about individuals with pathogenic rare variants in any of our genes of interest: *COL4A1*, *TREX1*, *HTRA1*, *COL4A2*, *ADA2*, or *CTSA*. We did not restrict the search by language or publication date; we limited it to human studies; and we included conference abstracts. We used a previously published search strategy (Data S1).^5^ In summary, the search included:

1. Text words, phrases, and Medical Subject Headings for relevant monogenic syndromes/diseases associated with our genes of interest, and
2. Text words, phrases, and Medical Subject Headings terms associated with cSVD combined with those for our genes of interest and their proteins.

### Screening

We carried out the screening using Covidence (www.covidence.org). At least two reviewers (E. W., S. T., L. Y. W. C., D. E. H., B. W., K. R.) independently screened titles and abstracts of all publications identified in our search, blinded to each other’s decisions. Full texts of studies included at this stage were then retrieved and screened by 2 reviewers for eligibility, recording any reasons for exclusion. We resolved disagreements through discussion and mutual consensus with a third reviewer. The included publications were combined with those identified via a previous systematic review.^5^

### Inclusion/Exclusion Criteria

We included studies that met the following conditions:

1. A case report, case series, or other study design (except review papers) describing the clinical or cerebral radiological phenotype of ≥1 individual. Such description could be anything between stating that the individual was healthy to an in‐depth case report.
2. Genetically confirmed rare variant (in a heterozygous [HetZ] or homozygous or compound heterozygous state [HomZ]) in any of our genes of interest.
3. Study authors considered the rare variant to be probably or definitely pathogenic.

We excluded studies describing individuals with rare variants in *CTSA* and *TREX1* associated with galactosialidosis, Aicardi‐Goutieres syndrome, and chilblain or systemic lupus. We excluded individuals with a presumed pathogenic variant in >1 gene.

### Data Extraction

From each included publication, we (one of E. W., S. T., L. Y. W. C., V. C., E. L., D. E. H., K. R.) extracted data on the first author, publication year, journal, and number of eligible individuals and pedigrees. For foreign language articles, we sought a full translation where an English language abstract did not provide sufficient information or was not available. For each eligible individual, we extracted data using a standardized form, including:

1. The individual’s characteristics (region of origin, sex, age at time of assessment); genetic variant, and resulting protein change;
2. Clinical cerebral phenotype (presence, type and age at diagnosis of clinical stroke[s], cognitive features, psychiatric features, and headache);
3. Vascular radiological cerebral phenotype (presence, location, burden, scan type used, age at diagnosis of ischemia, ICH, WMLs, microbleeds, atrophy, enlarged PVSs, calcification, and cerebral aneuryms); and
4. Vascular risk factors (presence of ≥1 of hypertension, smoking, diabetes, excess alcohol consumption, or hypercholesterolemia).

We selected the list of clinical cerebral phenotypes to extract to represent known manifestations of cSVD, including stroke, and the broad categories of cognitive and psychiatric features. We additionally included headache as phenotype of interest because of its association with several monogenic cSVD genes in the Online Mendelian Inheritance in Man (OMIM) database (*ADA2*, *COL4A1*, *TREX1*, and *HTRA1*). Finally, we also noted any other cerebral clinical phenotypes on our data extraction form.

We selected the list of vascular radiological cerebral phenotypes to extract to represent known manifestations of cSVD and again noted any other features on our data extraction form. Finally, we noted any specific radiological patterns to lesion location or severity that might help identify cases in everyday clinical practice.

To assess agreement in data extraction, at least 2 members of the team extracted data from 10% of publications, working independently and blinded to each other’s decisions.

Where radiological imaging findings were described, the terminology used across publications varied widely, as has been noted previously in the literature.^2^ We made an effort to sort the imaging descriptions into our prespecified categories to deal with the variable terminology (see Data S1 for a list of decisions and assumptions), discussing uncertainties with an expert neuroradiologist (J.W.).

### Data Synthesis

For each gene, we summarized the total number of relevant publications, pedigrees, individuals and rare variants, and the individuals’ characteristics. We summarized data on the presence or absence of each cerebral phenotype (clinical and vascular radiological) as well as cumulative evidence of any vascular radiological feature, to assess their apparent frequency. We compared findings between genes, highlighting shared patterns and differences in the frequencies of associated phenotypes.

We stratified the presence of clinical stroke and any vascular feature(s) on neuroimaging by presence of ≥1 vascular risk factors. We used the chi‐squared test (significance threshold of 0.05) to assess differences in phenotype frequency in patients with and without vascular risk factors.

### Variant Pathogenicity Assessment

We used the Ensembl Variant Effect Predictor (VEP)^15^ to assess the consequences of the genetic variants included in our systematic review. We extracted information on the variants on the basis of the following VEP subcomponents: (1) SnpEff variant annotation and effect prediction tool to assess variant impact^16^; (2) ClinVar to assess variant’s clinical significance^17^; (3) SIFT to predict whether an amino acid substitution is likely to affect protein function^18^; and (4) Polymorphism Phenotyping v2 to predict the effect of an amino acid substitution on the structure and function of a protein.^19^ Where conflicting evidence was provided for the same variant (usually because an allele may have a different effect in different transcripts), we selected the category with a more significant/negative effect. We calculated the results (expressed as percentages) among variants per each individual VEP subcomponent.

## RESULTS

**Figure 1:**
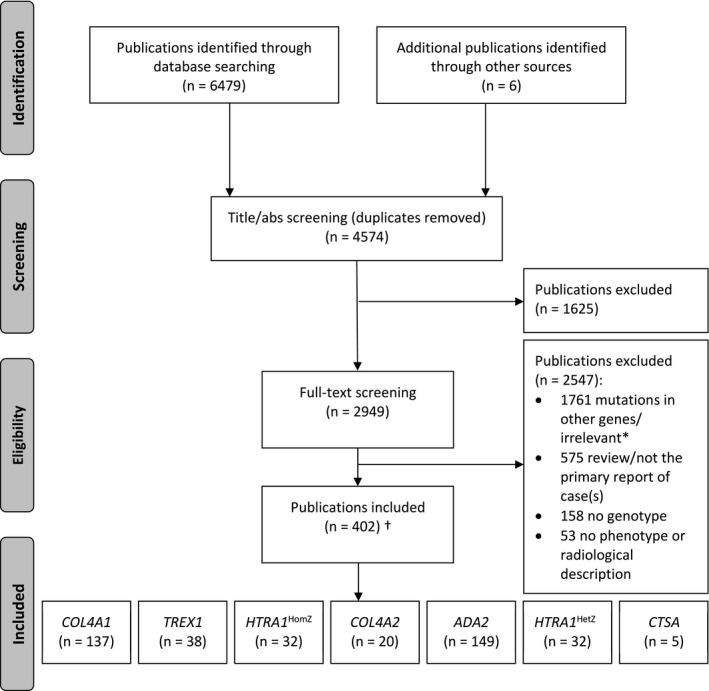
Selection of included publications. abs indicates abstract; HetZ¸ heterozygous; and HomZ, homozygous/compound heterozygous. *We identified *NOTCH3*, *FOXC1* and *PITX2* individuals as part of another systematic review. ^†^One publication reported both individuals with *HTRA1* ^HomZ^ and individuals with *HTRA1* ^HetZ^, 7 publications reported both individuals with *COL4A1/2*, and 1 publication reported individuals with *HTRA1* ^HetZ^, *COL4A1/2*, and *TREX1*, so the number of unique publications (402) is not the sum of publications per gene (413).

**Figure 2:**
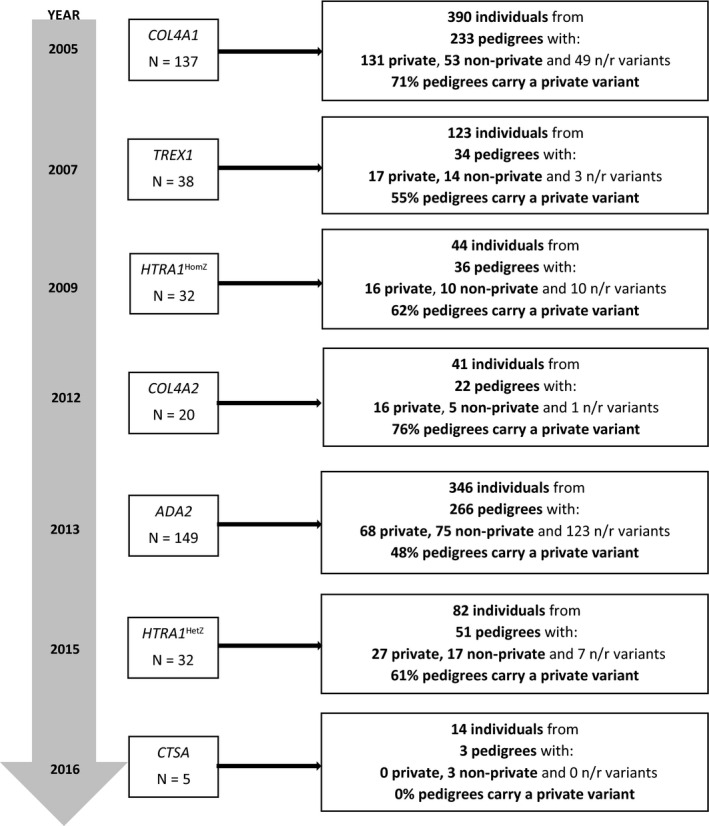
Number of included individuals and pedigrees. This figure is reporting on DNA change, variant was considered n/r where DNA change was not reported. For compound heterozygotes, if either variant was private, the pedigree was considered to carry a private variant. Where publications had not clearly reported these data (eg, reporting 5 individuals with pathogenic *COL4A1* variants, but not specifying the variants, could refer to 5 individuals all carrying the same variant or each carrying a private variant), we assumed the maximum number of private variants (eg, 5 private variants in this example). HetZ indicates heterozygous; HomZ, homozygous/compound heterozygous; n/r, not reported; and year, year gene first reported to be associated with cSVD.

We included 402 publications from 6485 identified for screening (Figure 1, Supplemental References). As in our previous systematic review,^5^ despite only being first reported in 2013, *ADA2* had the largest number of eligible publications (n=149), while the number of publications for other genes appears to be related to their order of discovery (*COL4A1*, n=137; *TREX1*, n=38; *HTRA1* ^HomZ^, n=32; *COL4A2*, n=20; *HTRA1* ^HetZ^, n=32; *CTSA*, n=5) (Figure 2). A likely explanation is the combination of existing treatment options and the severe early‐onset systemic phenotype of *ADA2*, prompting more widespread genetic testing. We extracted data on 1040 individuals, with the number of individuals per gene ranging from 14 (*CTSA*) to 390 (*COL4A1*), and the number of pedigrees ranging from 3 (*CTSA*) to 266 (*ADA2*). The percentage of pedigrees carrying a private variant ranged from 0% (*CTSA*) to 76% (*COL4A2*). As expected, the proportion carrying a private variant has decreased since our previous systematic review,^5^ presumablybecause of new reported individuals now becoming increasingly likely to have had their rare variant identified previously (Figure 2).

The subset of included studies with data independently extracted for comparison showed 96.3% agreement.

### Summary of Individuals’ Characteristics

The most common region of origin was Europe for individuals with *COL4A1*, *TREX1*, *COL4A2*, and *CTSA* (67% [263/390], 57% [70/123], 49% [20/41], and 100% [14/14], respectively); Asia for individuals with *HTRA1* ^HomZ^ and *HTRA1* ^HetZ^ (75% [33/44] and 56% [46/82]); and Turkey for individuals with *ADA2* (28% [98/346]). The region of origin was unknown in 0% to 16% of individuals per gene.

Sex distribution was generally approximated equal (45%–52% female sex) where the number of individuals per gene was considered sufficient to allow meaningful comparison (>100 individuals per gene).

Data about the age of individuals at the time of assessment were not available for >20% of *COL4A1/2* individuals. Mean (median) age ranged from 15 (13) years for individuals with *ADA2* to 59 (60) years for individuals with *HTRA1* ^HetZ^. For *COL4A1/2* and *ADA2*, the median age of individuals was <18 years, while the age ranges were broad (ranging from <1 to 77, 72, and 76, respectively) (Table 2).

**Table 2:** Summary of Case Characteristics

	*COL4A1* (N=390)	*TREX1* (N=123)	*HTRA1* ^HomZ^ (N=44)	*COL4A2* (N=41)	*ADA2* (N=346)	*HTRA1* ^HetZ^ (N=82)	*CTSA* (N=14)
Region of origin*							
European	67 (263/390)	57 (70/123)	11 (5/44)	49 (20/41)	27 (95/346)	40 (33/82)	100 (14/14)
Asian	15 (57/390)	14 (17/123)	75 (33/44)	20 (8/41)	18 (62/346)	56 (46/82)	0 (0/14)
Turkish	6 (25/390)	1 (1/123)	7 (3/44)	0 (0/41)	28 (98/346)	2 (2/82)	0 (0/14)
North American	7 (29/390)	24 (30/123)	2 (1/44)	15 (6/41)	6 (21/346)	0 (0/82)	0 (0/14)
South American	0 (0/390)	0 (0/123)	0 (0/44)	0 (0/41)	2 (6/346)	0 (0/82)	0 (0/14)
African	0 (0/390)	0 (0/123)	0 (0/44)	0 (0/41)	2 (8/346)	1 (1/82)	0 (0/14)
Australian	<1 (1/390)	4 (5/123)	5 (2/44)	10 (4/41)	0 (0/346)	0 (0/82)	0 (0/14)
Unknown	4 (15/390)	0 (0/123)	0 (0/44)	7 (3/41)	16 (56/346)	0 (0/82)	0 (0/14)
Sex							
Female/male	52/48 (160/146)	45/55 (54/65)	55/45 (22/18)	38/62 (15/24)	49/51 (132/140)	34/66 (27/52)	86/14 (12/2)
Sex not reported	22 (84/390)	3 (4/123)	9 (4/44)	5 (2/41)	21 (74/346)	4 (3/82)	…
Age at time of assessment^†^							
Mean, y	22	44	36	23	15	59	57
Median, y	17	…	34	15	13	60	55
Range, y	<1–77	…	24–52	<1–72	<1–76	31–86	39–74
Age *not* reported, %	28	14	11	22	20	10	0

Variables were reported as percentage (proportion). HetZ indicates heterozygous; and HomZ, homozygous/compound heterozygous.

* Region of origin assumed from first author’s institution country: 179/390 individuals with *COL4A1*, 19/123 with *TREX1*, 10/44 with *HTRA1* ^HomZ^, 21/41 with *COL4A2*, 152/346 with *ADA2*, and 25/82 with *HTRA1* ^HetZ^. We could not derive this for 15 individuals with *COL4A1*, 3 with *COL4A2*, and 56 with *ADA2*. Individuals reported to have a different region of origin/ancestry from that of the country they lived in were considered to be from their region of origin (eg, Chinese‐origin person living in the United States was considered Asian).

^†^ If mean age was available for a group of individuals, the overall summary estimate was weighted by group size. For 78/123 individuals with *TREX1*, only mean age was reported; therefore, they were included in the calculations for mean but not for median age/age range. Turkey was reported on specifically because of high proportion of individuals with *ADA2* from there.

### Frequency of Clinical Cerebral Phenotypes

**Figure 3:**
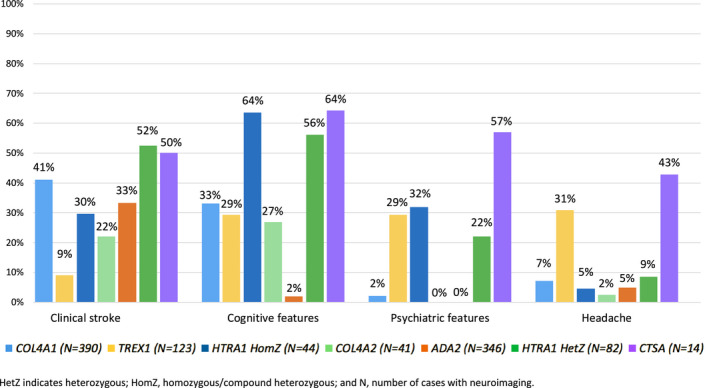
Frequency of clinical cerebral phenotypes by gene. HetZ indicates heterozygous; and HomZ, homozygous/compound heterozygous.

Cognitive features were the most common clinical cerebral phenotype for 4 of 7 genes (*HTRA1* ^HomZ^, *COL4A2*, *HTRA1^H^* ^etZ^, and *CTSA*); stroke was the most common among individuals with *COL4A1* and *ADA2*, and headache was most common among individuals with *TREX1* (Figure 3, Table S1).

#### Stroke

The frequency of clinical stroke ranged from 22% to 52% for 6 of 7 genes (*COL4A2*, 22% [9/41]*; HTRA1* ^HomZ^, 30% [13/44]*; ADA2*, 33% [115/346]; *COL4A1*, 41% [161/390]; *CTSA*, 50% [7/14]; *HTRA1* ^HetZ^, 52% [43/82]), while only 9% (11/123) of *TREX1* individuals were reported to have suffered a clinical stroke. Hemorrhagic events (ICH, porencephaly, and intraventricular hemorrhage) were the most commonly reported stroke type among *COL4A1/2* individuals, affecting 73% (118/161) and 100% (9/9) of stroke cases, respectively. Ischemic events (including arterial and venous ischemic stroke, transient ischemic attacks, and ocular vascular occlusions) were most common for all other genes and were reported in 54% to 100% of stroke cases (*HTRA1* ^HomZ^, 54% [7/13]; *ADA2*, 61% [70/115]; *HTRA1* ^HetZ^, 62% [27/43]; *TREX1*, 82% [9/11]; *CTSA*, 100% [7/7]), although hemorrhagic events also occurred in a substantial minority.

#### Cognitive Features

The frequency of cognitive features ranged from 27% to 64% for 6 of 7 genes (*COL4A2*, 27% [11/41]; *TREX1*, 29% [36/123]; *COL4A1*, 33% [128/390]; *HTRA1* ^HetZ^, 56% [46/82]; *HTRA1* ^HomZ^, 64% [28/44]; and *CTSA*, 64% [9/14]), while only 2% [7/346] of individuals with *ADA2* were reported to have cognitive features. Developmental delay was present in over 80% of individuals with *COL4A1/2* with cognitive features; however, no cases of developmental delay were reported for other genes. For other genes, publications were generally lacking in detail, so we could not draw conclusions about the nature and severity of cognitive decline (ie, cognitive impairment versus dementia).

#### Psychiatric Features

The frequency of psychiatric features ranged from 22% to 57% for 4 of 7 genes (*HTRA1* ^HetZ^, 22% [18/82], *TREX1*, 29% [36/124], *HTRA1* ^HomZ^, 32% [14/44], and *CTSA*, 57% [8/14], in ascending order of frequency). The most commonly reported psychiatric features were depression, followed by irritability or agitation. In contrast, only 2% (8/390) of individuals with *COL4A1* reported psychiatric features, and no psychiatric features were reported among individuals with *COL4A2* and *ADA2* (Table S1).

#### Headache

Headache was reported in 31% (38/123) of *TREX1* individuals and 43% (6/14) of *CTSA* individuals, with >80% of headache cases being specified as migraine. For all other genes, the frequency of headache ranged from 2% to 10%.

#### Other Clinical Cerebral Phenotypes

Thirty‐two percent of individuals with *COL4A1/2* (123/390 and 13/41, respectively) were reported to have suffered a seizure or have epilepsy. Forty‐three percent of individuals with (6/14) *CTSA* were reported to suffer from vertigo or balance problems of unclear etiology but suggested to signify brain stem and lower cranial nerve involvement.

### Frequency of Radiological Cerebral Phenotypes

The proportion of individuals with neuroimaging (magnetic resonance imaging [MRI], computed tomography, magnetic resonance angiography, or computed tomography angiography) was 74% (290/390) for *COL4A1*, 59% (73/123) for *TREX1*, 100% (44/44) for *HTRA1* ^HomZ^, 76% (31/41) for *COL4A2*, 34% (119/346) for *ADA2*, 85% (70/82) for *HTRA1* ^HetZ^, and 100% (14/14) for *CTSA*. Where neuroimaging was done, it included an MRI scan in 71% to 100% of cases. The rest of this section applies to those with neuroimaging only.

**Figure 4:**
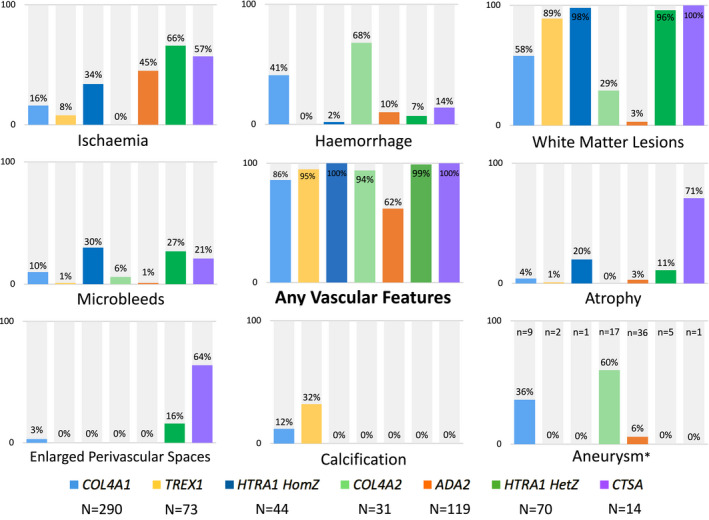
Frequency of radiological cerebral phenotypes by gene. Hemorrhage: intracerebral hemorrhage, intraventricular hemorrhage or porencephalic cysts. HetZ indicates heterozygous; HomZ, homozygous/compound heterozygous; and N, number of individuals with neuroimaging. Of those with computed tomography angiograms or magnetic resonance angiograms reported are indicated by asterisk (*).

The majority of individuals showed vascular feature(s) on neuroimaging: ≥86% for all genes except *ADA2* (62%). Figure 4 shows the proportion of individuals with specific features suggestive of vascular brain disease, and Table S2 shows the breakdown of these features by location and severity.

#### Ischemia

Presence ranged from 0% (*COL4A2*) to 66% (*HTRA1* ^HetZ^). Ischemia was the most common radiological manifestation for individuals with *ADA2* (45%). Location was reported for most individuals (80%), and as expected, where reported, was mainly in deep/lacunar areas. Most individuals (70%) had multiple lesions.

#### Intracerebral Hemorrhage

Presence ranged from 0% (*TREX1*) to 68% (*COL4A2*). It was predominantly present in individuals with *COL4A1/2*. However, ICH was also present in a small minority (7%–10%) of individuals with *HTRA1*, *ADA2*, and *CTSA*. Porencephaly was present in individuals with *COL4A1/2* only (61% and 76%, respectively) and intraventricular hemorrhage was present in individuals with *COL4A1* only (7%). Location, where reported, was mostly deep. The burden is less clear: Single lesions were common, though a minority of individuals did have multiple lesions.

#### White Matter Lesions

Presence ranged from 3% (*ADA2*) to 100% (*CTSA*). WMLs were the most common radiological manifestation for 5 of 7 genes (not *COL4A2* and *ADA2*). Location was poorly reported, though, where reported, was common in the temporal regions in several genes. Individuals with *CTSA* appear to have lesions mainly in the frontal and parietal regions (though numbers are low). The burden of WMLs, where reported, was mostly severe, though the burden was not reported well (data missing for 51% individuals). The exception to this was individuals with *HTRA1* ^HetZ^, who appear to have less severe WMLs. All individuals with *CTSA* with WMLs with known location had temporal lobe sparing.

#### Microbleeds

Presence ranged from 1% (*TREX1* and *ADA2*) to 30% (*HTRA1* ^HomZ^). Microbleeds were also common in individuals with *HTRA1* ^HetZ^ (27%). Location, where reported, was mostly deep. All individuals had multiple lesions where burden was reported.

#### Atrophy

Presence ranged from 0% (*COL4A2*) to 71% (*CTSA*). Location and burden were poorly described overall, and the low numbers make it difficult to make any conclusions.

#### Enlarged PVSs

Presence was infrequent: Enlarged PVSs were present in *COL4A1* (3%), *HTRA1* ^HetZ^ (16%), and *CTSA* (64%) individuals only.

#### Calcification

Presence was infrequent: Calcification was present in individuals with *COL4A1/2* only (12% and 32%, respectively).

#### Cerebral Aneurysm

Present in 36% (13/36) of individuals with *COL4A1*, 60% (3/5) with *COL4A2* and 6% (1/17) with *ADA2* (of those with computed tomography angiograms or magnetic resonance angiograms reported).

#### Other Radiological Cerebral Phenotypes

Individuals with *COL4A1/2* were also reported to manifest with schizencephaly (8% [24/290] of individuals with *COL4A1* and 13% [4/31] with *COL4A2*) and cerebellar atrophy (5% [14/290] of individuals with *COL4A1* and 3% [1/31] with *COL4A2*). Fifteen percent of individuals with *TREX1* (11/73) had pseudotumoral lesions.

#### Particular Patterns to Lesion Location or Severity to Help Identify Cases in Practice

**Figure 5:**
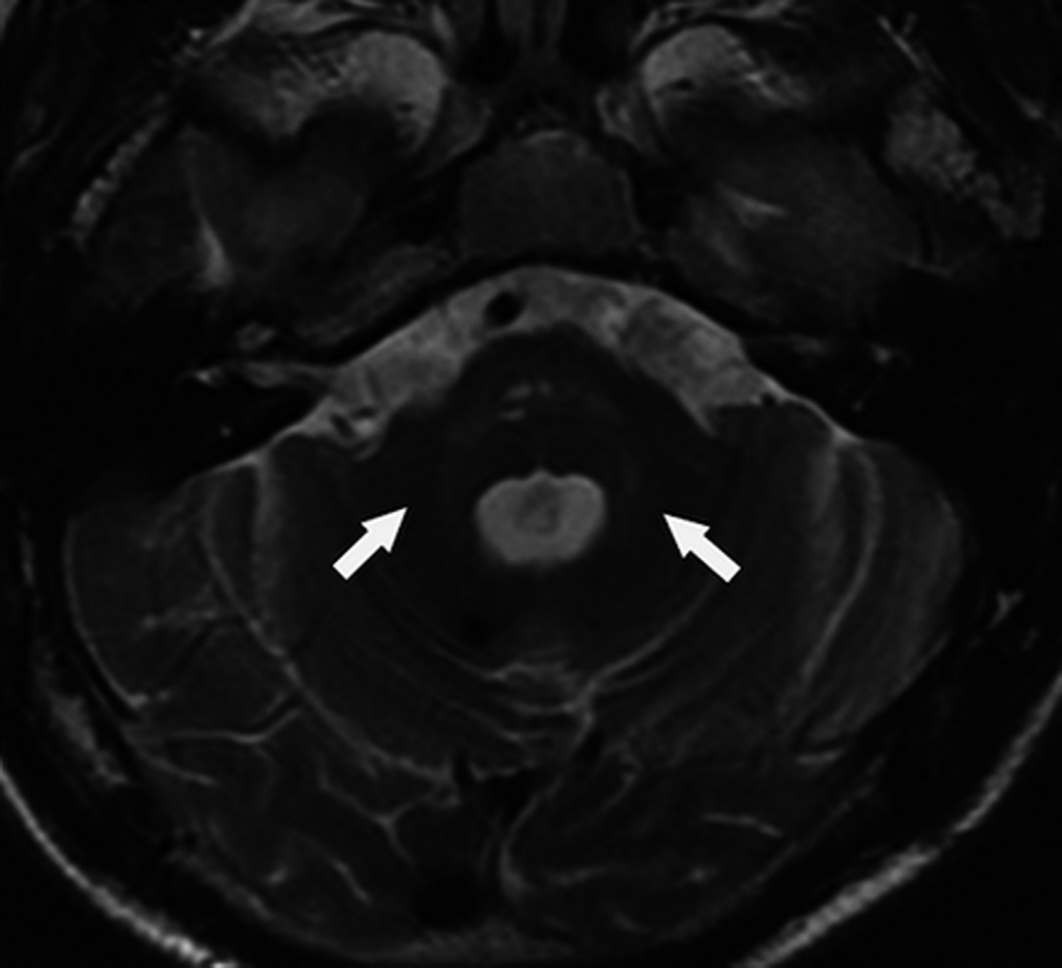
Example of the “arc sign” of the cerebellopontine peduncle on MRI imaging. Reproduced with permission from Yu et al [^22^]. Copyright © 2020 Elsevier.

**Figure 6:**
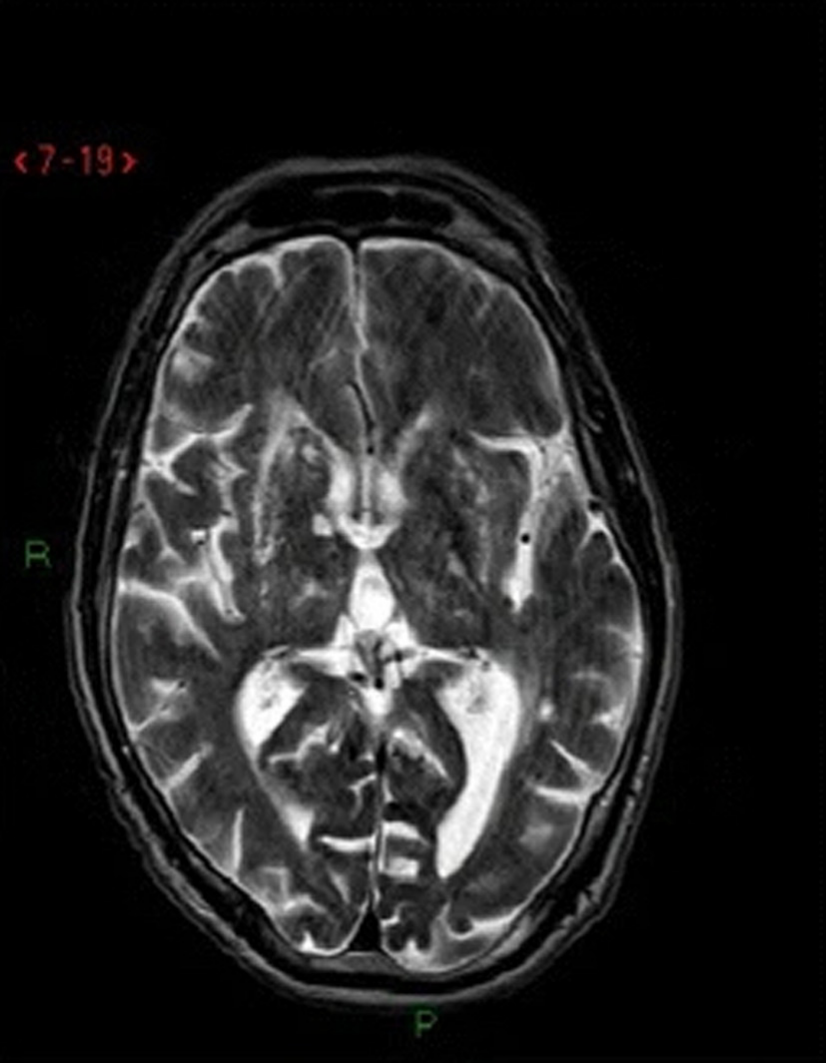
Example of “état crible” on MRI imaging. Reprinted with permission from Pati et al [^23^] Copyright 2018, Springer.

A unique feature of individuals with *HTRA1^HomZ^* was the presence of arc‐shaped hyperintense lesions from the pons to the middle cerebellar peduncles referred to as the “arc sign” (9% [4/44] of individuals) (Figure 5).^20^ A unique feature of individuals with *HTRA1^HetZ^* was the presence of dilated PVSs in the basal ganglia referred to as “status cribrosum” or “état crible” (13% [9/70] individuals) (Figure 6).^2^, ^21^ Overall, the descriptions provided were not detailed enough to identify further patterns for other genes.

### Vascular Risk Factor Stratification

**Figure 7:**
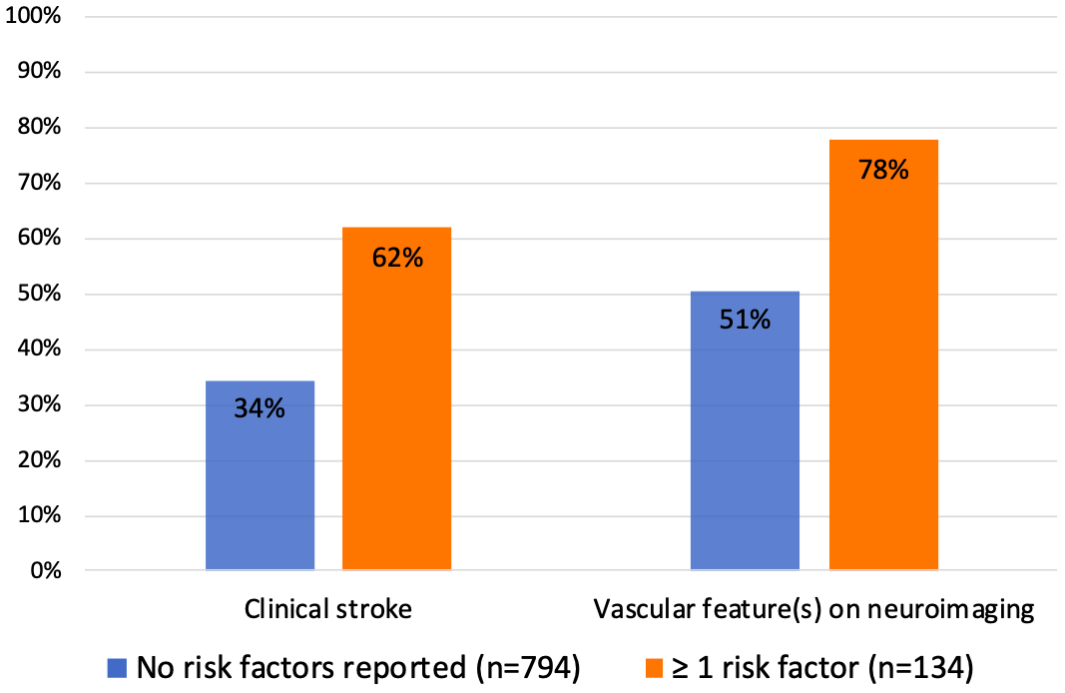
Frequency of cerebral phenotypes, stratified by presence of vascular risk factors.

Fourteen percent (134/928) of individuals across all genes were reported to have ≥1 vascular risk factors. Of these individuals, 62% (88/134) reported clinical stroke, compared with 34% (272/794) of individuals with no reported risk factors (*P*<0.01), while 78% (104/134) reported vascular features on neuroimaging, compared with 51% (401/794) of individuals with no reported risk factors (*P*<0.01) (Figure 7). The mean (median) age was 43 (48) years for those with ≥1 risk factor, and 22 (17) years for those with no reported risk factors. This analysis excludes individuals for whom data on risk factors or phenotypes were not available on an individual basis.

### Variant Pathogenicity Assessment

VEP produced results from ≥1 of its subcomponents for 15% to 66% of variants overall (SnpEff, 66%; ClinVar, 15%; SIFT, 60%; and Polymorphism Phenotyping v2, 62%), although there was substantial variability for these estimates across different genes. While the percentage of variants with supporting evidence of pathogenicity was high (81%–99%) when studying only the group of variants with data available, this appeared much lower when including all variants regardless of whether VEP was able to process them (12%–65%). Again, there was substantial variability across individual genes (Tables S3 and S4).

## DISCUSSION

Vascular changes are commonly seen on neuroimaging in individuals with rare variant(s) in cSVD genes. Where data are available, the most frequent radiological manifestations are WMLs and ischemic changes and, as expected, most lesions are deep. Common clinical phenotypes include clinical stroke, psychiatric symptoms, and, most frequently reported, cognitive decline. Overall, radiological vascular phenotypes were more common than clinical neurological phenotypes. However, when interpreting these results, it is important to bear in mind that variation in the mean age of affected individuals may explain some of the differences in phenotypes between genes (eg, increased age is a risk factor for both clinical stroke and vascular cerebral phenotypes on neuroimaging).

Both ICH and ischemic stroke were described for all cSVD genes, although the most common stroke subtype was hemorrhagic for *COL4A1/2* and ischemic for the remaining genes. Enlarged perivascular spaces were infrequently reported, which may reflect this feature being less apparent with older imaging modalities, difficult to differentiate from other lesions such as lacunes,^2^ or less commonly reported on neuroimaging.

The frequency of both clinical stroke and vascular radiological features on neuroimaging was higher for those with at least 1 vascular risk factor, compared with those with no reported risk factors. However, vascular risk factors were generally poorly reported (therefore, their presence cannot be excluded in most cases), age is highly likely to be a confounding factor, and individuals presenting with stroke/vascular radiological features are more likely to be investigated for vascular risk factors. More research is needed to understand the role for a focused effort on addressing modifiable vascular risk factors in the management of monogenic cSVDs.

We identified only 14 individuals with a putative pathogenic variant in *CTSA*. This is likely (at least partly) explained by the relatively recent description of its association with cSVD, but the small overall number of affected individuals limit the conclusions that can be drawn about its phenotype associations.

The strengths of our study are (1) a comprehensive search strategy, including foreign‐language papers and abstracts; (2) systematic data extraction following a preset spreadsheet with a comprehensive list of variables to be collected, while also allowing for novel phenotypes to be recorded; and (3) inclusion of several cSVD genes, allowing comparisons to be made across these.

This research also has some limitations. First, reporting for some variables was poor. For example, region of origin as a marker of ethnicity was frequently poorly reported and therefore often had to be assumed on the basis of information such as the location of the authors’ institute. It is possible that some true differences between ethnicities may not have been revealed because of incorrect categorization. Furthermore, individuals from African and South American regions were reported rarely (none reported in 5/7 genes; ≤2% of individuals in 2/7 genes). The understudy of these populations, which comprise over a fifth of the world population, may limit our appreciation of the breadth and frequency of phenotypes that exist. The frequency of neuroimaging reporting was also low for some genes, and it is unknown if neuroimaging was not reported because of lack of positive findings or whether it was not done at all. Second, case reports and case series have many inherent biases that are difficult to control for (eg, testing bias, publication bias, and reporting bias). In addition, the case reports included in this research appeared to lack use of a reporting structure. Current guidelines such as CARE (CAse REports)^24^, ^25^ do not work so well in the field of rare genetic diseases, so new, tailored guidelines could help improve the consistency of reporting.

The frequency profile of clinical cerebral phenotypes associated with monogenic cSVDs suggests that it is important to consider a broader spectrum of manifestations when identifying potential patients for genetic testing. Specifically, cognitive involvement appeared even more frequently than clinical stroke for several genes. Our results also show that in monogenic cSVD a radiological vascular phenotype is described more frequently than clinical cerebral phenotypes, suggesting a potential benefit of radiological screening, both for patients and for at‐risk family members.

Mancuso et al^26^, ^27^ and Guey et al^26^, ^27^ provide expert recommendations regarding indications for monogenic cSVD testing in a clinical context. Our work broadly supports these existing recommendations, including “red flag” suggestive clinical and radiological features and age of onset for each gene.

It is also notable that across several monogenic cSVDs, WMLs were commonly identified in the temporal region, a feature that has previously been associated with cerebral autosomal dominant arteriopathy with subcortical infarcts and leukoencephalopathy (caused by *NOTCH3* mutations).^28^ It is therefore important to also consider other cSVD genes in the presence of this feature.

Finally, according to OMIM (https://www.omim.org), headache is a known phenotype associated with *TREX1* rare variants, thus its high frequency in individuals with *TREX1* was expected. However, other genes associated in OMIM with headache (*COL4A1*, *ADA2*, and *HTRA1*) were not found to have a clear association with this phenotype in our review. Forty‐three percent of individuals with *CTSA* (albeit among a total of only 14 individuals) also reported headache, which is more than the expected population prevalence of 15%,^29^ suggesting a potentially novel associated phenotype. Epilepsy was another common phenotype in *COL4A1/2*, as suggested by OMIM and previous literature.^30^

VEP predicted 81% to 99% of the processed variants to have a high likelihood of being pathogenic. However, since these percentages are calculated only among variants with data available, this introduces a bias, as some variants without data (eg, synonymous single‐nucleotide polymorphisms) have a lower prior likelihood of being pathogenic. Adjusting these calculations to include all variants resulted in only 12% to 65% of variants having supporting evidence of pathogenicity, with substantial variability for results across individual genes. Also, it is possible that some variants have been submitted to ClinVar on the basis of the same case report/case series included in our review. This makes it difficult to draw robust conclusions about included variants’ pathogenicity.

The findings summarized here have potential clinical implications for the diagnosis and follow‐up of monogenic cSVDs, especially in conjunction with previous data of associated extracerebral phenotypes.^5^ Having said this, to get a more comprehensive and less biased overview of the clinical and radiological consequences of monogenic cSVDs, further work should address these same questions using a genotype‐first approach (ie, studying this in a population‐based setting and among individuals selected on the basis of carrying the variant of interest, regardless of their phenotype). The emergence of prospective population‐based studies with biosamples yielding genetic data at scale, such as the UK Biobank (https://www.ukbiobank.ac.uk), will make this possible and complement our study findings.

In summary, we found that individuals with rare variant(s) in our genes of interest appear to develop vascular features on neuroimaging. Clinical stroke and cognitive and psychiatric features are also common. The phenotype profiles appear to differ across monogenic cSVD genes, however, these results may be affected by age and other biases inherent to case reports. In the future, better characterization of associated phenotypes, as well as insights from population‐based studies, should improve our understanding of monogenic cSVD to inform genetic testing, guide clinical management, and help unravel underlying disease mechanisms.

## Sources of Funding

Dr Wardlaw: UK Dementia Research Institute Centre with funding received from Dementia Research Institute Ltd, UK Medical Research Council, Alzheimer’s Society, Alzheimer’s Research UK. Dr Rannikmäe: Rutherford fellowship MR/S004130/1. Dr Ferguson: British Heart Foundation award RE/18/5/34216, MR/S004130/1.

## Disclosures

None.

## Supporting information

Data S1
Tables S1–S4
