# Supplementary material for: Systematic Review of Cerebral Phenotypes Associated With Monogenic Cerebral Small‐Vessel Disease
Source: J Am Heart Assoc. 2022 Jun 14;11(12):e025629. doi: 10.1161/JAHA.121.025629 (PMC9238640; doi:10.1161/JAHA.121.025629)
Supplement: Supplementary file 1 — Data S1 Tables S1–S4 [file JAH3-11-e025629-s001.pdf]

SUPPLEMENTAL MATERIAL

Data S1.

Supplemental Methods: Decisions and Assumptions made when extracting data

Demographic data

- Age: sometimes specific ages weren't reported but rather an approximate age or greater/less than a particular age was provided. In these cases we took a best estimation, erring towards overestimating age in some cases so as to minimise overestimation of the burden of the disease in younger brains. For example: <1 = 0, <2 = 1, <27 = 26, ≤26 = 26, early 50s = 52, mid-40s = 45.

Clinical data

- Clinical stroke classification required reporting of symptoms, i.e. not just radiological description
- Intellectual disability was classified under developmental delay

Radiology data

- When scan findings only described 'hemosiderin deposits' we did not take it to mean a confirmed bleed or microbleed
- Cerebral matter loss in <18 year old was recorded as 'other' rather than 'atrophy'
- If a scan was described as showing 'stable findings'/'no changes' or equivalent, we marked the scan as showing the same pathology as the previous scan of the same patient
- In general, author interpretations which used words such as 'probable' or 'suggests' were taken to mean the feature was present, while author interpretations which used words such as 'possible' or 'might be' were not sufficient to consider the feature present
- We took 'periventricular gliosis' to mean white matter lesions
- We classified haemorrhage at the splenium of corpus callosum as 'deep'
- We took 'Hyperintense signal adjacent to the horn of the lateral ventricle' to mean periventricular white matter lesions
- External capsule, internal capsule, centrum semiovale and corona radiata locations qualified as deep
- Punctate hemorrhages were taken to mean brain microbleeds
- Regarding severity of white matter lesions, we assumed the following:
    - 'Severe' when described as: extensive, diffuse, severe, widespread, confluent, Fazekas score 3, disseminated
    - 'Not severe' when described as subtle, early/beginning confluent, limited, moderate, mild, weak, Fazekas score 1 or 2, punctiform
- If a scan was implied but not explicitly stated, we decided whether it was more likely a scan was done than not and assumed based on that – e.g. "haemorrhage in the right frontal area" was taken to mean a scan had been done
- We took a 'petechial spot' to mean a microbleed
- We took porencephalic cysts to be a subcategory of intracerebral haemorrhage

Search Strategy

1. CADASIL/
2. (CADASIL or "Cerebral autosomal dominant arterio$ with subcortical infarct$ and leukoencephalopathy" or (Dementia and hereditary and multi?infarct) or "Familial vascular leukoencephalopathy" or CASIL or "Cerebral arterio$ with subcortical infarct$ and leukoencephalopathy" or "Chronic familial vascular encephalopathy" or "Familial disorder with subcortical ischemic stroke$" or "Agnogenic medial arteriopathy" or "Familial Binswanger$ disease" or (cerebral and autosomal dominant and arterio$ and infarct$ and leukoencephalophy)).af.
3. (CARASIL or "Maeda$ syndrome" or "Cerebral autosomal recessive arterio$ with subcortical infarct$ and leukoencephalopathy" or ("Subcortical Vascular Encephalopathy" and Progressive) or "Cerebrovascular Disease With Thin Skin Alopecia And Disc Disease" or "Nemoto disease" or (cerebral and autosomal recessive and arterio$ and infarct$ and leukoencephalophy) or "Familial young adult onset arterio$ leukoencephalopathy with alopecia and lumbago").af.
4. ((COL4A1$ and (leukoencephalopathy or small vessel disease or autosomal dominant or infantile hemiparesis or retinal arter$ tortuosity or RATOR or PADMAL or "pontine autosomal dominant microangiopathy and leukoencephalopathy" or Walker Warburg or porencephaly 1 or "small vessel disease of the brain with or without ocular abnormalities" or BSVD)) or HANAC or (hereditary angio$ and nephropath$ and aneurysm$ and cramp$) or ((autosomal dominant or familial or hereditary) and (h?ematuria and Retinal Arter$ Tortuosity)) or ("Autosomal dominant familial porencephaly" or "Hereditary multi infarct dementia" or HEMID or hMID) or (multi-infarct dementia and Swedish) or "Nonsyndromic autosomal dominant congenital cataract").af.
5. Muscle Cramp/ and Raynaud Disease/
6. (COL4A2 and (Porencephaly or stroke or Microbleed$ or h?emorrhage or leukoencephalopathy or small vessel disease or autosomal recessive or infantile hemiparesis or retinal arter$ tortuosity)).af.
7. (RVCL or "Retinal vasculopathy with cerebral leukodystrophy" or ($retinal vascul$ and (hereditary or familial)) or ((Cerebroretinal Vasculopathy and Hereditary) or "hereditary vascular retinopathy") or "Grand-Kaine-Fulling syndrome" or HERNS or Hereditary Systemic Angiopathy or (hereditary and endotheliopathy and retin$ and nephro$ and stroke$) or (hereditary and retin$ and (raynaud$ or migraine)) or ADRVCL or (Autosomal Dominant and Retin$ and (leukodystrophy or leukoenchalopathy))).af.
8. ("Early-onset stroke and vasculopathy associated with mutations in ADA2" or (Stroke and vasc$ and ADA2) or ((deficien$ and (ADA 2 or ADA2 or adenosine deaminase-2)) or DADA2 or DADA 2 or (Vasculitis and ADA2 deficien$)) or Sneddon Syndrome or (Polyarteritis nodosa and Childhood onset)).af.

9. (CARASAL or (Cathepsin A related arteriopathy with stroke? and leukoencephalopathy)).af.
10. 1 or 2 or 3 or 4 or 5 or 6 or 7 or 8 or 9
11. (NOTCH?3 or Notch 3 or "Neurogenic locus notch homolog protein 3").af.
12. (TREX?1 or TREX 1 or "Three prime repair exonuclease 1").af.
13. (COL4A1 or COL4A2 or COL4 A1 or COL4 A2 or "COL4 A 1" or "COL4 A 2" or "COL 4 A1" or "COL 4 A2").af.
14. (Collagen and ("type IV" or "type 4") and (alpha?1 or alpha?2 or alpha 1 or alpha 2)).af.
15. Collagen Type IV/
16. (alpha?1 or alpha?2 or alpha 1 or alpha 2).af.
17. 15 and 16
18. (HTRA?1 or HTRA 1 or "HtrA serine peptidase 1" or "HtrA serine protease 1").af.
19. (CECR?1 or CECR 1 or "Cat eye syndrome critical region protein 1" or "adenosine deaminase 2" or ADA2 or ADA 2).af.
20. (FOXC?1 or FOX C1 or FOXC 1 or "FOX C 1" or "forkhead box C?1" or "Forkhead box C 1").af.
21. (PITX?2 or PITX 2 or "paired-like homeodomain 2" or "pituitary homeobox 2" or "Paired-like homeodomain transcription factor 2").af.
22. (Cathepsin?A or Cathepsin A or CathA or Cath A or CTSA).af.
23. 11 or 12 or 13 or 14 or 17 or 18 or 19 or 20 or 21 or 22
24. exp Cerebral Small Vessel Diseases/
25. exp Cerebrovascular Disorders/
26. exp stroke/
27. exp dementia, vascular/
28. Brain Diseases/
29. exp basal ganglia cerebrovascular disease/
30. exp brain ischemia/
31. exp intracranial arterial diseases/
32. exp Cerebral Hemorrhage/
33. exp intracranial hemorrhages/
34. leukomalacia, periventricular/
35. stroke, lacunar/
36. Leukoaraiosis/
37. Leukoencephalopathies/
38. White Matter/
39. Infarction/
40. ("Cerebral Small Vessel Disease?" or cerebrovascular).af.
41. (White matter hyperintensit$ or WMH$ or White matter MR hyperintensit$ or White matter magnetic resonance hyperintensit$ or Subcortical hyperintensit$ or White matter

lesion? or WML$ or Hyper intensit$ or Leukodystroph$ or Leukoaraiosis or Leukomalacia or White Matter Change? or WMC? or White Matter Disease or WMD or White matter damage or Grey matter hyperintensit$ or Brainstem hyperintensit$ or Subcortical hyperintensit$ or White matter hypoattenuation? or White matter hypodensit$ or Leukoencephalopath$).af.
42. (Subcortical infarct? or Cerebral infarct$ or Brain infarct$ or Silent brain infarct$ or Striatocapsular infarct$ or Lacunar infarct$ or Lacune? or Lacunar stroke? or Lacunar syndrome or Stroke? or Vascular lesion?).af.
43. (Microbleed? or Cerebral Microbleed or CMB? or Hypointense lesion? or Subcortical H?emorrhage or Intracerebral h?emorrhage or Cortical siderosis or Superficial siderosis).af.
44. (Perivascular space? or Virchow Robin space? or Type 3 lacune? or Etat crible).af.
45. (Brain atrophy or Cerebral atrophy or Global atrophy or Corpus callosum atrophy or Central atrophy or Mesencephalic atrophy or Hippocampal atrophy or Cortical thinning).af.
46. 24 or 25 or 26 or 27 or 28 or 29 or 30 or 31 or 32 or 33 or 34 or 35 or 36 or 37 or 38 or 39 or 40 or 41 or 42 or 43 or 44 or 45
47. 23 and 46
48. 10 or 47
49. limit 48 to humans
50. remove duplicates from 49

PRISMA 2020 Checklist

Section and Topic	Item #	Checklist item	Location where item is reported
**TITLE**			
Title	1	Identify the report as a systematic review.	p.0
**ABSTRACT**			
Abstract	2	See the PRISMA 2020 for Abstracts checklist.	p.1-2
**INTRODUCTION**			
Rationale	3	Describe the rationale for the review in the context of existing knowledge.	p.3-4
Objectives	4	Provide an explicit statement of the objective(s) or question(s) the review addresses.	p.4
**METHODS**			
Eligibility criteria	5	Specify the inclusion and exclusion criteria for the review and how studies were grouped for the syntheses.	p.5-6
Information sources	6	Specify all databases, registers, websites, organisations, reference lists and other sources searched or consulted to identify studies. Specify the date when each source was last searched or consulted.	p.4
Search strategy	7	Present the full search strategies for all databases, registers and websites, including any filters and limits used.	Suppl.
Selection process	8	Specify the methods used to decide whether a study met the inclusion criteria of the review, including how many reviewers screened each record and each report retrieved, whether they worked independently, and if applicable, details of automation tools used in the process.	p.5
Data collection process	9	Specify the methods used to collect data from reports, including how many reviewers collected data from each report, whether they worked independently, any processes for obtaining or confirming data from study investigators, and if applicable, details of automation tools used in the process.	p.6-7
Data items	10a	List and define all outcomes for which data were sought. Specify whether all results that were compatible with each outcome domain in each study were sought (e.g. for all measures, time points, analyses), and if not, the methods used to decide which results to collect.	p.6-7; Suppl.
	10b	List and define all other variables for which data were sought (e.g. participant and intervention characteristics, funding sources). Describe any assumptions made about any missing or unclear information.	p.6-7; Suppl.
Study risk of bias assessment	11	Specify the methods used to assess risk of bias in the included studies, including details of the tool(s) used, how many reviewers assessed each study and whether they worked independently, and if applicable, details of automation tools used in the process.	p.15 para2
Effect measures	12	Specify for each outcome the effect measure(s) (e.g. risk ratio, mean difference) used in the synthesis or presentation of results.	N/A
Synthesis methods	13a	Describe the processes used to decide which studies were eligible for each synthesis (e.g. tabulating the study intervention characteristics and comparing against the planned groups for each synthesis (item #5)).	p.7
	13b	Describe any methods required to prepare the data for presentation or synthesis, such as handling of missing summary statistics, or data conversions.	p.7
	13c	Describe any methods used to tabulate or visually display results of individual studies and syntheses.	p.7
	13d	Describe any methods used to synthesize results and provide a rationale for the choice(s). If meta-analysis was performed, describe the model(s), method(s) to identify the presence and extent of statistical heterogeneity, and software package(s) used.	p.7
	13e	Describe any methods used to explore possible causes of heterogeneity among study results (e.g. subgroup analysis, meta-regression).	N/A
	13f	Describe any sensitivity analyses conducted to assess robustness of the synthesized results.	N/A
Reporting bias assessment	14	Describe any methods used to assess risk of bias due to missing results in a synthesis (arising from reporting biases).	p.15 para2
Certainty assessment	15	Describe any methods used to assess certainty (or confidence) in the body of evidence for an outcome.	N/A

PRISMA 2020 Checklist

Section and Topic	Item #	Checklist item	Location where item is reported
**RESULTS**			
Study selection	16a	Describe the results of the search and selection process, from the number of records identified in the search to the number of studies included in the review, ideally using a flow diagram.	p.8, Fig. 1
	16b	Cite studies that might appear to meet the inclusion criteria, but which were excluded, and explain why they were excluded.	Fig. 1, Suppl.
Study characteristics	17	Cite each included study and present its characteristics.	Suppl.
Risk of bias in studies	18	Present assessments of risk of bias for each included study.	N/A
Results of individual studies	19	For all outcomes, present, for each study: (a) summary statistics for each group (where appropriate) and (b) an effect estimate and its precision (e.g. confidence/credible interval), ideally using structured tables or plots.	Suppl.
Results of syntheses	20a	For each synthesis, briefly summarise the characteristics and risk of bias among contributing studies.	Table 1, Fig. 2,3
	20b	Present results of all statistical syntheses conducted. If meta-analysis was done, present for each the summary estimate and its precision (e.g. confidence/credible interval) and measures of statistical heterogeneity. If comparing groups, describe the direction of the effect.	N/A
	20c	Present results of all investigations of possible causes of heterogeneity among study results.	N/A
	20d	Present results of all sensitivity analyses conducted to assess the robustness of the synthesized results.	N/A
Reporting biases	21	Present assessments of risk of bias due to missing results (arising from reporting biases) for each synthesis assessed.	p.15 para 2
Certainty of evidence	22	Present assessments of certainty (or confidence) in the body of evidence for each outcome assessed.	N/A
**DISCUSSION**			
Discussion	23a	Provide a general interpretation of the results in the context of other evidence.	p.14-17
	23b	Discuss any limitations of the evidence included in the review.	p.15
	23c	Discuss any limitations of the review processes used.	p.15-16
	23d	Discuss implications of the results for practice, policy, and future research.	p.16-17
**OTHER INFORMATION**			
Registration and protocol	24a	Provide registration information for the review, including register name and registration number, or state that the review was not registered.	p.4
	24b	Indicate where the review protocol can be accessed, or state that a protocol was not prepared.	p.4
	24c	Describe and explain any amendments to information provided at registration or in the protocol.	N/A
Support	25	Describe sources of financial or non-financial support for the review, and the role of the funders or sponsors in the review.	p.17
Competing interests	26	Declare any competing interests of review authors.	p.17
Availability of data, code and other materials	27	Report which of the following are publicly available and where they can be found: template data collection forms; data extracted from included studies; data used for all analyses; analytic code; any other materials used in the review.	Suppl.

From: Page MJ, McKenzie JE, Bossuyt PM, Boutron I, Hoffmann TC, Mulrow CD, et al. The PRISMA 2020 statement: an updated guideline for reporting systematic reviews. BMJ 2021;372:n71. doi: 10.1136/bmj.n71

For more information, visit: http://www.prisma-statement.org/

Table S1. Frequency and Subtypes of Cerebral Clinical

		COL4A1 (N=390)	*TREX1* (N=123)	*HTRA1*^{HomZ} (N=44)	*COL4A2* (N=41)	*ADA2* (N=346)	*HTRA1*^{HetZ} (N=82)	*CTSA* (N=14)
		% (n/N)						
CLINICAL STROKE	Unknown/ absent	59 (229/390)	91(112/123)	70 (31/44)	78 (32/41)	67(231/346)	48 (39/82)	50 (7/14)
	Present	41 (161/390)	9 (11/123)	30 (13/44)	22 (9/41)	33 (115/346)	52 (43/82)	50 (7/14)
	Ischaemic	15 (24/161)	82 (9/11)	54 (7/13)	0 (0/9)	53 (61/115)	53 (23/43)	71 (5/7)
	Ischaemic	15 (24/161)	73 (8/11)	46 (6/13)	11 (1/9)	55 (63/115)	44 (19/43)	43 (3/7)
	TIA	2 (3/161)	0 (0/11)	8 (1/13)	0 (0/9)	5 (6/115)	14 (6/43)	43 (3/7)
	Eye infarction	0 (0/161)	9 (1/11)	0 (0/13)	0 (0/9)	3 (4/115)	0 (0/43)	14 (1/7)
	Venous thrombosis/infarct	0 (0/161)	0 (0/11)	0 (0/13)	0 (0/9)	0 (0/115)	0 (0/43)	14 (1/7)
	Haemorrhagic	72 (116/161)	0 (0/11)	8 (1/13)	89 (8/9)	12 (14/115)	5 (2/43)	0 (0/7)
	ICH	32 (51/161)	0 (0/11)	8 (1/13)	22 (2/9)	20 (23/115)	14 (6/43)	29 (2/7)
	IVH	4 (7/161)	0 (0/11)	0 (0/13)	0 (0/9)	0 (0/115)	0 (0/43)	0 (0/7)
	Porencephalic cyst	47 (76/161)	0 (0/11)	0 (0/13)	78 (7/9)	0 (0/115)	0 (0/43)	0 (0/7)
	Ischaemic and haemorrhagic	1 (2/161)	0 (0/11)	0 (0/13)	11 (1/9)	8 (9/115)	9 (4/43)	29 (2/7)
	Unspecified/ no detail	12 (19/161)	18 (2/11)	38 (5/13)	0 (0/9)	27 (31 /115)	33 (14/43)	0 (0/7)
COGNITIVE FEATURES	Unknown/ absent	67 (262/390)	71 (87/123)	36 (16/44)	73 (30/41)	100(346/346)	44 (36/82)	36 (5/14)
	Present	33 (128/390)	29 (36/123)	64 (28/44)[#]	27 (11/41)	0 (0/346)	56 (46/82)	64 (9/14)
	Present (≥18 y)	23 (30/131)	34 (36/106)	65 (20/31)	0 (0/13)	0 (0/85)	62 (46/74)	64 (9/14)
	Dementia*	3 (4/128)<br>17 (5/30)	0 (0/36)<br>0 (0/36)	32 (9/28)<br>45 (9/20)	0 (0/11)<br>0 (0/0)	0 (0/0)<br>0 (0/0)	13 (6/46)<br>13 (6/46)	0 (0/9)<br>0 (0/9)

	Cognitive impairment- no ADL impact*	2 (2/128)<br>7 (2/30)	0 (0/36)<br>0 (0/36)	0 (0/28)<br>0 (0/20)	0 (0/11)<br>0 (0/0)	0 (0/0)<br>0 (0/0)	15 (7/46)<br>15 (7/46)	0 (0/9)<br>0 (0/9)
	Cognitive impairment- no ADL detail*	12 (15/128)<br>(22/30)	97 (35/36)<br>100 (35/36)	68 (19/28)<br>55 (11/20)	0 (0/11)<br>0 (0/0)	0 (0/0)<br>0 (0/0)	65 (30/46)<br>65 (30/46)	100 (9/9)<br>100 (9/9)
	Subjective cognitive decline*	0 (0/128)<br>73 (0/30)	0 (0/36)<br>0 (0/36)	0 (0/28)<br>0 (0/20)	0 (0/11)<br>0 (0/0)	0 (0/0)<br>0 (0/0)	7 (3/46)<br>7 (3/46)	0 (0/9)<br>0 (0/9)
	Developmental delay	83 (106/128)	0 (0/36)	0 (0/28)	100 (11/11)	0 (0/0)	0 (0/46)	0 (0/9)
PSYCHIATRIC FEATURES	Unknown/ absent	98 (382/390)	71 (87/123)	68 (30/44)	100 (41/41)	100(346/346)	78 (64/82)	43 (6/14)
	Present	2 (8/390)	29 (36/123)	32 (14/44)	0 (0/41)	0 (0/346)	22 (18/82)	57 (8/14)
	Psychosis	0 (0/8)	6 (2/36)	7 (1/14)	0 (0/0)	0 (0/0)	6 (1/18)	0 (0/8)
	Depression symptoms	25 (2/8)	17 (6/36)	64 (9/14)	0 (0/0)	0 (0/0)	67 (12/18)	88 (7/8)
	Anxiety	0 (0/8)	3 (1/36)	14 (2/14)	0 (0/0)	0 (0/0)	0 (0/18)	0 (0/8)
	Irritability/ agitation	25 (2/8)	8 (3/36)	64 (9/14)	0 (0/0)	0 (0/0)	0 (0/18)	13 (1/8)
	Emotional lability	13 (1/8)	0 (0/36)	21 (3/14)	0 (0/0)	0 (0/0)	28 (5/18)	13 (1/8)
	OCD	0 (0/8)	0 (0/36)	0 (0/14)	0 (0/0)	0 (0/0)	6 (1/18)	0 (0/8)
	Unspecified/ no detail	0 (0/8)	78 (28/36)	0 (0/14)	0 (0/0)	0 (0/0)	0 (0/18)	0 (0/8)
HEADACHE	Unknown/ absent	93 (362/390)	69 (85/123)	95 (42/44)	98 (40/41)	95 (329/346)	91 (75/82)	57 (8/14)
	Present	7 (28/390)	31 (38/123)	5 (2/44)	2 (1/41)	5 (17/346)	9 (7/82)	43 (6/14)
	Migraine	68 (19/28)	84 (32/38)	50 (1/2)	100 (1/1)	24 (4/17)	43 (3/7)	83 (5/6)
	Unspecified	32 (9/28)	16 (6/38)	50 (1/2)	0 (0/1)	76 (13/17)	57 (4/7)	17 (1/6)

HetZ=heterozygous; HomZ=homozygous/compound heterozygous; N=overall number of individuals; n=number of affected individuals; ADL=activities of daily living; #8 cases with unknown age; * second row: only individuals ≥18 years; assumed Stam *et al* cohort were all ≥18 y.

Table S2. Frequency of Vascular Radiological Cerebral Phenotypes by Location and Severity

				COL4A1 (N=290)	*TREX1* (N=73)	*HTRA1*^{HomZ} (N=44)	*COL4A2* (N=31)	*ADA2* (N=119)	*HTRA1*^{HetZ} (N=70)	*CTSA* (N=14)
				% (n/N)						
**ISCHAEMIA**	Total	Present		16 (47/290)	8 (6/73)	34 (15/44)	0 (0/31)	44 (52/119)	66 (46/70)	57 (8/14)
		Unknown/Absent		84(243/290)	92 (67/73)	66 (29/44)	100(31/31)	56 (67/119)	34 (24/70)	43 (6/14)
	Location	Supratento	Deep/ lacunar	43 (20/47)	100 (6/6)	53 (8/15)	0 (0/0)	42 (22/52)	46 (21/46)	75 (6/8)
			Cortical	2 (1/47)	0 (0/6)	0 (0/15)	0 (0/0)	2 (1/52)	2 (1/46)	25 (2/8)
			Unknown	4 (2/47)	0 (0/6)	20 (3/15)	0 (0/0)	10 (5/52)	15 (7/46)	0 (0/8)
		Infratentor	Brainstem	51 (24/47)	0 (0/6)	53 (8/15)	0 (0/0)	44 (23/52)	26 (12/46)	0 (0/8)
			Cerebellum	2 (1/47)	0 (0/6)	0 (0/15)	0 (0/0)	2 (1/52)	0 (0/46)	25 (2/8)
			Unknown	0 (0/47)	0 (0/6)	0 (0/15)	0 (0/0)	0 (0/52)	0 (0/46)	0 (0/8)
		Overall	Any deep	83 (39/47)	100 (6/6)	67 (10/15)	0 (0/0)	77 (40/52)	78 (36/46)	100 (8/8)
			No deep	2 (1/47)	0 (0/6)	0 (0/15)	0 (0/0)	0 (0/52)	0 (0/46)	0 (0/8)
			Unknown	15 (7/47)	0 (0/6)	33 (5/15)	0 (0/0)	23 (12/52)	22 (10/46)	0 (0/8)
	Burden	Single lesion		2 (1/47)	33 (2/6)	0 (0/15)	0 (0/0)	37 (19/52)	0 (0/46)	50 (4/8)
		Multiple lesions		57 (27/47)	50 (3/6)	87 (13/15)	0 (0/0)	56 (29/52)	100(46/46)	38 (3/8)

		Unknown		40 (19/47)	17 (1/6)	13 (2/15)	0 (0/0)	8 (4/52)	0 (0/46)	13 (1/8)
**HAEMORRHAGE**	Total	Present		41(118/290)	0 (0/73)	2 (1/44)	68 (21/31)	10 (12/119)	7 (5/70)	7 (1/14)
		Unknown/Absent		59(172/290)	100(73/73)	98 (43/44)	32 (10/31)	90(107/119)	93 (65/70)	93(13/14)
	Porencephaly			61 (72/118)	0 (0/0)	0 (0/1)	76 (16/21)	0 (0/12)	0 (0/5)	0 (0/1)
	IVH			7 (8/118)	0 (0/0)	0 (0/1)	0 (0/21)	0 (0/12)	0 (0/5)	0 (0/1)
	Location	Supratentorial	Deep/ lacunar	25 (29/118)	0 (0/0)	0 (0/1)	14 (3/21)	50 (6/12)	40 (2/5)	100 (1/1)
			Cortical	2 (2/118)	0 (0/0)	0 (0/1)	0 (0/21)	8 (1/12)	0 (0/5)	0 (0/1)
			Unknown	13 (15/118)	0 (0/0)	0 (0/1)	10 (2/21)	42 (5/12)	0 (0/5)	0 (0/1)
		Infratentorial	Brainstem	2 (2/118)	0 (0/0)	0 (0/1)	0 (0/21)	0 (0/12)	20 (1/5)	0 (0/1)
			Cerebellum	6 (7/118)	0 (0/0)	100 (1/1)	0 (0/21)	0 (0/12)	0 (0/5)	0 (0/1)
			Unknown	0 (0/118)	0 (0/0)	0 (0/1)	0 (0/21)	0 (0/12)	0 (0/5)	0 (0/1)
		Overall	Any deep	56 (36/64)	0 (0/0)	100 (1/1)	60 (3/5)	50 (6/12)	60 (3/5)	100 (1/1)
			No deep	3 (2/64)	0 (0/0)	0 (0/1)	0 (0/5)	8 (1/12)	0 (0/5)	0 (0/1)
			Unknown	41 (26/64)	0 (0/0)	0 (0/1)	40 (2/5)	42 (5/12)	40 (2/5)	0 (0/1)

	Burden	Single lesion		45 (53/118)	0 (0/0)	100 (1/1)	76 (16/21)	25 (3/12)	100 (5/5)	100 (1/1)
		Multiple lesions		39 (46/118)	0 (0/0)	0 (0/1)	19 (4/21)	8 (1/12)	0 (0/5)	0 (0/1)
		Unknown		16 (19/118)	0 (0/0)	0 (0/1)	5 (1/21)	67 (8/12)	0 (0/5)	0 (0/1)
**WML**	Totals	Present		58(167/290)	89 (65/73)	98 (43/44)	29 (9/31)	3 (3/119)	96 (67/70)	100(14/14)
		Unknown/Absent		42(123/290)	11 (8/73)	2 (1/44)	71 (22/31)	97 116/119)	4 (3/70)	0(0/14)
	Location	General	Periventricular only	26 (43/167)	9 (6/65)	0 (0/43)	78 (7/9)	33 (1/3)	7 (5/67)	0 (0/14)
			Deep only	5 (9/167)	2 (1/65)	14 (6/43)	0 (0/9)	33 (1/3)	24 (16/67)	0 (0/14)
			Both	14 (24/167)	2 (1/65)	21 (9/43)	0 (0/9)	0 (0/3)	25 (17/67)	93 (13/14)
			Unknown	54 (91/167)	88 (57/65)	65 (28/43)	22 (2/9)	33 (1/3)	43 (29/67)	7 (1/14)
		Region	Temporal	7 (11/167)	0 (0/65)	30 (13/43)	11 (1/9)	0 (0/3)	7 (5/67)	0 (0/14)
			Frontal	3 (5/167)	0 (0/65)	5 (2/43)	11 (1/9)	0 (0/3)	0 (0/67)	86 (12/14)
			Parietal	2 (3/167)	0 (0/65)	2 (1/43)	0 (0/9)	0 (0/3)	0 (0/67)	86 (12/14)
			Brainstem	2 (3/167)	0 (0/65)	21 (9/43)	0 (0/9)	0 (0/3)	9 (6/67)	7 (1/14)
			Unknown	89(149/167)	100(65/65)	63 (27/43)	89 (8/9)	100 (3/3)	85 (57/67)	7 (1/14)
	Burde	Severe		35 (59/167)	5 (3/65)	95 (41/43)	22 (2/9)	0 (0/3)	12 (8/67)	93 (13/14)
		Not severe		12 (20/167)	3 (2/65)	0 (0/43)	0 (0/9)	0 (0/3)	49 (33/67)	0 (0/14)

		Unknown		53 (88/167)	92 (60/65)	5 (2/43)	78 (7/9)	100 (3/3)	39 (26/67)	7 (1/14)
**MICROBLEEDS**	Total	Present		10 (29/290)	1 (1/73)	30 (13/44)	6 (2/31)	0 (0/119)	27 (19/70)	21 (3/14)
		Unknown/Absent		90(261/290)	99 (72/73)	70 (31/44)	94 (29/31)	100(119/119)	73 (51/70)	79 (11/14)
	Location	Supratentorial	Deep/ lacunar	52 (15/29)	0 (0/1)	31 (4/13)	50 (1/2)	0 (0/0)	47 (9/19)	100 (3/3)
			Cortical	3 (1/29)	0 (0/1)	8 (1/13)	0 (0/2)	0 (0/0)	0 (0/19)	0 (0/3)
			Unknown	14 (4/29)	0 (0/1)	46 (6/13)	0 (0/2)	0 (0/0)	26 (5/19)	0 (0/3)
		Infratentorial	Brainstem	21 (6/29)	0 (0/1)	31 (4/13)	0 (0/2)	0 (0/0)	16 (3/19)	33 (1/3)
			Cerebellum	10 (3/29)	0 (0/1)	0 (0/13)	0 (0/2)	0 (0/0)	11 (2/19)	33 (1/3)
			Unknown	3 (1/29)	0 (0/1)	23 (3/13)	0 (0/2)	0 (0/0)	0 (0/19)	0 (0/3)
		Overall	Any deep	69 (20/29)	0 (0/1)	62 (8/13)	50 (1/2)	0 (0/0)	53 (10/19)	100 (3/3)
			No deep	0 (0/29)	0 (0/1)	0 (0/13)	0 (0/2)	0 (0/0)	0 (0/19)	0 (0/3)
			Unknown	31 (9/29)	100 (1/1)	38 (5/13)	50 (1/2)	0 (0/0)	47 (9/19)	0 (0/3)
	Burden	Single lesion		14 (4/29)	0 (0/1)	0 (0/13)	0 (0/2)	0 (0/0)	0 (0/19)	33 (1/3)
		Multiple lesions		76 (22/29)	100 (1/1)	85 (11/13)	100 (2/2)	0 (0/0)	100 19/19)	67 (2/3)
		Unknown		10 (3/29)	0 (0/1)	15 (2/13)	0 (0/2)	0 (0/0)	0 (0/19)	0 (0/3)

**CEREBRAL ATROPHY**	Total	Present	4 (12/290)	1 (1/73)	20 (9/44)	0 (0/31)	3 (4/119)	11 (8/70)	71 (10/14)
		Unknown/Absent	96(278/290)	99 (72/73)	80 (35/44)	100(31/31)	97 (115/119)	89 (62/70)	29 (4/14)
	Location	Global	25 (3/12)	0 (0/1)	0 (0/9)	0 (0/0)	25 (1/4)	25 (2/8)	0 (0/10)
		Focal	42 (5/12)	0 (0/1)	11 (1/9)	0 (0/0)	25 (1/4)	50(4/8)	10 (1/10)
		Unknown	33 (4/12)	100 (1/1)	89 (8/9)	0 (0/0)	50 (2/4)	25 (2/8)	90 (9/10)
	Burden	Severe	42 (5/12)	0 (0/1)	0 (0/9)	0 (0/0)	0 (0/4)	0 (0/8)	0 (0/10)
		Not severe	0 (0/12)	100 (1/1)	11 (1/9)	0 (0/0)	25 (1/4)	50 (4/8)	90 (9/10)
		Unknown	58 (7/12)	0 (0/1)	89 (8/9)	0 (0/0)	75 (3/4)	50 (4/8)	10 (1/10)
**CALCIFICATION**	Total	Present	12 (34/290)	32 (23/73)	0 (0/44)	0 (0/31)	0 (0/119)	0 (0/70)	0 (0/14)
		Unknown/Absent	88(256/290)	68 (50/73)	100(44/44)	100(31/31)	100(119/119)	100(70/70)	100(14/14)
**ENLARGED PVS**	Total	Present	3 (8/290)	0 (0/73)	0 (0/44)	0 (0/31)	0 (0/119)	16 (11/70)	64 (9/14)
		Unknown/Absent	97(282/290)	100(73/73)	100(44/44)	100(31/31)	100(119/119)	84 (59/70)	36 (5/14)
**CEREBRAL ANEURYSM**	Total	Present	36 (13/36)	0 (0/1)	0 (0/9)	60 (3/5)	6 (1/17)	0 (0/2)	0 (0/1)
		Unknown/Absent	64 (23/36)	100 (1/1)	100 (9/9)	40 (2/5)	94 (16/17)	100 (2/2)	100 (1/1)

HetZ=heterozygous; HomZ=homozygous/compound heterozygous; N=overall number of individuals with neuroimaging; n=number of affected individuals; WML=white matter lesions(s); PVS=perivascular space(s);

Table S3. Variant Effect Predictor Output Summary

Number of variants							% variants with info	% pathogenic* among variants with data	% pathogenic* among all variants
**VARIANT IMPACT/CLASSIFICATION OF SEVERITY (SNPEff)**									
	no info	low	moderate*	high*					
HTRA1	7	0	35	11			87%	100%	87%
ADA2	43	3	24	18			51%	93%	48%
COL4A1	43	0	88	23			72%	100%	72%
COL4A2	1	0	14	1			94%	100%	94%
TREX1	21	0	2	8			32%	100%	32%
CTSA	1	0	0	0			0%	0%	0%
Total	116	3	163	61			66%	99%	65%
**CLINICAL SIGNIFICANCE (ClinVar)**									
	no info	uncertain clinical significance	benign	likely benign	likely pathogenic*	pathogenic*			
HTRA1	30	3	0	0	5	15	43%	87%	38%
ADA2	76	2	1	0	6	3	14%	75%	10%
COL4A1	150	1	0	0	0	3	3%	75%	2%
COL4A2	5	0	0	3	3	5	69%	73%	50%
TREX1	29	0	0	0	1	1	6%	100%	6%
CTSA	1	0	0	0	0	0	0%	0%	0%
Total	291	6	1	3	15	27	15%	81%	12%
**IMPACT ON PROTEIN FUNCTION (SIFT)**									
	no info	tolerated	deleterious*						
HTRA1	18	1	34				66%	97%	64%

ADA2	43	4	41				51%	91%	47%
COL4A1	46	9	99				70%	92%	64%
COL4A2	1	2	13				94%	87%	81%
TREX1	29	1	1				6%	50%	3%
CTSA	1	0	0				0%	0%	0%
Total	138	17	188				60%	92%	55%
**IMPACT ON PROTEIN STRUCTURE AND FUNCTION (PolyPhen-2)**									
	no info	benign	possibly damaging*	probably damaging*					
HTRA1	18	0	4	31			66%	100%	66%
ADA2	43	5	1	39			51%	89%	45%
COL4A1	40	2	15	97			74%	98%	73%
COL4A2	1	0	4	11			94%	100%	94%
TREX1	29	2	0	0			6%	0%	0%
CTSA	1	0	0	0			0%	0%	0%
Total	132	9	24	178			62%	96%	59%

*category considered to provide supporting evidence for pathogenicity; SnpEff classifies each variant in one of the following output categories: high impact (variant is assumed to have a disruptive impact in the protein, probably causing protein truncation, loss of function or triggering nonsense mediated decay), moderate impact (non-disruptive variant that might change protein effectiveness), and low impact (variant assumed to be mostly harmless or unlikely to change protein behaviour). The 'modifier' category is taken to represent no information about these categories; ClinVar assigns each variant as pathogenic, likely pathogenic, likely benign, benign, or of uncertain clinical significance; SIFT predicts whether an amino acid substitution is likely to affect protein function based on sequence homology and the physico-chemical similarity between the alternate amino acids, concluding with a qualitative prediction if a variant is deleterious or tolerated; PolyPhen-2 predicts the effect of an amino acid substitution on the structure and function of a protein using sequence homology, 3D structures where available, and a number of other databases and tools. It classifies each variant as probably damaging, possibly damaging or benign.

TABLE S4. Variant Effect Predictor outputs per gene A. *HTRA1*

Genetic mutation	Protein change	Variant information
c.589C>T	p.R197X	Stop gained, likely deleterious, high impact. Pathogenic
c.865C>T	p.Q289X	Stop gained, likely deleterious, high impact. Pathogenic
c.1108C>T	p.R370X	Stop gained, high impact variant. Pathogenic/likely pathogenic
c.904C>T	p.R302X	Stop gained, high impact variant. Likely pathogenic
c.502A.T	p.K168ter	Stop gained, high impact variant
c.847G>T	p.G283Ter	Stop gained, high impact variant
c.983C>A	p.S328*	Stop gained, high impact variant
c.1005+1G>T		Splice donor variant, high impact
c.971A>C	p.N324T	Missense variant, possible splice region variant with moderate impact. Probably damaging to protein structure and conflicting evidence of tolerated/deleterious to protein function. Likely pathogenic
c.754G>A	p.A252T	Missense variant, non-coding exon variant with moderate impact. Possibly damaging to protein structure and deleterious to protein function. Pathogenic
c.956C>T	p.T319I	Missense variant, moderate impact. Probably damaging to protein structure and likely to have deleterious effect on protein function
c.451C>A	p.Q151K	Missense variant, moderate impact and potential modifier of both upstream and downstream gene regulation (ARMS2), and lncRNA. Probably damaging to protein structure and likely to have deleterious effect on protein function. Uncertain clinial significance
c.359G>A	p.G120D	Missense variant, moderate impact and potential modifier of both upstream and downstream gene regulation (ARMS2), and lncRNA. Probably damaging to protein structure and likely to have deleterious effect on protein function. Likely pathogenic
c.361A>C	p.S121R	Missense variant, moderate impact and potential modifier of both upstream and downstream gene regulation (ARMS2), and lncRNA. Probably damaging to protein structure and likely to have deleterious effect on protein function

c.397C>G	p.R133G	Missense variant, moderate impact and potential modifier of both upstream and downstream gene regulation (ARMS2), and lncRNA. Possibly damaging to protein structure but tolerated by protein function
c.367G>T	p.A123S	Missense variant, moderate impact and potential modifier of both upstream and downstream gene regulation (ARMS2), and lncRNA. Only possibly damaging to protein structure and likely to have deleterious effect on protein function
c.821G>A	p.R274Q	Missense variant with moderate impact. Probably/possibly damaging to protein structure and deleterious/some reports of tolerated to protein function. Pathogenic
c.496C>T	p.R166C	Missense variant with moderate impact. Probably/possibly damaging to protein structure and deleterious to protein function
c.517G>A	p.A173T	Missense variant with moderate impact. Probably/possibly damaging to protein structure and deleterious to protein function
c.517G>C	p.A173P	Missense variant with moderate impact. Probably/possibly damaging to protein structure and deleterious to protein function
c.856T>G	p.F286V	Missense variant with moderate impact. Probably/possibly damaging to protein structure and deleterious to protein function
c.854C>A	p.P285Q	Missense variant with moderate impact. Probably damaging to protein structure and deleterious to protein function. Uncertain clinical significance/pathogenic
c.854C>T	p.P285L	Missense variant with moderate impact. Probably damaging to protein structure and deleterious to protein function. Uncertain clinical significance/pathogenic
c.616G>A	p.G206R	Missense variant with moderate impact. Probably damaging to protein structure and deleterious to protein function. Uncertain clinical significance
c.961G>A	p.A321T	Missense variant with moderate impact. Probably damaging to protein structure and deleterious to protein function. Uncertain clinical significance
c.1091T>C	p.L364P	Missense variant with moderate impact. Probably damaging to protein structure and deleterious to protein function. Pathogenic
c.497G>T	p.R166L	Missense variant with moderate impact. Probably damaging to protein structure and deleterious to protein function. Pathogenic
c.614C>G	p.S205C	Missense variant with moderate impact. Probably damaging to protein structure and deleterious to protein function. Pathogenic

c.852C>A	p.S284R	Missense variant with moderate impact. Probably damaging to protein structure and deleterious to protein function. Pathogenic
c.883G>A	p.G295R	Missense variant with moderate impact. Probably damaging to protein structure and deleterious to protein function. Pathogenic
c.889G>A	p.V297M	Missense variant with moderate impact. Probably damaging to protein structure and deleterious to protein function. Pathogenic
c.536T>A	p.I179N	Missense variant with moderate impact. Probably damaging to protein structure and deleterious to protein function. Likely pathogenic
c.827G>C	p.G276A	Missense variant with moderate impact. Probably damaging to protein structure and deleterious to protein function. Likely pathogenic
c.1021G>A	p.G341J	Missense variant with moderate impact. Probably damaging to protein structure and deleterious to protein function.
c.524T>A	p. V175E	Missense variant with moderate impact. Probably damaging to protein structure and deleterious to protein function
c.527T>C	p.V176A	Missense variant with moderate impact. Probably damaging to protein structure and deleterious to protein function
c.646 G>A	p.V216 M	Missense variant with moderate impact. Probably damaging to protein structure and deleterious to protein function
c.847G>A	p.G283R	Missense variant with moderate impact. Probably damaging to protein structure and deleterious to protein function
c.848G>A	p.G283E	Missense variant with moderate impact. Probably damaging to protein structure and deleterious to protein function
c.850A>G	p.S284G	Missense variant with moderate impact. Probably damaging to protein structure and deleterious to protein function
c.905G>A	p.R302Q	Missense variant with moderate impact. Probably damaging to protein structure and deleterious to protein function
c.1348G>C	p.D450H	Missense variant with moderate impact. Only possibly damaging to protein structure and deleterious to protein function.

c.184-185del		Intronic variant, with possible impact on both upstream and downsteam gene regulation, ARMS2. Possible influence on lncRNA.
c.830_831delAG	p.E277Vfs	Intronic variant with possible influence on upstream gene
c.126delG	p.E42fs	Frameshift variant with high impact. Pathogenic
c.543delT	p.A182Pfs*33	Frameshift mutation with high impact. Potentially leading to premature stop. Pathogenic
c.739delG	p.E247Rfs	Frameshift mutation with high impact. Potentially leading to premature stop
c.958G>A	p.D320N	Missense variant with moderate impact. Probably damaging to protein structure and deleterious to protein function.

B. *ADA2*

**Genetic mutation**	**Protein change**	**Variant information**
c.982G>A	p.E328K	Missense variant with potential impact on upstream and downstream gene regulation, possible 3'UTR variant involved in nonsense-mediated decay. Possible impact on processing of pseudogene, and CTCF binding site. Probably damaging/benign to protein structure and likely deleterious to protein function/potentially tolerated.
c.138/144delG		5'UTR variant, intronic variant with possible impact on regulation of upstream gene through processing of pseudogene/nonsense mediated decay
c.37_39del	p.K13del	5'UTR variant, intronic variant with possible impact on regulation of upstream gene through processing of pseudogene/nonsense mediated decay
c.143_144insG	p.R49Afs*13	Frameshift variant with high impact, possible impact on both upstream and downstream gene regulation. Likely pathogenic
c.144 dup	p.R49Afs*13	Frameshift variant with high impact, possible impact on both upstream and downstream gene regulation. Likely pathogenic
c.144_145ins		Frameshift variant with high impact, possible impact on both upstream and downstream gene regulation. Likely pathogenic
c.144del	p.R49Gfs*4	Frameshift variant with high impact, possible impact on both upstream and downstream gene regulation. Likely pathogenic

c.144delG	p.R49fs	Frameshift variant with high impact, possible impact on both upstream and downstream gene regulation. Likely pathogenic
c.144dupG	p.R49fs	Frameshift variant with high impact, possible impact on both upstream and downstream gene regulation. Likely pathogenic
c.629delT		Frameshift variant, high impact with potential impact on both upstream and downstream genes.
c.427del	p.I143Sfs*41	Frameshift variant, high impact. Impact on nonsense mediated decay transcript processing. Possible impact on downstream genes
c.1447_1451del	p.S483Pfs*5	Intronic variant, possible impact on transcript processing
c.680- 681delAT		Intronic variant, possible impact on transcript processing
c.973-?_1081+?del	p.V325Tfs*7	Intronic variant, possible retained intron. Could have impact on both upstream, downstream genes and nonsense medicated decay transcript processing.
c.972+3A>G		Intronic, splice region variant with low impact. Possible retained intron and impact on nonsense mediated decay transcript processing
c.326C>A	p.A109D	Missense variant with moderate impact, possible 5'UTR variant. Probably damaging to protein structure and deleterious to protein function.
c.336C>A	p.H112Q	Missense variant with moderate impact, possible 5'UTR variant. Probably damaging to protein structure and deleterious to protein function.
c.336C>G	p.H112Q	Missense variant with moderate impact, possible 5'UTR variant. Probably damaging to protein structure and deleterious to protein function.
c.336G>C	p.H112Q	Missense variant with moderate impact, possible 5'UTR variant. Probably damaging to protein structure and deleterious to protein function.
c.962G>A	p.G321E	Missense variant with moderate impact. Probably damaging to protein structure and deleterious to protein function
c.133C>T	p.A45T	Missense variant with moderate impact. Probably damaging to protein structure and deleterious to protein function.
c.1358A>G	p.Y453C	Missense variant with moderate impact. Probably damaging to protein structure and deleterious to protein function.
c.385A>C	p.T129P	Missense variant with moderate impact. Probably damaging to protein structure and deleterious to protein function.

c.932T>G	p.L311R	Missense variant with moderate impact. Probably damaging to protein structure and deleterious to protein function. Pathogenic.
c.1352T>G	p.L451W	Missense variant with potential impact on downstream gene regulation, possible 3'UTR variant involved in nonsense-mediated decay. Probably damaging to protein structure and likely deleterious to protein function.
c.1353G>T	p.L451F	Missense variant with potential impact on downstream gene regulation, possible 3'UTR variant involved in nonsense-mediated decay. Probably damaging to protein structure and likely deleterious to protein function.
c.1360G>C		Missense variant with potential impact on downstream gene regulation, possible 3'UTR variant involved in nonsense-mediated decay. Probably damaging to protein structure and likely deleterious to protein function.
c.1373T>A	p.V458D	Missense variant with potential impact on downstream gene regulation, possible 3'UTR variant involved in nonsense-mediated decay. Probably damaging to protein structure and likely deleterious to protein function.
c.1223G>A	p.C408Y	Missense variant with potential impact on downstream gene regulation, possible 3'UTR variant involved in nonsense-mediated decay. Probably damaging to protein structure and likely deleterious to protein function.
c.1348G>T	p.G450C	Missense variant with potential impact on downstream gene regulation, possible 3'UTR variant involved in nonsense-mediated decay. Probably damaging to protein structure and likely deleterious to protein function.
c.1367A>G	p.Y456C	Missense variant with potential impact on downstream gene regulation, possible 3'UTR variant involved in nonsense-mediated decay. Probably damaging to protein structure and likely deleterious to protein function.Pathogenic
c.1065C>A	p.F355L	Missense variant with potential impact on upstream and downstream gene regulation, possible 3'UTR variant involved in nonsense-mediated decay. Possible impact on processing of pseudogene. Benign protein structure and tolerated by protein function.
c.1052T>A	p.L351Q	Missense variant with potential impact on upstream and downstream gene regulation, possible 3'UTR variant involved in nonsense-mediated decay. Possible impact on processing of pseudogene. Probably damaging to protein structure and likely deleterious to protein function.

c.1057T>C	p.Y353H	Missense variant with potential impact on upstream and downstream gene regulation, possible 3'UTR variant involved in nonsense-mediated decay. Possible impact on processing of pseudogene. Probably damaging to protein structure and likely deleterious to protein function.
c.1069G>A	p.A357T	Missense variant with potential impact on upstream and downstream gene regulation, possible 3'UTR variant involved in nonsense-mediated decay. Possible impact on processing of pseudogene. Probably damaging to protein structure and likely deleterious to protein function.
c.1072G>A	p.G358R	Missense variant with potential impact on upstream and downstream gene regulation, possible 3'UTR variant involved in nonsense-mediated decay. Possible impact on processing of pseudogene. Probably damaging to protein structure and likely deleterious to protein function.
c.1078A>G	p.T360A	Missense variant with potential impact on upstream and downstream gene regulation, possible 3'UTR variant involved in nonsense-mediated decay. Possible impact on processing of pseudogene. Probably damaging to protein structure and likely deleterious to protein function.
c.140G>C	p.G47A	Missense variant, moderate impact and potential modifier of downstream gene regulation. Probably damaging to protein structure and likely to have deleterious effect on protein function
c.278T>C	p.I93T	Missense variant, moderate impact and potential modifier of downstream gene regulation. Probably damaging to protein structure and likely to have deleterious effect on protein function
c.506C>T	p.R169Q	Missense variant, moderate impact and potential modifier of downstream gene regulation. Probably damaging to protein structure and likely to have deleterious effect on protein function.
c.506G>A	R169Q	Missense variant, moderate impact and potential modifier of downstream gene regulation. Probably damaging to protein structure and likely to have deleterious effect on protein function.
c.533T>C	p.F178S	Missense variant, moderate impact and potential modifier of downstream gene regulation. Probably damaging to protein structure and likely to have deleterious effect on protein function. Possible retained intron.
c.139G>T	p.G47W	Missense variant, moderate impact and potential modifier of upstream and downstream gene regulation, possible impact on processing of pseudogene (FAM32BP). Probably damaging to protein structure and likely to have deleterious effect on protein function.Pathogenic
c.563T>C	p.L188P	Missense variant, moderate impact and potential modifier of upstream and downstream gene regulation. Probably damaging to protein structure and likely to have deleterious effect on protein function

c.578C>T	p.P193L	Missense variant, moderate impact and potential modifier of upstream and downstream gene regulation. Probably damaging to protein structure and likely to have deleterious effect on protein function.
c.139G>A	p.G47R	Missense variant, moderate impact and potential modifier of upstream and downstream gene regulation. Probably damaging to protein structure and likely to have deleterious effect on protein function. Conflicting evidence on clinical significance
c.650T>A	p.V217D	Missense variant, moderate impact and potential modifier of upstream gene regulation. Probably damaging to protein structure and likely to have deleterious effect on protein function
c.712G>A	p.D238N	Missense variant, moderate impact and potential modifier of upstream gene regulation. Probably damaging to protein structure and likely to have deleterious effect on protein function
c.872C>T	p.S291L	Missense variant, moderate impact and potential modifier of upstream gene regulation. Probably damaging to protein structure and likely to have deleterious effect on protein function.
c.620T>C		Missense variant, moderate impact and potential modifier of upstream gene regulation. Probably damaging to protein structure and likely to have deleterious effect on protein function. Uncertain clinical significance
c.791G>C	p.W264S	Missense variant, moderate impact and potential modifier of upstream gene regulation. Probably damaging/benign to protein structure and could have deleterious/tolerated impact on protein function. May cause retained intron.
c.1110C>A	p.N370K	Missense variant, moderate impact, possible 3'UTR variant involved in nonsense mediated decay. Probably damaging to protein structure and likely to have deleterious effect on protein function.
c.1445A>G		Missense variant, splice variant with potential impact on downstream gene regulation, possible 3'UTR variant involved in nonsense-mediated decay. Probably damaging to protein structure and likely deleterious to protein function.
c.1226C>A		Missense variant, splice variant with potential impact on downstream gene regulation, possible 3'UTR variant involved in nonsense-mediated decay. Probably damaging to protein structure and likely deleterious to protein function.
c.752C>T	p.P251L	Missense variant, splice region variant with moderate impact. Possibly damaging to protein structure and tolerated by protein function.
c.424G>A	p.G142S	Missense variant. Change tolerated by protein function, benign impact on protein structure

c.25C>T	p.R9W	Missense variant. Deleterious (but some evidnec of low confidence in finding) to protein function, benign impact on protein structure
c.2T>C	p.M1T	Missense variant. Deleterious (but some evidnec of low confidence in finding) to protein function, benign impact on protein structure
c.73G>T	p.G25C	Missense, splice region variant. Change tolerated by protein function and has benign impact on protein structure
c.882 -2A>G		Splice acceptor variant, high impact . Also potential impact on upstream gene regulation
c.973 -1G>A		Splice acceptor variant, high impact . Also potential regulatory region variant altering TF binding site. Could impact upstream gene regulation (RPL32P5)
c.973 -2A>G		Splice acceptor variant, high impact, may result in retained intron, could impact nonsense mediated decay . Also potential regulatory region variant altering TF binding site. Could impact upstream and downstream gene regulation (RPL32P5)
c.973-2A>G		Splice acceptor variant, high impact, may result in retained intron, could impact nonsense mediated decay . Also potential regulatory region variant altering TF binding site. Could impact upstream and downstream gene regulation (RPL32P5).
c.542+1G>A		Splice donor variant with high impact. Possible impact on nonsense mediated decay
c.753+2T>A		Splice donor variant with high impact. Possible retained intron
c.753G>A		Splice region variant with low impact. May influence downstream and upstream gene regulation.
c.781delinsCCATA	p.D261Pfs*2	Stop gained, frameshift variant with high impact
c.1196G>A	p.W399*	Stop gained, high impact
c.794C>G	p.Q265X	Stop gained, high impact variant. Possible impact on upstream gene regulation
c.916C>T	p.R306*	Stop gained, high impact variant. Possible impact on upstream gene regulation.
c.660C>A	p.Y220X	Stop gained, high impact variant. Possible impact on upstream gene regulation. Benign.
c.47+2T>C		Synonymous, intron variant with low impact. Potential retained intron

C. *COL4A1*

Genetic mutation	Protein change	Variant information
c.*35C>A		3' UTR variant, regulatory region variant
c.*31G>T		3'UTR variant, regulatory region variant

c.*32G>A		3'UTR variant, regulatory region variant
c.*32G>T		3'UTR variant, regulatory region variant
c.*33T>A		3'UTR variant, regulatory region variant
c.-2C>T		5'UTR variant, with possible impact on upstream gene regulation
c.2545G>T	p.G808V	Evidence of stop gained, high impact
c.2424delT	p.P810fs	Frameshift mutation with high impact. Potentially leading to premature stop
c.2931dupT	p.G978WfsX15	Frameshift mutation with high impact. Potentially leading to premature stop
c.3702delC	p. G1236*	Frameshift mutation with high impact. Potentially leading to premature stop
c.2085del	p.G696fs	Frameshift mutation with high impact. Potentially leading to premature stop. Pathogenic.
c.1121-18G>A		Intronic variant possibly leading to retained intron
c.2645_2646delinsAA	p.G882E	Intronic variant potentially leading to retained intron
c.3877-30C>A		Intronic variant with possible impact on upstream gene regulation. Intron retained
c.4582<br>-4586<br>dupCCCATG ins.		Intronic variant, retained intron. Likely deleterious and probably damaging. Possible impact on upstream gene regulation
c.4642T>G	p.C1548G	Missense & splice region variant with low to moderate effect. Likely to impact protein function and probably damaging
c.2969G>A	p.G990E	Missense variant and splice region variant. May result in retained intron. Possible modifier of downstream gene regulation. Likely deleterious and probably damaging.
c.2969G>T	p.G990V	Missense variant and splice region variant. May result in retained intron. Possible modifier of downstream gene regulation. Likely deleterious and probably damaging.
c.3200G>A	p. G1067E	Missense variant and splice region variant. May result in retained intron. Possible modifier of downstream gene regulation. Likely deleterious and probably damaging.
c.3200G>C	p.G1067A	Missense variant and splice region variant. May result in retained intron. Possible modifier of downstream gene regulation. Likely deleterious and probably damaging.
c.3770G>C	p.G1257E	Missense variant in possible regulatory region. Likely deleterious and probably damaging
c.3796G>C	p.G1266R	Missense variant in possible regulatory region. Likely deleterious and probably damaging
c.3832G>T	p.G1278S	Missense variant in possible regulatory region. Likely deleterious and probably damaging. Uncertain clinical significance

c.3245G>A	p.G1082E	Missense variant with moderate impact and possible modifier of downstream gene regulation. Likely deleterious and possibly damaging
c.3280G>C	p.G1094R	Missense variant with moderate impact and possible modifier of downstream gene regulation. Likely deleterious and possibly damaging
c.1249G>C	p.G417R	Missense variant with moderate impact. Benign impact on protein structure and deleterious to protein function
c.3997G>A	p.D1333N	Missense variant with moderate impact. Conflicting evidence of effect on protein function, potentially tolerated/potentially deleterious
c.3592G>A	p.G1198R	Missense variant with moderate impact. Likely deleterious and probably damaging
c.3620G>T	p.G1207V	Missense variant with moderate impact. Likely deleterious and probably damaging
c.3656G>A	p. G1219E	Missense variant with moderate impact. Likely deleterious and probably damaging
c.3671C>T	p.P1224L	Missense variant with moderate impact. Likely deleterious and probably damaging
c.3704A>G	p.K1235R	Missense variant with moderate impact. Likely deleterious and probably damaging
c.3706G>A	p.G1236R	Missense variant with moderate impact. Likely deleterious and probably damaging
c.3707G>A	p. G1237E	Missense variant with moderate impact. Likely deleterious and probably damaging
c.3712C>T	p.R1238C	Missense variant with moderate impact. Likely deleterious and probably damaging
c.3505G>A	p. G1169S	Missense variant with moderate impact. Possible splice region variant, with potential impact on downstream gene regulation. Likely deleterious and probably damaging
c.2512A>G	p.M838V	Missense variant with moderate impact. Possibly damaging to protein structure and deleterious to protein function
c.3389G>A	p.G1130D	Missense variant with moderate impact. Potentially modifies upstream and downstream gene regulation. Likely deleterious and probably damaging
c.4088 G > A	p.G1363D	Missense variant with moderate impact. Probably damaging and deleterious to protein function
c.1502G>A		Missense variant with moderate impact. Probably damaging to protein structure and deleterious to protein function
c.1528G>A	p.G510R	Missense variant with moderate impact. Probably damaging to protein structure and deleterious to protein function
c.1583G>A	p.G528E	Missense variant with moderate impact. Probably damaging to protein structure and deleterious to protein function

c.1619A>G	p.K540R	Missense variant with moderate impact. Probably damaging to protein structure and deleterious to protein function
c.2008G>A	p.G670R	Missense variant with moderate impact. Probably damaging to protein structure and deleterious to protein function
c.2045G>T	p. G682V	Missense variant with moderate impact. Probably damaging to protein structure and deleterious to protein function
c.2063G>A	p.G688D	Missense variant with moderate impact. Probably damaging to protein structure and deleterious to protein function
c.2078G>A	p.G693E	Missense variant with moderate impact. Probably damaging to protein structure and deleterious to protein function
c.2086G>A	p.G696S	Missense variant with moderate impact. Probably damaging to protein structure and deleterious to protein function
c.2086G>T	p.G696C	Missense variant with moderate impact. Probably damaging to protein structure and deleterious to protein function
c.2132G>A	p.G711E	Missense variant with moderate impact. Probably damaging to protein structure and deleterious to protein function
c.2159G>A	p.G720D	Missense variant with moderate impact. Probably damaging to protein structure and deleterious to protein function
c.2168G>A	p. G723E	Missense variant with moderate impact. Probably damaging to protein structure and deleterious to protein function
c.2504G>A	p.G835E	Missense variant with moderate impact. Probably damaging to protein structure and deleterious to protein function
c.625G>A	p. G209S	Missense variant with moderate impact. Probably damaging to protein structure and deleterious to protein function
c.634G>A	p.G212S	Missense variant with moderate impact. Probably damaging to protein structure and deleterious to protein function
c.1493G>A	p.G498D	Missense variant with moderate impact. Probably damaging to protein structure and deleterious to protein function. Pathogenic.

c.1493G>T	p.G498V	Missense variant with moderate impact. Probably damaging to protein structure and deleterious to protein function. Pathogenic.
c.3383T>A	p.I1128N	Missense variant with moderate impact. Substitution seems to be tolerated by protein function but probably damaging to protein structure
c.3715G>A	p.G1239R	Missense variant with possible impact on upstream gene regulation. Probably damaging variant
c.3941G>T	p.G1314V	Missense variant with possible impact on upstream gene regulation. Probably damaging variant
c.3976G>A	p.G1326R	Missense variant with possible impact on upstream gene regulation. Probably damaging variant
c.3995G>A	p.G1332D	Missense variant with possible impact on upstream gene regulation. Probably damaging variant
c.4031G>C	p.G1344A	Missense variant with possible impact on upstream gene regulation. Probably damaging variant
c.4105G>C	p.G1369R	Missense variant with possible impact on upstream gene regulation. Probably damaging variant
c.4213G>A	p.G1405S	Missense variant with possible impact on upstream gene regulation. Probably damaging variant
c.1801G>A	p. G601S	Missense variant, moderate impact and potential modifier of downstream gene regulation. Only possibly damaging to protein structure and likely to have deleterious effect on protein function
c.1807C>T	p.P603S	Missense variant, moderate impact and potential modifier of downstream gene regulation. Only possibly damaging to protein structure and likely to have deleterious effect on protein function
c.1555G>A	p.G519R	Missense variant, moderate impact and potential modifier of downstream gene regulation. Probably damaging to protein structure and likely to have deleterious effect on protein function
c.1835G>A	p.G612D	Missense variant, moderate impact and potential modifier of downstream gene regulation. Probably damaging to protein structure and likely to have deleterious effect on protein function
c.1853G > A	p.G618E	Missense variant, moderate impact and potential modifier of downstream gene regulation. Probably damaging to protein structure and likely to have deleterious effect on protein function
c.2494G>A	p.G832R	Missense variant, moderate impact and potential modifier of downstream gene regulation. Probably damaging to protein structure and likely to have deleterious effect on protein function
c.2563G>C	p.G855R	Missense variant, moderate impact and potential modifier of downstream gene regulation. Probably damaging to protein structure and likely to have deleterious effect on protein function
c.2581G>A	p.G861S	Missense variant, moderate impact and potential modifier of downstream gene regulation. Probably damaging to protein structure and likely to have deleterious effect on protein function
c.2599G>A	p.G867R	Missense variant, moderate impact and potential modifier of downstream gene regulation. Probably damaging to protein structure and likely to have deleterious effect on protein function

c.2608G>A	p.G870R	Missense variant, moderate impact and potential modifier of downstream gene regulation. Probably damaging to protein structure and likely to have deleterious effect on protein function
c.2636G>A	p.G879E	Missense variant, moderate impact and potential modifier of downstream gene regulation. Probably damaging to protein structure and likely to have deleterious effect on protein function
c.2645G>A	p.G882D	Missense variant, moderate impact and potential modifier of downstream gene regulation. Probably damaging to protein structure and likely to have deleterious effect on protein function
c.2662G>A	p.G888R	Missense variant, moderate impact and potential modifier of downstream gene regulation. Probably damaging to protein structure and likely to have deleterious effect on protein function
c.2689G>A	p.G897S	Missense variant, moderate impact and potential modifier of downstream gene regulation. Probably damaging to protein structure and likely to have deleterious effect on protein function
c.2699G>A	p.G900E	Missense variant, moderate impact and potential modifier of downstream gene regulation. Probably damaging to protein structure and likely to have deleterious effect on protein function
c.2744G>A	p.G915E	Missense variant, moderate impact and potential modifier of downstream gene regulation. Probably damaging to protein structure and likely to have deleterious effect on protein function
c.2782G>C	p.D928H	Missense variant, moderate impact and potential modifier of downstream gene regulation. Probably damaging to protein structure and likely to have deleterious effect on protein function
c.2842G>A	p.G948S	Missense variant, moderate impact and potential modifier of downstream gene regulation. Probably damaging to protein structure and likely to have deleterious effect on protein function
c.2987G>A	p.G996D	Missense variant, moderate impact and potential modifier of downstream gene regulation. Probably damaging to protein structure and likely to have deleterious effect on protein function
c.3022G>A	p.G1008R	Missense variant, moderate impact and potential modifier of downstream gene regulation. Probably damaging to protein structure and likely to have deleterious effect on protein function
c.3040G>C	p.G1014R	Missense variant, moderate impact and potential modifier of downstream gene regulation. Probably damaging to protein structure and likely to have deleterious effect on protein function
c.3104G>T	p.G1035V	Missense variant, moderate impact and potential modifier of downstream gene regulation. Probably damaging to protein structure and likely to have deleterious effect on protein function
c.3122G>A	p.G1041E	Missense variant, moderate impact and potential modifier of downstream gene regulation. Probably damaging to protein structure and likely to have deleterious effect on protein function

c.3130G>C	p.G1044E	Missense variant, moderate impact and potential modifier of downstream gene regulation. Probably damaging to protein structure and likely to have deleterious effect on protein function
c.3190G>A	p.G1064S	Missense variant, moderate impact and potential modifier of downstream gene regulation. Probably damaging to protein structure and likely to have deleterious effect on protein function
c.191G>T	p.G64V	Missense variant, moderate impact and potential modifier of upstream gene regulation. Probably damaging to protein structure and likely to have deleterious effect on protein function. Possible 3'UTR variant.
c.4739G>C	p.G1580A	Missense variant, moderate impact, deleterious and likely to impact protein function, probably damaging
c.4881C>G	p.N1627K	Missense variant, moderate impact, deleterious and likely to impact protein function, probably damaging
c.4843G>A	p.E1615K	Missense variant, Moderate impact, possibly retained intron, probably damaging
c.4232G>C	p.G1411A	Missense variant, moderate impact. Probably damaging and likely to have deleterious effect on protein function
c.4380T>G	p.C1460W	Missense variant, moderate impact. Probably damaging and likely to have deleterious effect on protein function
c.4652G>A	p. C1551Y	Missense variant, moderate impact. Probably damaging and likely to have deleterious effect on protein function
c.4717G>A	p.G1573R	Missense variant, moderate impact. Probably damaging and likely to have deleterious effect on protein function
c.4738 G > A	p.G1580S	Missense variant, moderate impact. Probably damaging and likely to have deleterious effect on protein function
c.4738G>A	p. G1580S	Missense variant, moderate impact. Probably damaging and likely to have deleterious effect on protein function
c.1955G>A	p. G652E	Missense variant, moderate impact. Probably damaging to protein structure and likely to have deleterious effect on protein function
c.1963G>A	p.G655R	Missense variant, moderate impact. Probably damaging to protein structure and likely to have deleterious effect on protein function

c.1964G>A	p.G655E	Missense variant, moderate impact. Probably damaging to protein structure and likely to have deleterious effect on protein function
c.1973C>A	p. G658V	Missense variant, moderate impact. Probably damaging to protein structure and likely to have deleterious effect on protein function
c.1973G>A	p.G658D	Missense variant, moderate impact. Probably damaging to protein structure and likely to have deleterious effect on protein function
c.2441 G > T	p.G814V	Missense variant, non-coding exon variant with moderate impact. Possibly damaging to protein structure and deleterious to protein function
c.2413G>A	p.G805R	Missense variant, non-coding exon variant with moderate impact. Possibly damaging to protein structure and deleterious to protein function
c.2413G>C	p. G805R	Missense variant, non-coding exon variant with moderate impact. Possibly damaging to protein structure and deleterious to protein function
c.2317G>A	p.G773R	Missense variant, non-coding exon variant with moderate impact. Probably damaging to protein structure and deleterious to protein function
c.2317G>C	p.G773R	Missense variant, non-coding exon variant with moderate impact. Probably damaging to protein structure and deleterious to protein function
c.2228G>T	p.G743V	Missense variant, non-coding exon variant with moderate impact. Regulatory region variant, leading to open chromatin structure. Probably damaging to protein structure and deleterious to protein function
c.2245G>A	p.G749S	Missense variant, non-coding exon variant with moderate impact. Regulatory region variant, leading to open chromatin structure. Probably damaging to protein structure and deleterious to protein function
c.2263G>A	p.G755R	Missense variant, non-coding exon variant with moderate impact. Regulatory region variant, leading to open chromatin structure. Probably damaging to protein structure and deleterious to protein function
c.4267G>C	p.G1423R	Missense variant, possibly resulting in retained intron. Possibly damaging and likely deleterious to protein function
c.4133G>A	p.G1378D	Missense variant, potentially impacting upstream gene regulation. Likely deleterious and probably damaging

c.4150+1(IVS46) G>T		Missense variant, splice donor variant with potential impact on upstream gene regulation. High impact. Probably damaging and likely deleterious.
c.4150+1G>A		Missense variant, splice donor variant with potential impact on upstream gene regulation. High impact. Probably damaging and likely deleterious.
c.4150G>A	p.G1384S	Missense variant, splice donor variant with potential impact on upstream gene regulation. High impact. Probably damaging and likely deleterious.
c.2345G>C	p.G782A	Missense variant, splice region variant with low-moderate impact. Likely deleterious and probably damaging
c.2096G>A	pG699D	Missense variant, splice region variant with low-moderate impact. Likely deleterious to protein function and probably damaging to protein structure
c.236G>T	p.G79V	Missense variant, splice region variant with low-moderate impact. Likely deleterious to protein function and probably damaging to protein structure
c.443G>A	p.G148E	Missense variant, splice region variant with low-moderate impact. Likely deleterious to protein function and probably damaging to protein structure
c.196C>A	p.Q66K	Missense variant. Change tolerated by protein function but possibly damaging to protein structure
c.2641A>G	p.M881V	Missense variant. Change tolerated by protein function but possibly damaging to protein structure
c.3046A>G	p.M1016V	Missense variant. Change tolerated by protein function but possibly damaging to protein structure
c.31C>A	p.L11M	Missense variant. Change tolerated by protein function but possibly damaging to protein structure
c.1612C>G	p.R538G	Missense variant. Change tolerated by protein function with benign impact on protein structure
c.1769G>A	p.G562E	Missense variant. Change tolerated by protein function with benign impact on protein structure
c.3946C>G	p.Q1316E	Missense variant. Change tolerated by protein function, likely benign some evidence of possibly damaging protein structure
c.1537-2A>G		Potential frameshift variant and splice acceptor variant with high impact
c.1537–2delA		Potential frameshift variant and splice acceptor variant with high impact
c.1121-2dupA	p.G374_N429 delinsD	Splice acceptor variant, intronic variant leading to retained intron. High impact variant.
c.1382-1G>C		Splice acceptor variant, intronic variant leading to retained intron. High impact variant.
c.2194-1G.A		Splice acceptor variant, intronic variant leading to retained intron. High impact variant. Also potential regulatory region variant leading to open chromatin structure

c.553-2A>G		Splice acceptor variant, intronic variant leading to retained intron. High impact variant. Also potential regulatory region variant leading to open chromatin structure and altered downstream gene regulation. Potential 3' UTR variant
c.1990+1G>A		Splice donor variant with high impact. Possible retained intron
c.3406 + 1G>T		Splice donor variant with high impact. Potential impact on both upstream and downstream gene regulation
c.2716 + 1G>A		Splice donor variant with high impact. Potential impact on downstream gene regulation
c.2716+ G>T		Splice donor variant with high impact. Potential impact on downstream gene regulation
c.2716+2T>C		Splice donor variant with high impact. Potential impact on downstream gene regulation
c.2458+1G>A		Splice donor variant, high impact. Possibly retained intron and downstream gene regulation modification
c.1A>T		Start lost, but seems to be tolerated by protein function but possibly damaging to protein structure. Possible impact on upstream gene regulation
c.739C>T	p.Q247*	Stop gained, high impact. Possible modifier of downstream gene regulation
c.607G>T	p. G203R	Stop gained, high impact. Potential 3'UTR regulatory variant
c.4875C>A	p.Y1625*	Stop gained, likely deleterious, high impact
c.4887C>A	p.Y1629X	Stop gained, likely deleterious, high impact
c.1870G>T	p.G624*	Stop gained, likely deleterious, high impact. Possible modifier of downstream gene regulation

D. *COL4A2*

**Genetic mutation**	**Protein change**	**Variant information**
c.1396G>A	p.G466S	Missense variant with moderate impact. Probably damaging to protein structure and deleterious to protein function. Possible intron variant causing alteration to lncRNA influencing gene AS2
c.1776+1G>A		Splice donor variant with high impact. Possible retained intron and impact to lncRNA influencing gene AS2. Pathogenic but also reported to have uncertain clinical significance
c.1810G>C	p.G604R	Missense variant, moderate impact and potential modifier of upstream and downstream gene regulation. Probably damaging to protein structure and likely to have deleterious effect on protein function. Potenital influence on promoter refulation and lncRNA influencing AS2

c.1856G>A	p.G619D	Missense variant, moderate impact and potential modifier of upstream and downstream gene regulation. Probably damaging to protein structure and likely to have deleterious effect on protein function. Potenital influence on promoter refulation and lncRNA influencing AS2. Likely pathogenic
c.2105G>A	p.G702D	Missense variant with moderate impact. Probably damaging to protein structure and deleterious to protein function
c.2399G>A	p.G800E	Missense variant with moderate impact. Probably damaging to protein structure and deleterious to protein function. With possible impact on upstream gene regulation and promoter regions
c.2821G>A	p.G941R	Missense variant, non-coding exon variant with moderate impact. Possibly damaging to protein structure and deleterious to protein function. Possible retained intron.
c.3110G>A	p.G1037E	Missense variant, moderate impact and potential modifier of downstream gene regulation. Probably damaging to protein structure and likely to have deleterious effect on protein function. Pathogenic
c.3368A>G	p.E1123G	Missense variant, non-coding exon variant with moderate impact. Possibly damaging to protein structure and deleterious to protein function. Likely benign clinical significance but possible risk factor
c.3448C>A	p.Q1150K	Missense variant, non-coding exon variant with moderate impact. Possibly damaging to protein structure and tolerated to protein function. Likely benign clinical significance but possible risk factor
c.3455G>A	p.G1152D	Missense variant, splice region variant. Probably damaging to protein structure and deleterious to protein function. Pathogenic
c.3490G>A	p.R1164G	Missense variant with moderate impact. Probably damaging to protein structure and deleterious to protein function. Pathogenic
c.4129G > A	p.G1377R	Missense variant, moderate impact and potential modifier of upstream gene regulation, possible impact on lncRNA influencing AS2. Probably damaging to protein structure and likely to have deleterious effect on protein function. Pathogenic
c.4147G>A	p.G1383R	Missense variant, moderate impact and potential modifier of upstream gene regulation, possible impact on lncRNA influencing AS2. Probably damaging to protein structure and likely to have deleterious effect on protein function. Likely pathogenic
c.4987G>A	p.G1663S	Missense variant, moderate impact and potential modifier of both upstream and downstream gene regulation, possible impact on lncRNA influencing AS2. Probably damaging to protein structure and likely to have deleterious effect on protein function. Conflicting clinical significane, reported both likely benign and likely pathogenic
c.5068G>A	p.A1690T	Missense variant, non-coding exon variant with moderate impact. Possibly damaging to protein structure and tolerated to protein function. Likely benign clinical significance but possible risk factor

E. *TREX1*

Genetic mutation	Protein change	Variant information
c.703dup	p.V235GfsX6	Frameshift mutation with high impact. Potentially leading to premature stop. Possible impact on downstream gene regulation of ATRIP and SHISA5 (non-mediated decay)
c.822delT	p.P275Qfsx2	Frameshift mutation with high impact. Potentially leading to premature stop. Possible impact on downstream gene regulation of ATRIP and SHISA5 (non-mediated decay)
c.830-833dupAGGA	p.D278fs	Intronic variant. Potential 3'UTR variant with downstream gene variation. Possible influence on ATRIP and nonsense mediated decay of SHISA5
c.829A>T	p.K277*	Stop gained, high impact. Possible modifier of downstream gene regulation. Likely pathogenic.
c.828_831dupGAAG	p.D278EfsTer48	Frameshift mutation with high impact. Potentially leading to premature stop. Possible impact on downstream gene regulation of ATRIP and SHISA5 (non-mediated decay)
c.703dupG	p.V235Gfs	Frameshift mutation with high impact. Potentially leading to premature stop. Possible impact on downstream gene regulation of ATRIP and SHISA5 (non-mediated decay)
c.685A>G	p.Arg229Gly	Missense variant, moderate impact and potential modifier of downstream gene regulation. Benign impact on protein structure and tolerated by protein function
c.690G>T	p.Lys230Asn	Missense variant, moderate impact and potential modifier of downstream gene regulation. Benign impact on protein structure and could have deleterious effect on protein function, but tolerated also reported
c.581delC	p.Ala194fs	Frameshift variant with high impact, possible downstream gene regulation of ATRIP and SHISA5. Pathogenic
c.742_745dupGTCA	p.T249fs	Intronic variant. Potential 3'UTR variant with downstream gene variation. Possible influence on ATRIP and nonsense mediated decay of SHISA5
c.734dupC	?	Frameshift mutation with high impact. Potentially leading to premature stop. Possible impact on downstream gene regulation of ATRIP and SHISA5 (non-mediated decay)
c.911_912delCA	p.T304Nfs*12	Intronic variant. Potential 3'UTR variant with downstream gene variation. Possible influence on ATRIP and nonsense mediated decay of SHISA5
c.703_704insG	p.V235GfsX6	Frameshift mutation with high impact. Potentially leading to premature stop. Possible impact on downstream gene regulation of ATRIP and SHISA5 (non-mediated decay)

Supplemental References:

1. Abe Y, Matsuduka A, Okanari K, Miyahara H, Kato M, Miyatake S, Saitsu H, Matsumoto N, Tomoki M, Ihara K. A severe pulmonary complication in a patient with COL4A1-related disorder: A case report. Eur J Med Genet. 2017;60:169-171.
2. Adams KL, Riparini G, Banerjee P, Breur M, Bugiani M, Gallo V. Endothelin-1 signaling maintains glial progenitor proliferation in the postnatal subventricular zone. Nat. Commun. 2020;11:2138.
3. Agharahimi A, Bergerson J, Sun A, Similuk M, Oler A, Mace E, Stone D, Ombrello A, Freeman A. Warts as a predominant manifestation of ADA2 deficiency. J Clin Immunol. 2018;38:417. (Abstract)
4. Akgun-Dogan O, Simsek-Kiper PO, Taskiran E, Lissewski C, Brinkmann J, Schanze D, Gocmen R, Cagdas D, Bilginer Y, Utine GE, et al. ADA2 deficiency in a patient with Noonan syndrome-like disorder with loose anagen hair: The co-occurrence of two rare syndromes. Am. J. Med. Genet. A. 2019;179:2474–2480.
5. Al Mosawi Z, Abduljawad H, Busehail M, Al Moosawi B. Adenosine deaminase 2 deficiency with a novel variant of CECR1 gene mutation: Responding to tumor necrosis factor antagonist therapy. Indian J. Rheumatol. 2019;14:236–240.
6. Alabbas F, Elyamany G, Alsharif O, Hershfield M, Meyts I. Childhood Hodgkin Lymphoma: Think DADA2. J Clin Immunol. 2019;39:26-29.
7. Alamowitch S, Plaisier E, Favrole P, Prost C, Chen Z, Van Agtmael T, Marro B, Ronco P. Cerebrovascular disease related to COL4A1 mutations in HANAC syndrome. Neurology. 2009;73:1873-1882.
8. Alao H, Kleiner D, Han MAT, Takyar V, Stone D, Hoffmann P, Ombrello A, Jones A, Kastner D, Heller T. Deficiency of adenosine deaminase 2 (DADA2); a rare cause of hepatoportal sclerosis and non-cirrhotic portal hypertension. Gastroenterology. 2016;150:S1172-1173. (Abstract)
9. Alaygut D, Alparslan C, Perihan Oncel E, Mutlubas F, Ozdemir T, Yavascan O, Kasap Demir B. A child diagnosed with treatment-resistant polyarteritis nodosa: Can the clinical diagnosis be different? Arch. Rheumatol. 2019;34:338–342.
10. Alsultan A, Basher E, Alqanatish J, Mohammed R, Alfadhel M. Deficiency of ADA2 mimicking autoimmune lymphoproliferative syndrome in the absence of livedo reticularis and vasculitis. Pediatr Blood Cancer. 2018;65.
11. Araujo JM, Alves JN, Taipa R, Alonso I, Ferreira C, Pinho J. Small vessel disease and intracerebral hemorrhages associated with novel pathogenic variants of col4a1. Europ Stroke J. 2018;3:582-583.
12. Arts K, Bergerson JRE, Ombrello AK, Similuk M, Oler AJ, Agharahimi A, Mace EM, Hershfield M, Wouters C, De Somer L et al. Warts and DADA2: a Mere Coincidence? J Clin Immunol. 2018;38:836-843.
13. Ayrignac X, Carra-Dalliere C, Menjot de Champfleur N, Denier C, Aubourg P, Bellesme C, Castelnovo G, Pelletier J, Audoin B, Kaphan E et al. Adult-onset genetic leukoencephalopathies: a MRI pattern-based approach in a comprehensive study of 154 patients. Brain. 2015;138:284-292.
14. Bademkiran F, Nalcaci S, Eraslan C, Durmaz A. The first Turkish family with the diagnosis of retinal vasculopathy with cerebral leukodystrophy (RVCL) where a new mutation was found. J Neurol Sci. 2017;381:378-379.

15. Balakrishna JP, Hsu A, Ombrello A, Wang W, Holland SM, Hickstein DD, Kastner DL, Aksentijevich I, Calvo KR. Spectrum of bone marrow pathology in patients with germline mutations in CECR1. Lab Inv. 2017;97:338A. (Abstract)
16. Barron K, Ombrello A, Stone D, Hoffmann P, Aksentijevich I, Zhou Q, Jones A, Kastner D. Clinical follow-up on a cohort of patients with deficiency of adenosine deaminase 2 (DADA2). Pediatric Rheumatology. 2015;13:19. (Abstract)
17. Barron K, Ombrello A, Stone D, Hoffmann P, Aksentijevich I, Zhou Q, Jones A, Kastner D. Deficiency of adenosine deaminase type ii-expanding the clinical spectrum. Arthritis Rheumatol. 2015;67. (Abstract)
18. Barron K, Ombrello A, Stone D, Hoffmann P, Romeo T, Jones A, Moura NS, Schnappauf O, Aksentijevich I, Bergerson J et al. The clinical spectrum of the deficiency of adenosine deaminase 2 (DADA2) continues to expand. Pediatr Rheumatol. 2019;17:1. (Abstract)
19. Barzaghi F, Minniti F, Mauro M, Bortoli M, Balter R, Bonetti E, Zaccaron A, Vitale V, Omrani M, Zoccolillo M et al. ALPS-Like Phenotype Caused by ADA2 Deficiency Rescued by Allogeneic Hematopoietic Stem Cell Transplantation. Front Immunol. 2018;9:2767.
20. Batu ED, Karadag O, Taskiran EZ, Kalyoncu U, Aksentijevich I, Alikasifoglu M, Ozen S. A case series of adenosine deaminase 2 deficient patients emphasizing treatment and genotype-phenotype correlations. Pediatr Rheumatol. 2015;13:P62. (Abstract)
21. Batu ED, Karadag O, Taskiran EZ, Kalyoncu U, Aksentijevich I, Alikasifoglu M, Özen S. A Case Series of Adenosine Deaminase 2-deficient Patients Emphasizing Treatment and Genotype-phenotype Correlations. J Rheumatol. 2015;42:1532-1534.
22. Batu ED, Karadag O, Taskiran EZ, Kalyoncu U, Aksentijevich I, Ozen S. A case series of adenosine deaminase 2 deficient patients emphasizing genotype-phenotype correlations. Ann Rheumat Dis. 2015;74:518. (Abstract)
23. Batu ED, Sonmez HE, Erden A, Taskiran EZ, Karadag O, Kalyoncu U, Oncel I, Kaplan B, Arici ZS, Temucin CM et al. The characteristic features of the patients with deficiency of adenosine deaminase 2 (DADA2). Pediatr Rheumatol. 2017;15:P403. (Abstract)
24. Batu ED, Taskiran EZ, Ozkara HA, Unal S, Guleray N, Erden A, Karadag O, Gumruk F, Cetin M, Bilginer Y, et al. A monogenic disease with wide range of symptoms: Deficiency of adenosine deaminase 2. Ann. Rheum. Dis. 2019;78:1748. (Abstract)
25. Bayrakli F, Balaban H, Gurelik M, Hizmetli S, Topaktas S. Mutation in the HTRA1 gene in a patient with degenerated spine as a component of CARASIL syndrome. Turk Neurosurg. 2014;24:67-69.
26. Baytaroglu A, Kadayifcilar S, Agin A, Delktas O, Demr S, Bigner Y, Karakaya J, Ozen S, Eldem B. Choroidal vascularity index as a biomarker of systemic inflammation in childhood Polyarteritis Nodosa and adenosine deaminase-2 deficiency. Pediatr. Rheumatol. 2020;18:29.
27. Beaufort N, Scharrer E, Kremmer E, Lux V, Ehrmann M, Huber R, Houlden H, Werring D, Haffner C, Dichgans M. Cerebral small vessel disease-related protease HtrA1 processes latent TGF-beta binding protein 1 and facilitates TGF-beta signaling. Proc Natl Acad Sci USA. 2014;111:16496-16501.
28. Belot A, Wassmer E, Twilt M, Lega JC, Zeef LA, Oojageer A, Kasher PR, Mathieu AL, Malcus C, Demaret J et al. Mutations in CECR1 associated with a neutrophil signature in peripheral blood. Pediatr Rheumatol Online J. 2014;12:44.

29. Ben-Ami T, Revel-Vilk S, Brooks R, Shaag A, Hershfield MS, Kelly SJ, Ganson NJ, Kfir-Erenfeld S, Weintraub M, Elpeleg O et al. Extending the Clinical Phenotype of Adenosine Deaminase 2 Deficiency. J Pediatr. 2016;177:316-320.
30. Bertamino M, Grossi A, Severino M, Tortora D, Signa S, Amico G, Di Rocco M, Ceccherini I. Next generation sequencing based gene panel for enhanced rapid diagnosis of monogenic pediatric stroke. Eur. Stroke J. 2019;4:355. (Abstract)
31. Bianchi S, Di Palma C, Gallus GN, Gallus GF, Taglia I, Poggiani A, Rosini F, Rufa A, Muresanu DF, Cerase A et al. Two novel HTRA1 mutations in a European CARASIL patient et al. Neurology 2014, 82 (10), 898-900.
32. Bianchi S, Di Palma C, Gallus GN, Taglia I, Poggiani A, Rosini F, Cerase A, Rufa A, Muresanu D, Dotti MT et al. Journal of the Neurological Sciences. 2013;333:e660. (Abstract)
33. Bick D, Fraser PC, Gutzeit MF, Harris JM, Hambuch TM, Helbling DC, Jacob HJ, Kersten JN, Leuthner SR, May T et al. Successful Application of Whole Genome Sequencing in a Medical Genetics Clinic. J Pediatr Genet. 2017;6:61-76.
34. "Bilguvar K, DiLuna ML, Bizzarro MJ, Bayri Y, Schneider KC, Lifton RP, Gunel M, Ment LR. COL4A1 mutation in preterm intraventricular hemorrhage. J Pediatr. 2009;155:743-745."
35. Bougea A, Velonakis G, Spantideas N, Anagnostou E, Paraskevas G Kapaki E, Kararizou E. The first Greek case of heterozygous cerebral autosomal recessive arteriopathy with subcortical infarcts and leukoencephalopathy: An atypical clinico-radiological presentation. Neuroradiol J. 2017;30:583-585.
36. Breedveld G, de Coo IF, Lequin MH, Arts WF, Heutink P, Gould DB, John SW, Oostra B, Mancini GM. Novel mutations in three families confirm a major role of COL4A1 in hereditary porencephaly. J Med Genet. 2006;43:490-495.
37. Bucciol G, Delafontaine S, Segers H, Bossuyt X, Hershfield MS, Moens L, Meyts I. Hematopoietic Stem Cell Transplantation in ADA2 Deficiency: Early Restoration of ADA2 Enzyme Activity and Disease Relapse upon Drop of Donor Chimerism. J Clin Immunol. 2017;37:746-750.
38. Bugiani M, Bakels HS, Waisfisz Q, Ceuterick-de Groote C, Niessen HW, Abbink TE, Lesnik Oberstein SA, van der Knaap MS. Cathepsin A-related arteriopathy with strokes and leukoencephalopathy (CARASAL). Neurology. 2016;87:1777-1786.
39. Bulut E, Erden A, Karadag O, Oguz KK, Ozen S. Deficiency of adenosine deaminase 2; special focus on central nervous system imaging. J Neuroradiol. 2019;46:193-198.
40. Caetano A, Barbosa R, Costa J, Viana-Baptista M. Sindrome HANAC (Hereditary angiopathy, nephropathy, aneurysms, and muscle cramps) incompleto e 1a mutacao descrita do gene C0L4A1 em Portugal (G236T). Sinapse. 2015;15:23-26.
41. Cai B, Zeng J, Lin Y, Lin W, Li Z, Wang N. A frameshift mutation in HTRA1 expands CARASIL syndrome and peripheral small arterial disease to the Chinese population. Neurol Sci. 2015;36:1387-1391.
42. Cakan M, Aktay-Ayaz N, Karadag SG, Tahir-Turanli E, Stafstrom K, Bainter W, Geha RS, Chou J. Atypical phenotype of an old disease or typical phenotype of a new disease: Deficiency of adenosine deaminase 2. Turk. J. Pediatr. 2019;61:413–417.
43. Campo-Caballero D, Rodriguez-Antiguedad J, Ekiza-Bazan J, Iruzubieta-Agudo P, Fernandez-Eulate G., Munoz-Lopetegui A, Martinez-Zabaleta M, de la Riva P, Urtasun-Ocariz M, de Munain AL, et al. COL4A1 Mutation as a Cause of Familial Recurrent Intracerebral Hemorrhage. J. Stroke Cerebrovasc. Dis. 2020;29:104652.

44. Caorsi R, Grossi A, Cusano R, Rusmini M, Penco F, Schena F, Anna Podda R, Uva P, Gattorno M, Ceccherini I. ADA2 deficiency without ADA2 mutations explained by a structural homozygous variation in 22q11.1. Pediatr Rheumatol. 2018;16. (Abstract)
45. Caorsi R, Grossi A, Insalaco A, Alessio M, Martino S, Cortis E, Morreale A, Caroli F, Martini A, Ceccherini I et al. Prevalence of cecr1 mutations in pediatric patients with polyarteritis nodosa, livedo reticularis and/or stroke. Ann Rheumat Dis. 2015;74:835. (Abstract)
46. Caorsi R, Grossi A, Insalaco A, Alessio M, Martino S, Cortis E, Morreale A, Caroli F, Martini A, Ceccherini I et al. Prevalence of CECR1 mutations in pediatric patients with polyarteritis nodosa, livedo reticularis and/or stroke. Pediatr Rheumatol. 2015;13:O87. (Abstract)
47. Caorsi R, Omenetti A, Morreale A, Insalaco A, Buoncompagni A, Picco P, Malattia C, Gandolfo C, Aksentijevich I, Martini A et al. Rapid and sustained effect of anti-TNF treatment in patients with ADA2 deficiency. Pediatr Rheumatol. 2016;13:74. (Abstract)
48. Caorsi R, Omenetti A, Picco P, Buoncompagni A, Minoia F, Federici S, Finetti M, Martini A, Aksentijevich I, Gattorno M. Long-term efficacy of etanercept in ADA2 deficiency. Pediatr Rheumatol. 2014;12. (Abstract)
49. Caorsi R, Penco F, Grossi A, Insalaco A, Omenetti A, Alessio M, Conti G, Marchetti F, Picco P, Tommasini A, et al. ADA2 deficiency (DADA2) as an unrecognised cause of early onset polyarteritis nodosa and stroke: A multicentre national study. Ann. Rheum. Dis. 2017;76:1648–1656.
50. Caorsi R, Severino MS, Gandolfo C, Ravelli A, Rossi A, Gattorno M. Distinct cerebrovascular features in patients with ADA2 deficiency. Pediatr Rheumatol. 2018;16. (Abstract)
51. Carneiro D, Fernandes C, Santo GC. Familial sneddon's syndrome revisited: The natural history of deficiency of adenosine deaminase 2. Eur. Stroke J. 2019;4:355–356. (Abstract)
52. Carra-Dalliere C, Ayrignac X, Prieto-Morin C, Girard P, Tournier-Lasserve E, Labauge P. TREX1 Mutation in Leukodystrophy with Calcifications and Persistent Gadolinium-Enhancement. Eur Neurol. 2017;77:113-114.
53. Cavallin M, Mine M, Philbert M, Boddaert N, Lepage JM, Coste T, Lopez-Gonzalez V, Sanchez-Soler MJ, Ballesta-Martínez MJ, Remerand G et al. Further refinement of COL4A1 and COL4A2 related cortical malformations. Eur J Med Genet. 2018;61:765-772.
54. Chang Y, Derfalvi B, Issekutz A, Shi J, Alonzo P, Pascual CJ, Issekutz T, Walter JE. ADA2 deficiency: Case report of a rare phenotype with alps and CVID-like presentation. J Clin Immunol. 2018;38:341-342.
55. Chen Y, He Z, Meng S, Li L, Yang H, Zhang X. A novel mutation of the high-temperature requirement A serine peptidase 1 (HTRA1) gene in a Chinese family with cerebral autosomal recessive arteriopathy with subcortical infarcts and leukoencephalopathy (CARASIL). J Int Med Res. 2013;41:1445-1455.
56. Chong-Neto HJ, Segundo GRS, Bandeira M, Riedi CA, Hershfield M, Torgerson TR, Rosario N. Novel CECR1 gene mutation causing ADA-2 deficiency. J Clin Immunol. 2018;38:387. (Abstract)
57. Christ S, Haber B, Grulich-Henn J, Helling-Bakki A, Hoffmann GF, Lutz T, Tonshoff B, Syrbe S, Haas D, Youssef H et al. Unclear strokes in pediatrics-adenosine deaminase 2

(ADA2) deficiency as a therapeutic relevant differential diagnosis to acquired inflammatory cns diseases. Neuropediatrics. 2018;49. (Abstract)
58. Cimino M, Soo S, Morrow M. Ring-enhancing lesions, stroke and vascular retinopathy associated with a novel TREX1 mutation. Neurology. 2018;90:15. (Abstract)
59. Cipe FE, Aydogmus C, Serwas NK, Keskindemirci G, Boztuğ K. Novel Mutation in CECR1 Leads to Deficiency of ADA2 with Associated Neutropenia. J Clin Immunol. 2018;38:273-277.
60. Clarke K, Campbell C, Omoyinmi E, Hong Y, Obaidi MAL, Sebire N, Brogan P. Testicular ischemia in deficiency of adenosine deaminase 2 (DADA2). Pediatr Rheumatol. 2019;17. (Abstract)
61. Cohn AC, Kotschet K, Veitch A, Delatycki MB, McCombe MF. Novel ophthalmological features in hereditary endotheliopathy with retinopathy, nephropathy and stroke syndrome. Clin Exp Ophthalmol. 2005;33:181-183.
62. Colin E, Sentilhes L, Sarfati A, Mine M, Guichet A, Ploton C, Boussion F, Delorme B, Tournier-Lasserve E, Bonneau D. Fetal intracerebral hemorrhage and cataract: think COL4A1. J Perinatol. 2014;34:75-77.
63. Corlobe A, Tournier-Lasserve E, Mine M, Menjot de Champfleur N, Carra Dalliere C, Ayrignac X, Labauge P, Arquizan C. COL4A1 mutation revealed by an isolated brain hemorrhage. Cerebrovasc Dis. 2013;35:593-594.
64. Cornec-Le Gall E, Chebib FT, Madsen CD, Senum SR, Heyer CM, Lanpher BC, Patterson MC, Albright RC, Yu AS, Torres VE et al. The Value of Genetic Testing in Polycystic Kidney Diseases Illustrated by a Family With PKD2 and COL4A1 Mutations. Am J Kidney Dis. 2018;72:302-308.
65. Cornec-Le Gall E, Heyer C, Senum S, Audrezet M-P, Le Meur Y, Torres V, Harris P. Identifying the culprit gene in 400 genetically unresolved autosomal dominant polycystic kidney or liver disease (ADPKD/ADPLD) pedigrees. Nephrol Dial Transplant. 2017;32:3. (Abstract)
66. Coupry I, Sibon I, Mortemousque B, Rouanet F, Mine M, Goizet C. Ophthalmological features associated with COL4A1 mutations. Arch Ophthalmol. 2010;128:483-489.
67. Coutts SB, Matysiak-Scholze U, Kohlhase J, Innes AM. Intracerebral hemorrhage in a young man. CMAJ. 2011:183:E61-E64.
68. Craggs LJ, Hagel C, Kuhlenbaeumer G, Borjesson-Hanson A, Andersen O, Viitanen M, Kalimo H, McLean CA, Slade JY, Hall RA et al. Quantitative vascular pathology and phenotyping familial and sporadic cerebral small vessel diseases. Brain Pathol. 2013;23:547-557.
69. Dahl S, Pettersson M, Eisfeldt J, Schroder AK, Wickstrom R, Tear Fahnehjelm K, Anderlid BM, Lindstrand A. Whole genome sequencing unveils genetic heterogeneity in optic nerve hypoplasia. PloS One. 2020;15:e0228622.
70. De Vries LS, Koopman C, Groenendaal F, Van Schooneveld M, Verheijen FW, Verbeek E, Witkamp TD, van der Worp HB, Mancini G. COL4A1 mutation in two preterm siblings with antenatal onset of parenchymal hemorrhage. Ann Neurol. 2009;65:12-18.
71. Decio A, Tonduti D, Pichiecchio A, Vetro A, Ciccone R, Limongelli I, Giorda R, Caffi L, Balottin U, Zuffardi O et al. A novel mutation in COL4A1 gene: a possible cause of early postnatal cerebrovascular events. Am J Med Genet. 2015;167A:810-815.

72. Değerliyurt A, Ceylaner G, Koçak H, Bilginer Gürbüz B, Cihan BS, Rizzu P, Ceylaner S. A new family with autosomal dominant porencephaly with a novel Col4A1 mutation. Are arachnoid cysts related to Col4A1 mutations? Genet Couns. 2012;23:185- 193.
73. Deml B, Reis LM, Maheshwari M, Griffis C, Bick D, Semina EV. Whole exome analysis identifies dominant COL4A1 mutations in patients with complex ocular phenotypes involving microphthalmia. Clin Genet. 2014;86:475-481.
74. Dhamija R, Schiff D, Lopes MB, Jen JC, Lin DD, Worrall BB. Evolution of brain lesions in a patient with TREX1 cerebroretinal vasculopathy. Neurology. 2015;85:1633- 1634.
75. Di Donato I, Bianchi S, Gallus GN, Cerase A, Federico A, Dotti MT. Heterozygous mutation of HTRA1 gene in an Italian family with cerebral ischemic small vessel disease. Eur J Neurol. 2016;23:559. (Abstract)
76. Di Donato I, Bianchi S, Gallus GN, Cerase A, Taglia I, Pescini F, Nannucci S, Battisti C, Inzitari D et al. Heterozygous mutations of HTRA1 gene in patients with familial cerebral small vessel disease. CNS Neurosci Ther. 2017;23:759-765.
77. DiFrancesco JC, Novara F, Zuffardi O, Forlino A, Gioia R, Cossu F, Bolognesi M, Andreoni S, Saracchi E, Frigeni B et al. TREX1 C-terminal frameshift mutations in the systemic variant of retinal vasculopathy with cerebral leukodystrophy. Neurol Sci. 2015;36:323-330.
78. Dimachkie MD, Fraga GR, Moura NS, Springer JM. A Rare Case of Adenosine Deaminase 2 Deficiency Presenting With Temporal Arteritis. J. Clin. Rheumatol. Pract. Rep. Rheum. Musculoskelet. Dis. 2020;00:Publish Ahead of Print.
79. Durrani-Kolarik S, Manickam K, Chen B. COL4A1 Mutation in a Neonate With Intrauterine Stroke and Anterior Segment Dysgenesis. Pediatr Neurol. 2017;66:100-103.
80. Ekinci RMK, Balci S, Bisgin A, Sasmaz I, Leblebisatan G, Incecik F, Yilmaz M. A homozygote novel L451W mutation in CECR1 gene causes deficiency of adenosine deaminase 2 in a pediatric patient representing with chronic lymphoproliferation and cytopenia. Pediatr. Hematol. Oncol. 2019;36:376–381.
81. Ekinci RMK, Balci S, Hershfield M, Bisgin A, Dogruel D, Altintas DU, Yilmaz M. Deficiency of adenosine deaminase 2: A case series revealing clinical manifestations, genotypes and treatment outcomes from Turkey. Rheumatol. U. K. 2020;59:254–256.
82. El Hasbani G, Balaghi A, Assaker R, Rojas A, Troya M, Kofahi A, Assaker JP, Diab C, Al Husayni H. Intraparenchymal hemorrhage and cerebral venous thrombosis in an adult with congenital porencephalic cyst presenting for generalized tonic-clonic seizures. Radiol. Case Rep. 2020;15:95–99.
83. Elbracht M, Mull M, Wagner N, Kuhl C, Abicht A, Kurth I, Tenbrock K, Häusler M. Stroke as Initial Manifestation of Adenosine Deaminase 2 Deficiency. Neuropediatrics. 2017;48:111-114.
84. Erden A, Batu ED, Taskiran EZ, Sonmez HE, Sari A, Armagan B, Kilic L, Arici ZS, Bilginer Y, Akdogan A et al. The characteristic features of the patients with deficiency of adenosine deaminase 2 (DADA2). Arthritis Rheumatol. 2016;68:4112-4114. (Abstract)
85. Ersoy G, Bayram C, Gokce M, Unal S, Ozdemir NN. Ada 2 enzyme deficiency manifesting as pure red cell aplasia. HemaSphere. 2018;2:835. (Abstract)
86. F. Variable clinical phenotypes and relation of interferon signature with disease activity in ADA 2 deficiency. Pediatr Rheumatol. 2017;15. (Abstract)

87. Fasano A, Formichi P, Taglia I, Bianchi S, Di Donato I, Battisti C, Federico A, Dotti MT. HTRA1 expression profile and activity on TGF-β signaling in HTRA1 mutation carriers. J. Cell. Physiol. 2020;235:7120–7127.
88. Favaretto S, Margoni M, Salviati L, Pianese L, Manara R, Baracchini C. A new Italian family with HTRA1 mutation associated with autosomal-dominant variant of CARASIL: Are we pointing towards a disease spectrum? J Neurol Sci. 2019;396:108-111.
89. Fujita M, Shimoyama K, Otsuka N, Maeda Y, Hayashi K, Saitsu H, Matsumoto N, Takanashi J-I. Familiar patients with congenital hemiplegia due to a COL4A1 mutation. No To Hattatsu. 2018;50:424-426.
90. Gale D, Oygar DD, Lin F, Oygar DP, Connor TMF, Khan N, Lapsley M, Maxwell PH, Neild GH. A novel COL4A1 frameshift mutation and kidney disease without extra-renal involvement in a large Turkish cypriot family. Nephrol Dial Transplant. 2015;30:iii385. (Abstract)
91. Gale D, Oygar DD, Lin F, Oygar DP, Khan N, Connor TM, Lapsley M, Maxwell PH, Neild GH. A novel COL4A1 frameshift mutation in familial kidney disease: the importance of the C-terminal NC1 domain of type IV collagen. Nephrol Dial Transplant. 2016;31:1908- 1914.
92. Garbarino F, Caorsi R, Volpi S, Grossi A, Ceccherini I, Gattorno M. A case of adenosine deaminase 2 deficiency (DADA2) with an uncommon clinical presentation and response to IVIG. Pediatr Rheumatol. 2019;17. (Abstract)
93. Garel C, Rosenblatt J, Moutard ML, Heron D, Gelot A, Gonzales M, Miné E, Jouannic JM. Fetal intracerebral hemorrhage and COL4A1 mutation: promise and uncertainty. Ultrasound Obstet Gynecol. 2013;41:228-230.
94. Garg N, Kasapcopur O, Foster J, Barut K, Tekin A, Kızılkılıç O, Tekin M. Novel adenosine deaminase 2 mutations in a child with a fatal vasculopathy. Eur J Pediatr. 2014;173:827-830.
95. Gasparini S, Qualtieri A, Ferlazzo E, Cianci V, Patitucci A, Spadafora P, Aguglia U. Normal immunofluorescence pattern of skin basement membranes in a family with porencephaly due to COL4A1 G749S mutation. Neurol Sci. 2016;37:459-463.
96. Geis T, Schirmer S, Walter M, Rodl T, Albrecht B, Schara U, Hehr U, Kolbel H. Massive parallel sequencing with a multigene panel (MGPS): Experiences with alpha-dystroglycanopathies. Neuropediatrics. 2016;47. (Abstract)
97. Gerasimenko A, Heron D, Billette De Villemeur T, Rodriguez D, Garel C, Tournier-Lasserve E, Chalard F, Mine M, Coste T, Mignot C. TORCH-like encephalopathy due to de novo COL4A1 mutation. Eur. J. Hum. Genet. 2019;27:1428–1429. (Abstract)
98. Ghurye RR, Sundaram K, Smith F, Clark B, Simpson MA, Fairbanks L, Adhya Z, Mufti GJ, Marsh JCW, Ibrahim MAA. Novel ADA2 mutation presenting with neutropenia, lymphopenia and bone marrow failure in patients with deficiency in adenosine deaminase 2 (DADA2). Br J Haematol. 2019;186:e60-64.
99. Gibson K, Cabral D, Drogemoller B, Xhan X, Miao F, Morishita K, Gill E, Hancock REW, Ross C, Brown K. Characterization of adenosine deaminase 2 variants identified in an international pediatric vasculitis cohort. Arthritis Rheumatol. 2017;69:Supplement 10. (Abstract)
100. Gibson KM, Morishita KA, Dancey P, Moorehead P, Drögemöller B, Han X, Graham J, Hancock REW, Foell D, Benseler S et al. Identification of Novel Adenosine Deaminase

2 Gene Variants and Varied Clinical Phenotype in Pediatric Vasculitis. Arthritis Rheumatol. 2019;71:1747-1755.
101. Giorgio E, Vaula G, Bosco G, Giacone S, Mancini C, Calcia A, Cavalieri S, Di Gregorio E, Rigault De Longrais R, Leombruni S et al. Two families with novel missense mutations in COL4A1: When diagnosis can be missed. Neurol Sci. 2015;352:99-104.
102. Gomes I, Galego O, Santo GAPRC, Nunes C. CARASIL: An underdiagnosed disease. Eur. J. Neurol. 2019;26:381. (Abstract)
103. Goncalves T da S, Alves CAPF, da Paz JA, Lucato LT. Teaching NeuroImages: Lacunar stroke and polyarteritis nodosa: Consider ADA2 deficiency (DADA2). Neurology. 2019;92:e1801–e1802.
104. Gonzalez Santiago TM, Zavialov A, Saarela J, Seppanen M, Reed AM, Abraham RS, Gibson LE. Dermatologic Features of ADA2 Deficiency in Cutaneous Polyarteritis Nodosa. JAMA Dermatol. 2015;151:1230-1234.
105. Goschl L, Winkler S, Dmytrus J, Heredia RJ, Lagler H, Ramharter M, Scheinecker C, Bonelli M, Schmetterer K, Pickl WF, et al. Unreported Missense Mutation in the Dimerization Domain of ADA2 Leads to ADA2 Deficiency Associated with Severe Oral Ulcers and Neutropenia in a Female Somalian Patient-Addendum to the Genotype-Phenotype Puzzle. J. Clin. Immunol. 2020;40:223–226.
106. Gould DB, Phalan FC, Breedveld GJ, van Mil SE, Smith RS, Schimenti JC, Aguglia U, van der Knaap MS, Heutink P, John SW. Mutations in Col4a1 cause perinatal cerebral hemorrhage and porencephaly. Science. 2005;308:1167-1171.
107. Gould DB, Phalan FC, van Mil SE, Sundberg JP, Vahedi K, Massin P, Bousser MG, Heutink P, Miner JH, Tournier-Lasserve E et al. NEJM. 2006;354:1489-1496.
108. Green LMC, Berry I, McCullagh HG. Cecr1 mutation is an important cause of brainstem stroke. Dev Med Child Neurol. 2017;59:82-83.
109. Grego L, Pignatto S, Rassu N, Passone E, Cogo P, Lanzetta P. Optic Nerve Hypoplasia, Corpus Callosum Agenesis, Cataract, and Lissencephaly in a Neonate with a Novel COL4A1 Mutation. Case Rep. Ophthalmol. 2019;10:424–430.
110. Grond-Ginsbach C, Brandt T, Kloss M, Aksay SS, Lyrer P, Traenka C, Erhart P, Martin JJ, Altintas A, Siva A et al. Next generation sequencing analysis of patients with familial cervical artery dissection. Eur Stroke J. 2017;2:137-143.
111. Grossi A, Cusano R, Rusmini M, Penco F, Schena F, Podda RA, Caorsi R, Gattorno M, Uva P, Ceccherini I. ADA2 deficiency due to a novel structural variation in 22q11.1. Clinical Genetics. 2019;95:732-733.
112. Grossi A, Garbarino F, Caorsi R, Cusano R, Rusmini M, Penco F, Schena F, Podda RA, Uva P, Ceccherini I et al. ADA2 deficiency without ADA2 mutations explained by a structural homozygous variation in 22Q11.1. Pediatr Rheumatol. 2019;17. (Abstract)
113. Gruver AM, Schoenfield L, Coleman JF, Hajj-Ali R, Rodriguez ER, Tan CD. Novel ophthalmic pathology in an autopsy case of autosomal dominant retinal vasculopathy with cerebral leukodystrophy. J Neuroophthalmol. 2011;31:20-24.
114. Gu J, Bennetts B, Holman K, Wong K, Parratt J, Krishnan A, Tchan M. Cerebral autosomal recessive arteriopathy with subcortical infarcts and leukoencephalopathy (CARASIL): The first Australian cases. Twin Research and Human Genetics. 2016;19:567. (Abstract)
115. Gulati A, Bale AE, Dykas DJ, Bia MJ, Danovitch GM, Moeckel GW, Somlo S, Dahl NK. TREX1 Mutation Causing Autosomal Dominant Thrombotic Microangiopathy and CKD-A Novel Presentation. Am J Kidney Dis. 2018;72:895-899.

116. Gümrük F, Soltanova G, Akarsu N, Cetin M, Unal S. Immunological status of patients with diamond blackfan anemia. Am J Hum Genet. 2018;2:832-833. (Abstract)
117. Gunda B, Kovács T, Hornyák C, Bereczki D, Várallyay G, Rudas G, Mine M, Tournier-Lasserve E. Recurrent intracerebral hemorrhage in a young adult caused by COL4A2 mutation. Int J Stroke. 2015;10:369. (Abstract)
118. Gunda B, Mine M, Kovács T, Hornyák C, Bereczki D, Várallyay G, Rudas G, Audrezet MP, Tournier-Lasserve E. COL4A2 mutation causing adult onset recurrent intracerebral hemorrhage and leukoencephalopathy. J Neurol. 2014;261:500-503.
119. Gunda B, Mine M, Kovács T, Hornyák C, Bereczki D, Várallyay G, Rudas G, Tournier-Lasserve E. Col4a2 mutation causing recurrent intracerebral hemorrhage - Importance of screening both Col4a1 and Col4a2 in ICH of unknown origin. J Neurol Sci 2013;333:e174-175. (Abstract)
120. Gunduz T, Demirkol Y, Dogan O, Demir S, Akcakaya NH. A Case of Leukoencephalopathy and Small Vessels Disease Caused by a Novel HTRA1 Homozygous Mutation. J. Stroke. 2019;28:104354.
121. Ha TT, Sadleir LG, Mandelstam SA, Paterson SJ, Scheffer IE, Gecz J, Corbett MA. A mutation in COL4A2 causes autosomal dominant porencephaly with cataracts. Am J Med Genet. 2016;170A:1059-1063.
122. Ha TT, Sadleir LG, Mandelstam SA, Paterson SJ, Scheffer IE, Gecz J, Corbett MA. A mutation in COL4A2 causes autosomal dominant porencephaly with cataracts. Am J Med Genet. 2016;170A:1059-1063.
123. Hanson-Kahn A, Vlessis K, Smith EJ, Manning M. Defining the clinical features associated with variants in COL4A2: Case report and review of the literature. Eur. J. Hum. Genet. 2019;27:1274.
124. Hara K, Shiga A, Fukutake T, Nozaki H, Miyashita A, Yokoseki A, Kawata H, Koyama A, Arima K, Takahashi T et al. Association of HTRA1 mutations and familial ischemic cerebral small-vessel disease. N Engl J Med. 2009;360:1729-1739.
125. Harada T, Uegaki T, Arata K, Tsunetou T, Taniguchi F. Schizencephaly and Porencephaly Due to Fetal Intracranial Hemorrhage: A Report of Two Cases. Yonago Acta Med. 2017;60:241-245.
126. Hardy TA, Young S, Sy JS, Colley AF, Terwindt GM, Ferrari MD, Hayes MW, Hodkinson S. Tumefactive lesions in retinal vasculopathy with cerebral leucoencephalopathy and systemic manifestations (RVCL-S): a role for neuroinflammation? J Neurol Neurosurg Psychiatry. 2017:316142
127. Harel A, Raynowska J, Miskin D, Pramanik B, Asiry S, Anderson T, Boockvar J, Najjar S. Retinal vasculopathy with cerebral leukoencephalopathy (RVCL): A rare familial mimic of tumefactive multiple sclerosis (MS). Neurology. 2018;90:15. (Abstract)
128. Harteman JC, Groenendaal F, van Haastert IC, Liem KD, Stroink H, Bierings MB, Huisman A, de Vries LS. Atypical timing and presentation of periventricular haemorrhagic infarction in preterm infants: the role of thrombophilia. Dev Med Child Neurol. 2012;54:140- 147.
129. Hashem H, Egler R, Dalal J. Refractory Pure Red Cell Aplasia Manifesting as Deficiency of Adenosine Deaminase 2. J Pediatr Hematol Oncol. 2017;39:e293-6.
130. Hashem H, Kumar AR, Müller I, Babor F, Bredius R, Dalal J, Hsu AP, Holland SM, Hickstein DD, Jolles S et al. Hematopoietic stem cell transplantation rescues the hematological, immunological, and vascular phenotype in DADA2. Blood. 2017;130:2682- 2688.

131. Hashem H, Vatsayan A, Gupta A, Nagle K, Hershfield M, Dalal J. Successful reduced intensity hematopoietic cell transplant in a patient with deficiency of adenosine deaminase 2. Bone Marrow Transplant. 2017;52:1575-1576.
132. Hatano T, Daida K, Hoshino Y, Li Y, Saitsu H, Matsumoto N, Hatter N. Dystonia due to bilateral caudate hemorrhage associated with a COL4A1 mutation. Movement Disorders. 2017;32:823-824.(Abstract)
133. Hatano T, Daida K, Hoshino Y, Li Y, Saitsu H, Matsumoto N, Hattori N. Dystonia due to bilateral caudate hemorrhage associated with a COL4A1 mutation. Parkinsonism Relat Disord. 2017;40:80-82.
134. Hedderich DM, Lummel N, Deschauer M, Kumpfel T, Schuh E, Patzig M, Zimmer C, Huber T. Magnetic Resonance Imaging Characteristics of Retinal Vasculopathy with Cerebral Leukoencephalopathy and Systemic Manifestations. Clin Neuroradiol. 2019;1-8.
135. Heinrich T, Dufke A, Waldmuller S, Schoning M. Identification of a COL4A1 mutation in a boy with developmental delay, brain malformations, and bilateral cataract allows for prenatal testing in a subsequent pregnancy. Medizinische Genetik. 2016;28:153. (Abstract)
136. Hinman JD, Lee MD, Tung S, Vinters HV, Carmichael ST. Molecular disorganization of axons adjacent to human lacunar infarcts. Brain. 2015;138:736-745.
137. Hoffmann P, Ombrello AK, Stone DL, Barron K, Pinto-Patarroyo G, Jones A, Romeo T, Follmann D, Toro C, Soldatos A et al. Analysis of the use of anticoagulants and antiplatelet agents in strokes caused by the deficiency of adenosine deaminase 2. Arthritis Rheumatol. 2016;68:3110-3111. (Abstract)
138. Hoffmann PM, Ombrello A, Stone DL, Follmann D, Barron K, Jones A, Romeo T, Toro C, Soldatos A, Hay A et al. Risk of hemorrhagic strokes in patients with adenosine deaminase 2 deficiency. Arthritis Rheumatol. 2018;70:2517. (Abstract)
139. Hsu AP, West RR, Calvo KR, Cuellar-Rodriguez J, Parta M, Kelly SJ, Ganson NJ, Hershfield MS, Holland SM, Hickstein DD. Adenosine deaminase type 2 deficiency masquerading as GATA2 deficiency: Successful hematopoietic stem cell transplantation. Ann Rheum Dis. 2017;76:1648-1656.
140. Hsu AP, West RR, Calvo KR, Cuellar-Rodriguez J, Parta M, Kelly SJ, Ganson NJ, Hershfield MS, Holland SM, Hickstein DD. Adenosine deaminase type 2 deficiency masquerading as GATA2 deficiency: Successful hematopoietic stem cell transplantation. J. Allergy Clin. Immunol. 2016;138:628-630.e2.
141. Hwang YT, Lakshmanan R, Davagnanam I, Thompson AGB, Lynch DS, Houlden H, Bajaj N, Eriksson SH, Bamiou DE, Warren JD. Brainstem phenotype of cathepsin A-related arteriopathy with strokes and leukoencephalopathy. Neurol Genet. 2017;3:e165.
142. Ibrahimi M, Nozaki H, Lee A, Onodera O, Reichwein R, Wicklund M, El-Ghanem M. A CARASIL Patient from Americas with Novel Mutation and Atypical Features: Case Presentation and Literature Review. Cerebrovasc Dis. 2017;44:135-140.
143. "Insalaco A, Moneta G, Pardeo M, Passarelli C, Celani C, Messia V, De Benedetti F. Variable clinical phenotypes and relation of interferon signature with disease activity in
144. ADA 2 deficiency. Pediatr Rheumatol. 2017;15. (Abstract)"

145. Insalaco A, Moneta G, Pardeo M, Passarelli C, Celani C, Messia V, De Benedetti F. Variable clinical phenotypes and relation of interferon signature with disease activity in ADA 2 deficiency. Arthritis Rheumatol. 2016;68:3111-3112. (Abstract)
146. Insalaco A, Moneta GM, Pardeo M, Caiello I, Messia V, Bracaglia C, Passarelli C, De Benedetti F. Variable Clinical Phenotypes and Relation of Interferon Signature with Disease Activity in ADA2 Deficiency. J Rheumatol. 2019;46:523-526.
147. Ito J, Nozaki H, Toyoshima Y, Abe T, Sato A, Hashidate H, Igarashi S, Onodera O, Takahashi H, Kakita A. Histopathologic features of an autopsied patient with cerebral small vessel disease and a heterozygous HTRA1 mutation. Neuropathology. 2018;38:428-432.
148. Ito S, Takao M, Fukutake T, Hatsuta H, Funabe S, Ito N, Shimoe Y, Niki T, Nakano I, Fukayama M et al. Histopathologic Analysis of Cerebral Autosomal Recessive Arteriopathy with Subcortical Infarcts and Leukoencephalopathy (CARASIL): A Report of a New Genetically Confirmed Case and Comparison to 2 Previous Cases. J Neuropathol Exp Neurol. 2016;75:1020-1030.
149. Ito S, Takao M, Nogami A, Funabe S, Hatsuta H, Niki T, Ito N, Fukutake T, Shimoe Y, Fukayama M, et al. Cerebral autosomal recessive arteriopathy with subcortical infarcts and leukoencephalopathy (CARASIL):neuropathological examinations of three genetically confirmed autopsied cases. Neuropathology. 2012;32:363. (Abstract)
150. Jeanne M, Labelle-Dumais C, Jorgensen J, Kauffman WB, Mancini GM, Favor J, Valant V, Greenberg SM, Rosand J, Gould DB. COL4A2 mutations impair COL4A1 and COL4A2 secretion and cause hemorrhagic stroke. Am J Hum Genet. 2012;90:91-101.
151. John S, Jehi L, Manno EM, Conway DS, Uchino K. COL4A1 gene mutation--beyond a vascular syndrome. Seizure. 2015;31:19-21.
152. John S, Jehi L, Manno EM, Conway DS, Uchino K. COL4A1 gene mutation--beyond a vascular syndrome. Seizure. 2015;31:19-21.
153. Jordan MA, Pierpont ME, Johnston RH, Lee MS, Mcclelland CM. Hereditary angiopathy with nephropathy, aneurysm, and muscle cramps (hanac) syndrome presenting to neuro-ophthalmology with metamorphopsia. J. Neuroophthalmol. 2019;39:506–510.
154. Kaljas Y, Liu C, Skaldin M, Wu C, Zhou Q, Lu Y, Aksentijevich I, Zavialov AV. Human adenosine deaminases ADA1 and ADA2 bind to different subsets of immune cells. Cell. Mol. Life Sci. 2017;74:555–570.
155. Kamo H, Conedera SA, Ogaki K, Daida K, Li Y, Funabashi M, Yoshino H, Funayama M, Nishioka K, Hattori N. Genetic analyses of HTRA1 and CTSA in Japanese patients with cerebral small vessel disease. Clin. Neurol. 2019;59:S409. (Abstract)
156. Karacan İ, Balamir A, Uğurlu S, Aydın AK, Everest E, Zor S, Önen M, Daşdemir S, Özkaya O, Sözeri B et al. Diagnostic utility of a targeted next-generation sequencing gene panel in the clinical suspicion of systemic autoinflammatory diseases: a multi-center study. Rheumatol Int. 2019;39:911-919.
157. Keer N, Hershfield M, Caskey T, Unizony S. Novel compound heterozygous variants in CECR1 gene associated with childhood onset polyarteritis nodosa and deficiency of ADA2. Rheumatology. 2016;55:1145-1147.
158. Kellett S, Lemaire M, Miller SP, Licht C, Yoon G, Dlamini N, Noone D. Neonatal stroke and haematuria: Answers. Pediatr Nephrol. 2018;33:807-811.

159. Kellett S, Lemaire M, Miller SP, Licht C, Yoon G, Dlamini N, Noone D. Neonatal stroke and haematuria: Questions. Pediatr Nephrol. 2018;33:805-806.
160. Khaleeli Z, Jaunmuktane Z, Beaufort N, Houlden H, Haffner C, Brandner S, Dichgans M, Werring D. A novel HTRA1 exon 2 mutation causes loss of protease activity in a Pakistani CARASIL patient. J Neurol. 2015;262:1369-1372.
161. Khalid R, Krishnan P, Andres K, Blaser S, Miller S, Moharir M, Dlamini N. COL4A1 and fetal vascular origins of schizencephaly. Neurology. 2018;90:232-234.
162. Khalid R, Krishnan P, Blaser S, Andres K, Miller S, Moharir M, Dlamini N. Fetal vascular origins of schizencephaly. Ann Neurol. 2016;80:S341-S343. (Abstract)
163. Kilic SS, Cekic S, Karali Y. Cerebral ischemic attacks in ADA2 deficiency treated with adalimumab. Allergy Eur. J. Allergy Clin. Immunol. 2019;74:828. (Abstract) (a)
164. Kilic SS, Cekic S, Karali Y. Severe neutropenia in ADA2 deficiency. Arch. Dis. Child. 2019;104:A304. (Abstract) (b)
165. Kinoshita K, Ishizaki Y, Yamamoto H, Sonoda M, Yonemoto K, Kira R, Sanefuji M, Ueda A, Matsui H, Ando Y, et al. De novo p.G696S mutation in COL4A1 causes intracranial calcification and late-onset cerebral hemorrhage: A case report and review of the literature COL4A1-associated vasculopathy. Eur. J. Med. Genet. 2020;63:103825.
166. Kitzler TM, Schneider R, Kohl S, Kolvenbach CM, Connaughton DM, Dai R, Mann N, Nakayama M, Majmundar AJ, Wu CW et al. COL4A1 mutations as a potential novel cause of autosomal dominant CAKUT in humans. Hum Genet. 2019;138:1105-1115.
167. Klemans RJB, Leavis HL, van Montfrans JM, van Dijk MR, Sanders CJG. Recurrent painful ulcers. Ned. Tijdschr. Voor Dermatol. En Venereol. 2019;29:46–48.
168. Koerber I, Kudernatsch M, Hartlieb T, Selch C, Sisodiya S, Coras R, Blumcke I, Winkler P, Berweck S, Kluger G. Histopathology and MRI findings in two children with COL4A1/-2 mutation related epilepsy. Epilepsia. 2018;59:S188. (Abstract)
169. Kollmann P, Peeters A, Vanakker O, Sznajer Y. 'De novo' Col4A2 mutation in a patient with migraine, leukoencephalopathy, and small carotid aneurysms. J Neurol. 2016;263:2327-2329.
170. Komaki R, Ueda T, Tsuji Y, Miyawaki T, Kusuhara S, Hara S, Toda T. Retinal vasculopathy with cerebral leukoencephalopathy carrying TREX1 mutation diagnosed by the intracranial calcification: a case report. Rinsho Shinkeigaku. 2018;58:111-117.
171. Kono Y, Nishioka K, Komatuzaki Y, Ito Y, Yoshino H, Tanaka R, Hattori N, Iguchi Y. CADASIL type 2 in two families prsenting mimic symptoms of CARASIL. J Neurol Sci. 2017;381. (Abstract)
172. Kono Y, Nishioka K, Li Y, Komatuzaki Y, Ito Y, Yoshino H, Tanaka R, Iguchi Y, Hattori N. Heterozygous HTRA1 mutations with mimicking symptoms of CARASIL in two families. Clin Neurol Neurosurg. 2018;172:174-176.
173. Konstantoulaki E, Siddiqui A, Amaya L, Gowda V. Case presentation: Developmental delay, cataracts and seizures with white matter infarctions explained by a novel COL4A1 mutation on a young child. Dev Med Child Neurol. 2017;59:93.(Abstract) (a)
174. Konstantoulaki E, Siddiqui A, Livingston J, Gowda V. Novel mutation in COL4A1 in a boy with cataracts, gross motor and speech delay, seizures, stroke and deep white matter changes on neuroimaging. Dev Med Child Neurol. 2017;59:99. (Abstract) (b)
175. Krutzke S, Horneff G. Treatment of Two Male Children Suffering From Deficiency of Adenosine Deaminase Type 2 (DADA2) With TNF-Inhibitor Etanercept. J. Clin. Rheumatol. Pract. Rep. Rheum. Musculoskelet. Dis. 2019;Publish Ahead of Print.

176. Kumar AR, Hickstein DD, Ghadir SS, Bertuch AA, Krance RA, Hsu AP, Hashem H, Babor F, Meisel R, Koskenvuo M et al. Hematopoietic stem cell transplantation rescues the vascular, haematological and immunological phenotype in adenosine deaminase 2 deficiency. J Clin Immunol. 2017;37:247-248.
177. Kunii M, Doi H, Kubota S, Hashiguchi S, Hirama N, Ogawa Y, Takahashi K, Tanaka K, Tada M et al. Genetic analysis of adult leukoencephalopathy patients using whole exon sequencing. J Neurol Sci. 2017;381:455. (Abstract)
178. Labauge P, Carra-Dalliere C, Ayrignac X, De Champfleur NM, Aubourg P, Bellesme C, Pelletier J, Audoin B, De Seze J, Collongues N, et al. Diagnosis of adult onset leukodystrophy in a consecutive study of 156 patients. Neurology. 2013;80:1 MeetingAbstracts. (Abstract)
179. Labelle-Dumais C, Dilworth DJ, Harrington EP, de Leau M, Lyons D, Kabaeva Z, Manzini MC, Dobyns WB, Walsh CA, Michele DE et al. COL4A1 mutations cause ocular dysgenesis, neuronal localization defects, and myopathy in mice and Walker-Warburg syndrome in humans. PLoS Genet. 2011;7:e1002062.
180. Labelle-Dumais C, Schuitema V, Hayashi G, Hoff K, Gong W, Dao DQ, Ullian EM, Oishi P, Margeta M, Gould DB. COL4A1 Mutations Cause Neuromuscular Disease with Tissue-Specific Mechanistic Heterogeneity. Am J Hum Genet. 2019;104:847-860.
181. Lamprecht P, Humrich JY, Diebold I, Riemekasten G. Diagnosis of deficiency of adenosine deaminase 2 with early onset polyarteritis nodosa in an adult patient with a novel compound heterozygous CECR1 mutation. Clin Exp Rheumatol. 2018;36:177.
182. Lee PY, Kellner ES, Huang Y, Furutani E, Huang Z, Bainter W, Alosaimi MF, Stafstrom K, Platt CD, Stauber T, et al. Genotype and functional correlates of disease phenotype in deficiency of adenosine deaminase 2 (DADA2). J. Allergy Clin. Immunol. 2020;145:1664–1672.
183. Lee YC, Chung CP, Chao NC, Fuh JL, Chang FC, Soong BW, Liao YC. Characterization of Heterozygous HTRA1 Mutations in Taiwanese Patients with Cerebral Small Vessel Disease. Stroke. 2018;49:1593-1601.
184. Lee YC, Huang Y, Zhou Q, Schnappauf O, Hershfield MS, Li Y, Ganson NJ, Sampaio Moura N, Delmonte OM, Stone SS et al. Disrupted N-linked glycosylation as a disease mechanism in deficiency of ADA2. J Allerg Clin Immunol. 2018;142:1363-1365.
185. Lemmens R, Maugeri A, Niessen HW, Goris A, Tousseyn T, Demaerel P, Corveleyn A, Robberecht W, van der Knaap MS, Thijs VN et al. Novel COL4A1 mutations cause cerebral small vessel disease by haploinsufficiency. Hum Mol Genet. 2013;22:391-397.
186. Leung M, Lewis EC, Humphreys P, Miller E, Geraghty M, Lines M, Sell E. COL4A1 mutation in a pediatric patient presenting with post-ictal hemiparesis. Can J Neurol Sci. 2012;39:654-657.
187. Leung M, Lewis EC, Humphreys P, Miller E, Lines M, Sell E. COL4A1 mutation in a pediatric patient presenting with a Todd's paresis. Can J Neurol Sci. 2011;38:S68-S69. (Abstract)
188. Li WR, Zhao DH, Wang ZX, Hong DJ, Zhang W, Yuan Y. Novel mutation of HTRA1 gene causes cerebral autosomal recessive arteriopathy with subcortical infarcts and leukoencephalopathy: one case. Chinese Journal of Neurology. 2012;45:566-569.
189. Liao YC, Chao NC, Lee YC. Heterozygous HTRA1 mutation in Taiwanese patients with cerebral small vessel disease. Neurology. 2017;88(16). (Abstract) (a)

190. Liao YC, Chao NC, Tsai PC, Soong BW, Lee YC. Heterozygous HTRA1 mutation in Taiwanese patients with cerebral small vessel disease. J Neurol Sci. 2017;381:456. (Abstract) (b)
191. Lichtenbelt KD, Pistorius LR, De Tollenaer SM, Mancini GM, De Vries LS. Prenatal genetic confirmation of a COL4A1 mutation presenting with sonographic fetal intracranial hemorrhage. Ultrasound Obstet Gynecol. 2012;39:726-727.
192. Liebowitz J, Hellmann DB, Schnappauf O. Thirty Years of Follow up in 3 Patients with Familial Polyarteritis Nodosa due to Adenosine Deaminase 2 Deficiency. J Rheumatol. 2019;46:1059-1060.
193. Liu L, Wang W, Wang Y, Hou J, Ying W, Hui X, Zhou Q, Liu D, Yao H, Sun J et al. A Chinese DADA2 patient: report of two novel mutations and successful HSCT. Immunogenetics. 2019;71:299-305.
194. Livingston J, Doherty D, Orcesi S, Tonduti D, Piechiecchio A, La Piana R, Tournier-Lasserve E, Majumdar A, Tomkins S, Rice G et al. COL4A1 mutations associated with a characteristic pattern of intracranial calcification. Neuropediatrics. 2011;42:227-233.
195. Livingston JH, Stivaros S, van der Knaap MS, Crow YJ. Recognizable phenotypes associated with intracranial calcification. Dev Med Child Neurol. 2013;55:46-57.
196. Loureiro G, Oliveira D, Ganhao S, Aguiar F, Rodrigues M, Brito I. ADA2 deficiency presenting as infantile polyarteritis nodosa. Ann. Rheum. Dis. 2019;78:1985.
197. Low WC, Junna M, Börjesson-Hanson A, Morris CM, Moss TH, Stevens DL, St Clair D, Mizuno T, Zhang WW, Mykkänen K et al. Hereditary multi-infarct dementia of the Swedish type is a novel disorder different from NOTCH3 causing CADASIL. Brain. 2007;130:357-367.
198. Low WC, Junna M, Börjesson-Hanson A, Morris CM, Moss TH, Stevens DL, St Clair D, Mizuno T, Zhang WW, Mykkänen K et al. Hereditary multi-infarct dementia of the Swedish type is a novel disorder different from NOTCH3 causing CADASIL. Brain. 2007;130:357-367.
199. Lynch D, De Paiva ARB, Zhang WJ, Lakshmanan R, Davagnanam I, Fox N, Murphy E, Kok F, Chataway J, Houlden H. Clinical and genetic characterisation of adult onset leukoencephalopathy. Neurology. 2017;88:16 Supplement 1. (Abstract)
200. Lynch DS, Rodrigues Brandão de Paiva A, Zhang WJ, Bugiardini E, Freua F, Tavares Lucato L, Macedo-Souza LI, Lakshmanan R, Kinsella JA, Merwick A et al. Clinical and genetic characterization of leukoencephalopathies in adults. Brain. 2017;140:1204-1211.
201. Maccora I, Frongia I, Azzari C, Ricci S, Cimaz R, Simonini G. A misleading case of deficiency of adenosine deaminase 2 (DADA2): the magnifying glass of the scientific knowledge drives the tailored medicine in real life. Clin Exp Rheumatol. 2018;36:146.
202. Magnin E, Ayrignac X, Berger E, Mine M, Tournier-Lasserve E, Labauge P. Late diagnosis of COL4A1 mutation and problematic vascular risk factor management. Eur Neurol. 2014;72:150-152.
203. Maisonneuve E, M’Barek IB, Leblanc T, Da Costa L, Friszer S, Pernot F, Thomas P, Castaigne V, N’Dour CT, Mailloux A, et al. Managing the Unusual Causes of Fetal Anemia. Fetal Diagn. Ther. 2020;47:156–164.
204. Mancini GM, de Coo IF, Lequin MH, Arts WF. Hereditary porencephaly: clinical and MRI findings in two Dutch families. Eur J Paediatr Neurol. 2004;8:45-54.

205. Martin H, Bursztejn AC, Cuny JF, Sarrabay G, Schmutz JL, Touitou I, Wahl D, Bonhomme A. Chronic leg ulcer revealing adenosine deaminase 2 deficiency: an atypical presentation. Eur J Dermatol. 2018;28:847-848.
206. Mateen FJ, Krecke K, Younge BR, Ford AL, Shaikh A, Kothari PH, Atkinson JP. Evolution of a tumor-like lesion in cerebroretinal vasculopathy and TREX1 mutation. Neurology. 2010;75:1211-1213.
207. Matias-Perez D, Garcia-Montano LA, Cruz-Aguilar M, Garcia-Montalvo IA, Nava-Valdez J, Barragan-Arevalo T, Villanueva-Mendoza C, Villarroel CE, Guadarrama-Vallejo C, la Cruz RV-D et al. Identification of novel pathogenic variants and novel gene-phenotype correlations in Mexican subjects with microphthalmia and/or anophthalmia by next- generation sequencing. J Hum Genet. 2018;63:1169-1180.
208. Matsumoto T, Miyakoshi K, Fukutake M, Ochiai D, Minegishi K, Tanaka M. Intracranial sonographic features demonstrating in utero development of hemorrhagic brain damage leading to schizencephaly-associated COL4A1 mutation. J Med Ultrason. 2015;42:445-446.
209. Matthew S, Graf W, Szekely A. An autosomal dominant arteriopathy of the brain due to a novel mutation in collagen 4A1 gene in a family with early-onset stroke and leukoencephalopathy. Neurology. 2014;82:10 Suppl 1. (Abstract)
210. McGovern M, Flanagan O, Lynch B, Lynch SA, Allen NM. Novel COL4A2 variant in a large pedigree: Consequences and dilemmas. Clin Genet. 2017;92:447-448.
211. Mendioroz M, Fernandez-Cadenas I, del Rio-Espinola A, Rovira A, Sole E, Fernandez-Figueras MT, Garcia-Patos V, Sastre-Garriga J, Domingues-Montanari S, Alvarez-Sabin J, et al. A missense HTRA1 mutation expands CARASIL syndrome to the Caucasian population. Neurology. 2010;75:2033–2035.
212. Menezes Cordeiro I, Nzwalo H, Sá F, Ferreira RB, Alonso I, Afonso L, Basílio C. Shifting the CARASIL paradigm: report of a non-Asian family and literature review. Stroke. 2015;46:1110-1112.
213. Menter T, Winkler D, Isimbaldi G, Hopfer H, Mihatsch M. TREX1 mutations - one of the genetic causes for renal vascular diseases in younger patients. Swiss Medical Weekly. 2013;143:23S. (Abstract) (a)
214. Menter T, Winkler D, Isimbaldi G, Hopfer H, Mihatsch M. TREX1 mutations - one of the genetic causes for renal vascular diseases in younger patients. Virchows Archiv. 2013;463:298. (Abstract) (b)
215. Meuwissen ME, de Vries LS, Verbeek HA, Lequin MH, Govaert PP, Schot R, Cowan FM, Hennekam R, Rizzu P, Verheijen FW et al. Sporadic COL4A1 mutations with extensive prenatal porencephaly resembling hydranencephaly. Neurology. 2011;76:844- 846.
216. Meuwissen ME, Halley DJ, Smit LS, Lequin MH, Cobben JM, de Coo R, van Harssel J, Sallevelt S, Woldringh G, van der Knaap MS, de Vries LS, Mancini GM. The expanding phenotype of COL4A1 and COL4A2 mutations: clinical data on 13 newly identified families and a review of the literature. Genet Med. 2015;17:843-853.
217. Michniacki TF, Hannibal M, Ross CW, Frame DG, DuVall AS, Khoriaty R, Vander Lugt MT, Walkovich KJ. Hematologic Manifestations of Deficiency of Adenosine Deaminase 2 (DADA2) and Response to Tumor Necrosis Factor Inhibition in DADA2-Associated Bone Marrow Failure. J Clin Immunol. 2018;38:166-173.

218. Michniacki TF, Hannibal M, Walkovich KJ, VanderLugt MT, Hershfield M, Frame DG, DuVall AS. Bone marrow failure secondary to ADA2 deficiency in adult siblings. J Clin Immunol. 2017;37:246. (Abstract)
219. Mishra A, Chauhan G, Violleau M-H, Vojinovic D, Jian X, Bis JC, Li S, Saba Y, Grenier-Boley B, Yang Q, et al. Association of variants in HTRA1 and NOTCH3 with MRI-defined extremes of cerebral small vessel disease in older subjects. Brain. 2019;142:1009–1023.
220. Mishra A, Violleau MH, Chauhan G, Mazoyer B, Tzourio C, Debette S. Exome sequence study on extreme MRI markers of cerebral small vessel disease. Stroke. 2018;49. (Abstract)
221. Monroy-Jaramillo N, Cerón A, León E, Rivas V, Ochoa-Morales A, Arteaga-Alcaraz MG, Nocedal-Rustrian FC, Gallegos C, Alonso-Vilatela ME, Corona T. Phenotypic Variability in a Mexican Mestizo Family with Retinal Vasculopathy with Cerebral Leukodystrophy and TREX1 Mutation p.V235Gfs*6. Rev Invest Clin. 2018;70:68-75.
222. Morsi A, Maldonado A, Lal D, Moosa ANV, Pestana-Knight E, Bingaman W. Vasospasm Following Hemispherectomy: A Case Report of a Novel Complication. World Neurosurg. 2020;137:357–361.
223. Muinjonov B, Giyazitdinova E. Myelin repair correlates with CARASIL-associated neurological deficits. Eur J Neurol. 2016;23:500. (Abstract)
224. Munshi S, Eason J, Shetty AK, Sunman W, Evans A, Gruener A, Ho E, Lakhani B. COL4A1 variant presenting as recurrent stroke and cerebral small vessel disease. Int J Stroke. 2018;13:65. (Abstract)
225. Murray LS, Lu Y, Taggart A, Van Regemorter N, Vilain C, Abramowicz M, Kadler KE, Van Agtmael T. Chemical chaperone treatment reduces intracellular accumulation of mutant collagen IV and ameliorates the cellular phenotype of a COL4A2 mutation that causes haemorrhagic stroke. Hum Mol Genet. 2014;23:283-292.
226. Nagiel A, Lalane RA, Jen JC, Kreiger AE. Superficial and deep capillary ischemia as a presenting sign of retinal vasculopathy with cerebral leukoencephalopathy and systemic manifestations. Retin Cases Brief Rep. 2018;12 Suppl 1:S87-S91.
227. Naidu G, Acharya N, Jha S, Chattopadhyay A, Dhir V, Goyal M, Modi M, Nada R, Minz RW, Jain S et al. Deficiency of adenosine deaminase 2: Report of three cases from single center in North India. Indian J Rheum. 2018;13:S211-212. (Abstract)
228. Nandeesh BN, Bindu PS, Narayanappa G, Chickabasaviah Yasha T, Mahadevan A, Kulanthaivelu K, Santosh V. Cerebral small vessel disease with hemorrhagic stroke related to COL4A1 mutation: A case report. Neuropathology. 2020;40:93–98.
229. Nanthapisal S, Murphy C, Omoyinmi E, Hong Y, Standing A, Berg S, Ekelund M, Jolles S, Harper L, Youngstein T et al. Deficiency of Adenosine Deaminase Type 2: A Description of Phenotype and Genotype in Fifteen Cases. Arthritis Rheumatol. 2016;68:2314-2322.
230. Nanthapisal S, Murphy C, Omoyinmi E, Standing A, Hong Y, Gomes SM, Klein N, Eleftheriou D, Brogan PA. Monogenic polyarteritis nodosa caused by ADA2 deficiency: The GOSH experience. Pediatr Rheumatol. 2015;13:89. (Abstract)
231. Nau S, McCourt EA, Maloney JA, Van Hove JL, Saenz M, Jung JL. COL4A1 mutations in two infants with congenital cataracts and porencephaly: an ophthalmologic perspective. J. AAPOS. 2019;23:246–248.

232. Navon Elkan P, Pierce SB, Segel R, Walsh T, Barash J, Padeh S, Zlotogorski A, Berkun Y, Press JJ, Mukamel M et al. Mutant adenosine deaminase 2 in a polyarteritis nodosa vasculopathy. N Engl J Med. 2014;370:921-931.
233. Navon Elkan P, Pierce SB, Segel R, Walsh T, Barash J, Padeh S, Zlotogorski A, Berkun Y, Press JJ, Mukamel M et al. Mutant adenosine deaminase 2 in a polyarteritis nodosa vasculopathy. N Engl J Med. 2014;370:921-931.
234. Neishabury M, Mehri M, Fattahi Z, Najmabadi H, Azarkeivan A. Novel variants in Iranian individuals suspected to have inherited red blood cell disorders, including bone marrow failure syndromes. Haematologica. 2020;105:E1–E4.
235. Ng J, Gunny R, Prabhakar PS, Carr LJ, Saunders DE. The expanding neuroradiological phenotype of COL4A1 gene mutations. Dev Med Child Neurol. 2013;55:23. (Abstract)
236. Nishimoto Y, Shibata M, Nihonmatsu M, Nozaki H, Shiga A, Shirata A, Yamane K, Kosakai A, Takahashi K, Nishizawa M et al. Neurology. 2011;76:1353-1355. (a)
237. Nishimoto Y, Shibata M, Onodera O, Suzuki N. Neurological picture. Neuroaxonal integrity evaluated by MR spectroscopy in a case of CARASIL. J Neurol Neurosurg Psychiatry. 2011;82:860-861. (b)
238. Niwa T, Aida N, Osaka H, Wada T, Saitsu H, Imai Y. Intracranial Hemorrhage and Tortuosity of Veins Detected on Susceptibility-weighted Imaging of a Child with a Type IV Collagen α1 Mutation and Schizencephaly. Magn Reson Med Sci. 2015;14:223-226.
239. Nozaki H, Kato T, Nihonmatsu M, Saito Y, Mizuta I, Noda T, Koike R, Miyazaki K, Kaito M, Ito S et al. Distinct molecular mechanisms of HTRA1 mutants in manifesting heterozygotes with CARASIL. Clin Neurol. 2016;56:S325. (Abstract)
240. Nozaki H, Kato T, Nihonmatsu M, Saito Y, Mizuta I, Noda T, Koike R, Miyazaki K, Kaito M, Ito S et al. Distinct molecular mechanisms of HTRA1 mutants in manifesting heterozygotes with CARASIL. Neurology. 2016;86:1964-1974.
241. Nozaki H, Nishizawa M, Onodera O. Features of cerebral autosomal recessive arteriopathy with subcortical infarcts and leukoencephalopathy. Stroke. 2014;45:3447- 3453.
242. Nozaki H, Sekine Y, Fukutake T, Nishimoto Y, Shibata M, Yutaka S, Shirata A, Yanagawa S, Hirayama M, Yamane K, et al. MRI features of cerebral autosomal recessive arteriopathy with subcortical infarcts and leukoencephalopathy. Neurology. 2013;80:1 MeetingAbstracts. (Abstract)
243. Nozaki H, Sekine Y, Fukutake T, Nishimoto Y, Shimoe Y, Shirata A, Yanagawa S, Hirayama M, Tamura M, Nishizawa M et al. Characteristic features and progression of abnormalities on MRI for CARASIL. Neurology. 2015;85:459-463.
244. O’Neill R, O’Mahony O, McSweeney N. COL4A1 mutation inherited from maternal mosaicism in an infant presenting with microcephaly, haemolytic anaemia and cataracts. Arch. Dis. Child. 2019;104:A126–A127.
245. Ohta K, Ozawa T, Fujinaka H, Goto K, Nakajima T. Cerebral Small Vessel Disease Related to a Heterozygous Nonsense Mutation in HTRA1. Intern. Med. 2020;59:1309–1313.
246. Okano S, Shimada S, Tanaka R, Okayama A, Kajihama A, Suzuki N, Nakau K, Takahashi S, Matsumoto N, Saitsu H, et al. Life-threatening muscle complications of COL4A1-related disorder. Brain Dev. 2020;42:93–97.

247. Oluwole OJ, Ibrahim H, Garozzo D, Ben Hamouda K, Ismail Mostafa Hassan S, Hegazy AM, Msaddi AK. Cerebral small vessel disease due to a unique heterozygous HTRA1 mutation in an African man. Neurol. Genet. 2020;6:e382.
248. Ombrello A, Stone D, Hoffmann P, Jones A, Barham B, Barron K, Flegel W, Sheldon S, Zhou Q, Hershfield M et al. The deficiency of adenosine deaminase type 2-results of therapeutic intervention. Pediatr Rheumatol. 2015;13:37. (Abstract)
249. Ombrello AK, Barron K, Hoffmann P, Toro C, Stone DL, Pinto-Patarroyo G, Jones A, Romeo T, Soldatos A, Zhou Q et al. The deficiency of adenosine deaminase type 2 (DADA2)-results of anti-TNF treatment in a cohort of patients with a history of stroke. Arthritis Rheumatol. 2016;68:4288-4289. (Abstract)
250. Ombrello AK, Stone DL, Barron K, Hoffmann PM, Cudrici C, Jones A, Romeo T, Dimitrova D, Dotan A, Wall D et al. Analysis of the efficacy of treatment on 45 patients with deficiency of adenosine deaminase 2. Pediatr Rheumatol. 2019;17. (Abstract)
251. Ouail DE, Tebbani M, Si Ahmed D, Bouali F. Youth hypertension associated with ADA2 deficiency. About three cases. J. Hypertens. 2019;37:e215. (Abstract)
252. Ozen S, Batu ED, Taskiran EZ, Ozkara HA, Unal S., Guleray N, Erden A, Karadag O, Gumruk F, Cetin M, et al. A monogenic disease with a variety of phenotypes: Deficiency of adenosine deaminase 2. J. Rheumatol. 2020;47:117–125.
253. Paisal V, Al-Abadi E, Southwood T, Wassmer E. Childhood onset stroke and vasculitis associated with deficiency of adenosine deaminase 2 (dada2). Ann Rheum Dis. 2017;76:1395. (Abstract)
254. Paola K, Gomes FHR, Benevides LC, Leite MF, Medeiros P, Santos AC, De Carvalho LM, Ferriani V. CECR1/ADA2 mutation in a Brazilian family. Ann. Rheum. Dis. 2019;78:2009. (Abstract)
255. Papandreou A, Tisdall MM, Chong WK, Cross JH, Harkness WF, Varadkar SM. COL4A1 mutations should not be a contraindication for epilepsy surgery. Childs Nerv Syst. 2014;30:1467-1469.
256. Papandreou A, Tisdall MM, Harkness WF, Cross JH, Varadkar SM. COL4A1 mutations should not be a contraindication for epilepsy surgery. Epilepsia. 2014;55:245. (Abstract)
257. Pati AR, Battisti C, Taglia I, Galluzzi P, Bianchi M, Federico A. A new case of autosomal dominant small vessel disease carrying a novel heterozygous mutation in HTRA1 gene: 2-year follow-up. Neurol Sci. 2018;39:1479-1481.
258. Pelzer N, Bijkerk R, Reinders MEJ, van Zonneveld AJ, Ferrari MD, van den Maagdenberg AMJM, Eikenboom J, Terwindt GM. Circulating Endothelial Markers in Retinal Vasculopathy With Cerebral Leukoencephalopathy and Systemic Manifestations. Stroke. 2017;48:3301-3307.
259. Pelzer N, Hoogeveen ES, Haan J, Bunnik R, Poot CC, van Zwet EW, Inderson A, Fogteloo AJ, Reinders MEJ, Middelkoop HAM et al. Systemic features of retinal vasculopathy with cerebral leukoencephalopathy and systemic manifestations: a monogenic small vessel disease. J Intern Med. 2019;285:317-332.
260. Pescini F, Donni I, Asaro A, Rinnoci V, Squitieri M, Nannucci S, Poggesi A, Di Donoato I, Bianchi S, Cereda C, et al. Screening for COL4A1 and COL4A2 mutations in patients with familiar microangiopathy. Eur. Stroke J. 2019;4:699–700.

261. Pichard DC, Ombrello AK, Hoffmann P, Stone DL, Cowen EW. Early-onset stroke, polyarteritis nodosa (PAN), and livedo racemosa. J Am Acad Dermatol. 2016;75:449-453.
262. Plaisier E, Chen Z, Gekeler F, Benhassine S, Dahan K, Marro B, Alamowitch S, Paques M, Ronco P. Novel COL4A1 mutations associated with HANAC syndrome: a role for the triple helical CB3[IV] domain. Am J Med Genet. 2010;152A:2550-2555.
263. Plaisier E, Gribouval O, Alamowitch S, Mougenot B, Prost C, Verpont MC, Marro B, Desmettre T, Cohen SY, Roullet E. et al. COL4A1 mutations and hereditary angiopathy, nephropathy, aneurysms, and muscle cramps. N Engl J Med. 2007;357:2687-2695.
264. Plancher JM, Hufnagel RB, Vagal A, Peariso K, Saal HM, Broderick JP. Case of Small Vessel Disease Associated with COL4A1 Mutations following Trauma. Case Rep Neurol. 2015;7:142-147.
265. Plancher JM, Hufnagel RB, Vagal A, Peariso K, Saal HM, Broderick JP. Case of Small Vessel Disease Associated with COL4A1 Mutations following Trauma. Case Rep Neurol. 2015;7:142-147.
266. Pode-Shakked B, Marek-Yagel D, Navon-Elkan P, Pierce SB, Segel R, Walsh T, Padeh S, Fairbanks L, Pras E, Winkelmann J et al. Adenosine deaminase 2 (ADA2) deficiency: A novel inborn error of purine metabolism. Mol Genet Metab. 2014;111:232- 233.
267. Poswar F, Da Fonseca RM, de Albuquerque LC, Zhou Q, Jardim LB, Monte TL, Aksentijevich I, Saute JA. Adenosine deaminase 2 deficiency presenting as spastic paraplegia and systemic vasculitis. J Neurol. 2016;263:818-820.
268. Preethish-Kumar V, Nozaki H, Tiwari S, Vengalil S, Bhat M, Prasad C, Onodera O, Uemura M, Doniparthi S, Saini J et al. CARASIL families from India with 3 novel null mutations in the HTRA1 gene. Neurology. 2017;89:2392-2394.
269. Rama M, Duflos C, Melki I, Bessis D, Bonhomme A, Martin H, Doummar D, Valence S, Rodriguez D, Carme E et al. A decision tree for the genetic diagnosis of deficiency of adenosine deaminase 2 (DADA2): a French reference centres experience. Eur J Hum Genet. 2018;26:960-971.
270. Rasmussen M, Hareide LL, Skogen AR, Nedregaard B, Antal E-A, Plaisier E. Work- up of an increased level of CK leading to the diagnosis of HANAC. Eur J Paed Neurol. 2017;21:e226. (Abstract)
271. Raynowska J, Miskin DP, Pramanik B, Asiry S, Anderson T, Boockvar J, Najjar S, Harel A. Retinal vasculopathy with cerebral leukoencephalopathy (RVCL): A rare mimic of tumefactive MS. Neurology. 2018;91:e1423-1428.
272. Richards A, van den Maagdenberg AM, Jen JC, Kavanagh D, Bertram P, Spitzer D, Liszewski M, Barilla-Labarca ML, Terwindt GM, Kasai Y et al. C-terminal truncations in human 3'-5' DNA exonuclease TREX1 cause autosomal dominant retinal vasculopathy with cerebral leukodystrophy. Nat Genet. 2007;39:1068-1670.
273. Riley CS, Roth LA, Sampson JB, Radhakrishnan J, Herlitz LC, Blitz AM, Moazami G. A 31-Year-Old Man With a Ring-Enhancing Brain Lesion. J Neuroophthalmol. 2017;37:172-175.
274. Rødahl E, Knappskog PM, Majewski J, Johansson S, Telstad W, Kråkenes J, Boman H. Variants of anterior segment dysgenesis and cerebral involvement in a large family with a novel COL4A1 mutation. Am J Ophthalmol. 2013;155:946-953.
275. Roeben B, Uhrig S, Bender B, Synofzik M. Teaching NeuroImages: When alopecia and disk herniations meet vascular leukoencephalopathy Neurology. 2016;86:e166.

276. Rouaud T, Labauge P, Tournier Lasserve E, Mine M, Coustans M, Deburghgraeve V, Edan G. Acute urinary retention due to a novel collagen COL4A1 mutation. Neurology. 2010;75:747-749.
277. Ruiz-Escribano Menchen L, Flores Barragan JM, Camacho Nieto A, Franco Salinas AR, Villanueva Ruiz FJ, Hernandez Gonzalez A, Vaamonde Gamo J. COL4A1 novel missense mutation causing recurrent spontaneous intracerebral haemorrhage and encephalopathy. Eur. Stroke J. 2019;4:364. (Abstract)
278. Russo A, Pinto AM, Lopergolo D, Renieri A, Battisti C. An Italian family carrying a new mutation in the COL4A1 gene. J. Neurol. Sci. 2020;414:116815.
279. Saffari A, Kolker S, Merkenschlager A, Hoffmann GF, Ziegler A, Syrbe S. Axenfeld-rieger anomaly and neuropsychiatric symptoms. Neuropediatrics. 2018;49:2. (Abstract)
280. Saffari A, Ziegler A, Merkenschlager A, Kruger S, Kolker S, Hoffmann GF, Syrbe S. Axenfeld-Rieger Anomaly and Neuropsychiatric Problems-More than Meets the Eye. Neuropediatrics. 2020;51:192–197.
281. Sahin S, Adrovic A, Barut K, Baran S, Tahir Turanli E, Canpolat N, Kizilkilic O, Ozkaya O, Kasapcopur O. A 9.5-year-old boy with recurrent neurological manifestations and severe hypertension, treated initially for polyarteritis nodosa, was subsequently diagnosed with adenosine deaminase type 2 deficiency (DADA2) which responded to anti- TNF-α. Paediatr Int Child Health. 2019;1-4.
282. Sahin S, Adrovic A, Barut K, Baran S, Tahir Turanli E, Canpolat N, Kizilkilic O, Ozkaya O, Kasapcopur O. A 9.5-year-old boy with recurrent neurological manifestations and severe hypertension, treated initially for polyarteritis nodosa, was subsequently diagnosed with adenosine deaminase type 2 deficiency (DADA2) which responded to anti-TNF-alpha. Paediatr. Int. Child Health. 2020;40:65–68.
283. Sahin S, Adrovic A, Barut K, Ugurlu S, Turanli ET, Ozdogan H, Kasapcopur O. Anti TNF-alpha therapy would be lifesaving in deficiency of adenosine deaminase-2. Ann Rheumat Dis. 2017; 76:1402-1403. (Abstract)
284. Sahin S, Adrovic A, Barut K, Ugurlu S, Turanli ET, Ozdogan H, Kasapcopur O. Clinical, imaging and genotypical features of three deceased and five surviving cases with ADA2 deficiency. Rheumatol Int. 2018;38:129-136.
285. Saito R, Nozaki H, Kato T, Toyoshima Y, Tanaka H, Tsubata Y, Morioka T, Horikawa Y, Oyanagi K, Morita T et al. Retinal Vasculopathy With Cerebral Leukodystrophy: Clinicopathologic Features of an Autopsied Patient With a Heterozygous TREX 1 Mutation. J Neuropathol Exp Neurol. 2019;78:181-186.
286. Saitsu H, Yoneda Y, Haginoya K, Arai H, Yamaoka S, Matsumoto N. De novo and inherited mutations in COL4A2, encoding the type IV collagen alpha2 chain, cause porencephaly. Congenital Anomalies. 2012;52:A8-A9. (Abstract)
287. Sakai N, Uemura M, Kato T, Nozaki H, Koyama A, Ando S, Kamei H, Kato M, Onodera O. Hemorrhagic cerebral small vessel disease caused by a novel mutation in 3’ UTR of collagen type IV alpha 1. Neurol. Genet. 2020;6:e383.
288. Santo GC, Baldeiras I, Guerreiro R, Ribeiro JA, Cunha R, Youngstein T, Nanthapisal S, Leitão J, Fernandes C, Caramelo F et al. Adenosine Deaminase Two and Immunoglobulin M Accurately Differentiate Adult Sneddon's Syndrome of Unknown Cause. Cerebrovasc Dis. 2018;46:257-264.

289. Sarkar K, Way C, Verro P. A case of retinal vasculopathy and cerebral leukodystrophy with predominantly central nervous system manifestations. Neurology. 2012;78. (Abstract)
290. Sarrabay G, Insalaco A, Uettwiller F, Tieulie N, Quartier-Dit-Maire P, Melki J, Touitou I. Identification of three ADA2 deficiency families with novel CECR1 mutations. Ped Rheumatol. 2015;13:229. (Abstract)
291. Sasa GS, Elghetany MT, Bergstrom K, Nicholas S, Himes R, Krance RA, Hershfield M, Van Montfrans J, Bertuch A. Adenosine deaminase 2 deficiency as a cause of pure red cell aplasia mimicking diamond blackfan anemia. Blood. 2015;126:3615. (Abstract)
292. Sasaki S, Nozaki F, Saitsu H, Miyatake S, Matsumoto N, Kumada T, Shibata M, Fujii T. A case of COL4A1 -related disorder with a variety of brain imaging findings. No To Hattatsu. 2017;49:405-407.
293. Sasaki S, Nozaki F, Saitsu H, Miyatake S, Matsumoto N, Kumada T, Shibata M, Fujii T. A case of COL4A1 -related disorder with a variety of brain imaging findings. No To Hattatsu. 2017;49:405-407.
294. Sato Y, Shibasaki J, Aida N, Hiiragi K, Kimura Y, Akahira-Azuma M, Enomoto Y, Tsurusaki Y, Kurosawa K. Novel COL4A1 mutation in a fetus with early prenatal onset of schizencephaly. Hum Genome Var. 2018;5:4.
295. Scalais E, Ceuterick-De Groot C, Martin JJ, Maugeri A, Varlet P, Devaux B, De Meirleir L. Cortical dysplasia, antenatal porencephaly, recurrent retinal hemorrhages: Different insults at different times-COL4A1 deficiency and environmental factors. Ann Neurol. 2015;78:S198. (Abstract)
296. Schena F, Pastorino C, Penco F, Volpi S, Caorsi R, Kalli F, Fenoglio D,Salis A, Prigione I, Bocca P et al. Dysregulation of B and Tfh cells functions in DADA2 patients Pediatr Rheumatol. 2018;16:2. (Abstract)
297. Schena F, Penco F, Volpi S, Pastorino C, Caorsi R, Bertoni A, Kalli F, Fenoglio D, Salis A, Prigione I et al. B cell defect in ADA2 deficiency patients. Pediatr Rheumatol. 2019;17. (Abstract)
298. Schena F, Volpi S, Caorsi R, Penco F, Pastorino C, Kalli F, Omenetti A, Chiesa S, Bertoni A, Picco P et al. Defect of adaptive immunity in ADA2 deficiency patients. Pediatr Rheumatol. 2017;15(Suppl 1). (Abstract)
299. Schepp J, Bulashevska A, Mannhardt-Laakmann W, Cao H, Yang F, Seidl M, Kelly S, Hershfield M, Grimbacher B. Deficiency of Adenosine Deaminase 2 Causes Antibody Deficiency. J Clin Immunol. 2016;36:179-186.
300. Schepp J, Proietti M, Frede N, Buchta M, Hübscher K, Rojas Restrepo J, Goldacker S, Warnatz K, Pachlopnik Schmid J, Duppenthaler A et al. Screening of 181 Patients With Antibody Deficiency for Deficiency of Adenosine Deaminase 2 Sheds New Light on the Disease in Adulthood. Arthritis Rheumatol. 2017;69:1689-1700.
301. Schnappauf O, Stoffels M, Aksentijevich I, Kastner DL, Grayson PC, Cuthbertson D, Carette S, Chung SA, Forbess LJ, Khalidi NA et al. Screening of patients with adult-onset idiopathic polyarteritis nodosa for deficiency of adenosine deaminase 2. Arthritis Rheumatol. 2018;70(suppl10). (Abstract)
302. Schnappauf O, Stoffels M, Aksentijevich I, Ombrello A, Moura NS, Barron K, Kastner D, Grayson P, Merkel P. Screening of patients with idiopathic polyarteritis nodosa, granulomatosis with polyangiitis, and microscopic polyangiitis for deficiency of adenosine deaminase 2. Pediatr Rheumatol. 2019;17. (Abstract)

303. Schnider C, Theodoropoulou K, Candotti F, Angelini F, Perreau M, Hershfield M, Hofer M. A family case of ADA 2 deficiency with CECR1 mutation. Swiss Medical Weekly. 2018;148:11S-12S. (Abstract)
304. Schnider C, Theodoropoulou K, Candotti F, Angelini F, Perreau M, Riccio O, Hershfield M, Hofer M. A family case of ADA2 deficiency with cecr1 mutation. Pediatr Rheumatol. 2018;16:P233. (Abstract)
305. Schuh E, Ertl-Wagner B, Lohse P, Wolf W, Mann JF, Lee-Kirsch MA, Hohlfeld R, Kümpfel T. Multiple sclerosis-like lesions and type I interferon signature in a patient with RVCL. Neurol Neuroimmunol Neuroinflamm. 2015;2:e55.
306. Schuh E, Lohse P, Kumpfel T. A rare case of cerebroretinal vasculopathy caused by a novel Trex 1 mutation. Journal of Neurology. 2013;260:S137. (Abstract)
307. Scoppettuolo P, Ligot N, Naeije G, Wermenbol V, Van Bogaert P. A novel mutation of COL4A1 responsible of familial porencephaly and severe hypermetropia. Eur Stroke J. 2018;3:486.
308. Segel R, Padeh S, Goldzweig O, Gerstein M, Barash J, Zlotogorski A, Pres J, Hashkes P, Horev L, Harel L, et al. Natural history and treatment outcome of patients with adenosine deaminase (ADA) 2 deficiency: Twenty years of the Israeli experience. Eur. J. Hum. Genet. 2019;26:314. (Abstract)
309. Selch C, Winkler P, Pringsheim M, Hasse A, Baumeister F, Staudt M, Kluger G. Epilepsy, clinical presentation and MRI features in patients with COL4A1 mutations. Eur J Paed Neurol. 2015;19:S6. (Abstract)
310. Severino MS, Caorsi R, Gandolfo C, Martinetti C, Martini A, Gattorno M. Distinct cerebrovascular features in patients with ADA2 deficiency. Pediatr Rheumatol. 2015;13:233. (Abstract)
311. Shah S, Ellard S, Kneen R, Lim M, Osborne N, Rankin J, Stoodley N, van der Knaap M, Whitney A, Jardine P. Childhood presentation of COL4A1 mutations. Dev Med Child Neurol. 2012;54:569-574.
312. Shah S, Kumar Y, McLean B, Churchill A, Stoodley N, Rankin J, Rizzu P, van der Knaap M, Jardine P. A dominantly inherited mutation in collagen IV A1 (COL4A1) causing childhood onset stroke without porencephaly. Eur J Paediatr Neurol. 2010;14:182-187.
313. Shan LD, Peng J, Xiao H, Wu LW, Duan HL, Pang N, Miriam K, Yin F. Clinical features and COL4A1 genotype of a toddler with hereditary angiopathy with nephropathy, aneurysms and muscle cramps syndrome. Chin. J. Contemp. Pediatr. 2019;21:754–760.
314. Sharma A, Naidu GSRSNK, Chattopadhyay A, Acharya N, Jha S, Jain S. Novel CECR1 gene mutations causing deficiency of adenosine deaminase 2, mimicking antiphospholipid syndrome. Rheumatology. 2019;58:181–182.
315. Shibata M. Clinical manifestations and neuroradiological findings of CARASIL with a novel mutation. Clinical Neurology. 2012;52:1363-1364.
316. Shwin KW, Carmona-Rivera C, Tsai W, Richard Lee CC, Novakovich E, Stone DL, Ombrello AK, Goldbach-Mansky R, Gadina M, Kastner D et al. Role of adenosine and neutrophils in inflammation associated with mutations in CECR1 gene.Arthritis Rheumatol. 2015;67. (Abstract)
317. Sibon I, Coupry I, Menegon P, Bouchet JP, Gorry P, Burgelin I, Calvas P, Orignac I, Dousset V, Lacombe D, et al. COL4A1 mutation in Axenfeld-Rieger anomaly with leukoencephalopathy and stroke. Ann Neurol. 2007;62:177-184.

318. Siitonen M, Hanson AB, Pasanen P, Bras JT, Kern S, Kern J, Andersen O, Stanescu H, Kleta R, Baumann M et al. Multi-infarct dementia of Swedish type is caused by 3'utr COL4A1 mutation. Brain. 2017;40:e29.
319. Siri A, Tournier-Lasserve E, Mine M, Magnin E, Berger E, Arquizan C, Ayrignac X, Carra-Dalliere C, Castelnovo G, De Champfleur N et al. COL4A1 mutations: Clinical and radiological phenotypes in a french adult cohort. Neurology 2014;82:10 Supplement. (Abstract)
320. Skrabl-Baumgartner A, Plecko B, Schmidt WM, König N, Hershfield M, Gruber-Sedlmayr U, Lee-Kirsch MA. Autoimmune phenotype with type I interferon signature in two brothers with ADA2 deficiency carrying a novel CECR1 mutation. Pediatr Rheumatol Online J. 2017;15:67.
321. Slavotinek AM, Garcia ST, Chandratillake G, Bardakjian T, Ullah E, Wu D, Umeda K, Lao R, Tang PL, Wan E et al. Exome sequencing in 32 patients with anophthalmia/microphthalmia and developmental eye defects. Clin Genet. 2015;88:468- 473.
322. Slavotinek AM, Garcia ST, Chandratillake G, Bardakjian T, Ullah E, Wu D, Umeda K, Lao R, Tang PL, Wan E et al. Exome sequencing in 32 patients with anophthalmia/microphthalmia and developmental eye defects. Clin Genet. 2015;88:468- 473.
323. Soldatos A, Toro C, Ombrello A, Stone D, Hoffman P, Romeo T, Jones A, Pinto-Patarroyo G, Aksentijevich I, Grayson P et al. Expanding the neurological phenotype of adenosine deaminase 2 deficiency (DADA2 syndrome) due to biallelic mutations in the CECR1 gene: A treatable pediatric lacunar stroke syndrome. Annals of Neurol. 2016;80:S338-340. (Abstract)
324. Sonmez HE, Karaaslan C, de Jesus AA, Batu ED, Anlar B, Sozeri B, Bilginer Y, Karaguzel D, Cagdas Ayvaz D, Tezcan I, et al. A clinical score to guide in decision making for monogenic type I IFNopathies. Pediatr. Res. 2020;87:745–752.
325. Sourander P, Wålinder J. Hereditary multi-infarct dementia. Morphological and clinical studies of a new disease. Acta Neuropathol. 1977;39:247-54.
326. Sozeri B, Ercan G, Dogan OA, Yildiz J, Demir F, Doganay L. Deficiency of ADA2 from childhood to adult; the same mutation in a family. Ped Rheumatol. 2019;17. (Abstract)
327. Sozeri B, Ercan G, Dogan OA, Yildiz J, Demir F, Doganay L. The same mutation in a family with adenosine deaminase 2 deficiency. Rheumatol. Int. 2019;41:227–233.
328. Springer JM, Gierer SA, Jiang H, Kleiner D, Deuitch N, Ombrello AK, Grayson PC, Aksentijevich I. Deficiency of Adenosine Deaminase 2 in Adult Siblings: Many Years of a Misdiagnosed Disease With Severe Consequences. Front Immunol. 2018;9:1361.
329. Stam AH, Kothari PH, Shaikh A, Gschwendter A, Jen JC, Hodgkinson S, Hardy TA, Hayes M, Kempster PA, Kotschet KE et al. Retinal vasculopathy with cerebral leukoencephalopathy and systemic manifestations. Brain. 2016;139:2909-2922.
330. Staples E, Simeoni I, Stephens JC, Allen HL, Wright P, Davies EG, Javid B, Gkrania-Klotsas E, Gattens M, Firth H, et al. ADA2 deficiency complicated by EBV-driven lymphoproliferative disease. Clin. Immunol. 2020;215:108443.
331. Stutterd C, Delatycki M, Lockhart P, Taft R, Vanderver A, Simons C, Leventer R. Whole-genome sequencing for patients with unclassified leukodystrophies. Twin Res. Hum. Genet. 2019;21:410–411.

332. Sundin M, Marits P, Nierkens S, Kolios AGA, Nilsson J. "Immune" Thrombocytopenia as Key Feature of a Novel ADA2 Deficiency Variant: Implication on Differential Diagnostics of ITP in Children. J Pediatr Hematol Oncol. 2019;41:155-157.
333. Takenouchi T, Ohyagi M, Torii C, Kosaki R, Takahashi T, Kosaki K. Porencephaly in a fetus and HANAC in her father: variable expression of COL4A1 mutation. Am J Med Genet A. 2015;167A:156-158.
334. Tan RYY, Traylor M, Megy K, Duarte D, Deevi SVV, Shamardina O, Mapeta RP, Consortium NBRD, Ouwehand WH, Graf S, et al. How common are single gene mutations as a cause for lacunar stroke? A targeted gene panel study. Neurology. 2019;93:e2007–e2020.
335. Tanatar A, Karadag SG, Sozeri B, Sonmez HE, Cakan M, Kendir Demirkol Y, Aktay Ayaz N. ADA2 Deficiency: Case Series of Five Patients with Varying Phenotypes. J. Clin. Immunol. 2020;40:253–258.
336. Taskinen MH, Mustjoki S, Jahnukainen K, Trotta L, Siitonen T, Hautala T, Zavialov A, Heiskanen K, Haapaniemi EM, Saarela J et al. Large granular lymphocyte infiltration in the bone marrow in children and young adults may suggest primary immune deficiency. Blood. 2015;126:1024. (Abstract)
337. Tateoka T, Onda H, Hirota K, Kasuya H, Shinohara T, Kinouchi H, Akagawa H. Unusual case of cerebral small vessel disease with a heterozygous nonsense mutation in HTRA1. J Neurol Sci. 2016;362:144-146.
338. Teixeira VA, Ramos FO, Costa M. Severe and refractory childhood-onset polyarteritis nodosa associated with CECR1 mutation. Pediatr Rheumatol. 2017;15. (Abstract)
339. Thaler FS, Catak C, Einhäupl M, Müller S, Seelos K, Wollenweber FA, Kümpfel T. Cerebral small vessel disease caused by a novel heterozygous mutation in HTRA1. J Neurol Sci. 2018;388:19-21.
340. Thomas AS, Lin P. A Case of TREX1-Associated Retinal Vasculopathy with Cerebral Leukodystrophy. Ophthalmol. Retina. 2020;4:115–117.
341. Tonduti D, Pichiecchio A, La Piana R, Livingston JH, Doherty DA, Majumdar A, Tomkins S, Mine M, Ceroni M, Ricca I et al. COL4A1-related disease: raised creatine kinase and cerebral calcification as useful pointers. Neuropediatrics. 2012;43:283-288.
342. Topkarci Z, Ayaz NA, Karadag SG, Tanatar A, Sonmez HE. Deficiency of adenosine deaminase 2 presenting as livedo racemosa. Pediatr Dermatol. 2019;36:S22. (Abstract)
343. Topkarci Z, Ayaz NA, Karadag SG, Tanatar A, Sonmez HE. Deficiency of adenosine deaminase 2 presenting as livedo rasemosa. Gazi Med. J. 2020;31:P32. (Abstract)
344. Tournier-Lasserve E, Verdura E, Herve D, Bergametti F, Jacquet C, Morvan T, Prieto-Morin C, Mackowiak A, Manchon E, Hosseini H, et al. Up-regulation of COL4A1 and COL4A2 genes through various mechanisms leads to severe early onset ischaemic smallvessel disease, including padmal. Eur. Stroke J. 2017;2:25.
345. Toz B, Erer B, Kamali S, Ocal L, Gul A. Differential response to anakinra and adalimumab in a patient with DADA2 syndrome. Pediatr Rheumatol. 2015;13:P201. (Abstract)
346. Traenka C, Kloss M, Strom T, Lyrer P, Brandt T, Bonati LH, Grond-Ginsbach C, Engelter S. Rare genetic variants in patients with cervical artery dissection. Eur. Stroke J. 2019;4:355–362.

347. Trotta L, Martelius T, Siitonen T, Hautala T, Hämäläinen S, Juntti H, Taskinen M, Ilander M, Andersson EI, Zavialov A et al. ADA2 deficiency: Clonal lymphoproliferation in a subset of patients. J Allergy Clin Immunol. 2018;141:1534-1537.e8.
348. Tsubata Y, Morita T, Morioka T, Sasagawa T, Ikarashi K, Saito N, Shimada H, Miyazaki S, Sakai S, Tanaka H et al. Renal histopathological findings of retinal vasculopathy with cerebral leukodystrophy. CEN Case Rep. 2018;7:83-89.
349. Uemura M, Nozaki H, Koyama A, Sakai N, Ando S, Kanazawa M, Kato T, Onodera O. HTRA1 Mutations Identified in Symptomatic Carriers Have the Property of Interfering the Trimer-Dependent Activation Cascade. Front. Neurol. 2019;10:693.
350. Uettwiller F, Sarrabay G, Rodero MP, Rice GI, Lagrue E, Marot Y, Deiva K, Touitou I, Crow YJ, Quartier P. ADA2 deficiency: case report of a new phenotype and novel mutation in two sisters. RMD Open. 2016;2:1-5.
351. Vahedi K, Boukobza M, Massin P, Gould DB, Tournier-Lasserve E, Bousser MG. Clinical and brain MRI follow-up study of a family with COL4A1 mutation. Neurology. 2007;69:1564-1568. (a)
352. Vahedi K, Kubis N, Boukobza M, Arnoult M, Massin P, Tournier-Lasserve E, Bousser MG. COL4A1 mutation in a patient with sporadic, recurrent intracerebral hemorrhage. Stroke. 2007;38:1461-1464. (b)
353. Van Agtmael T, Murray L, Vilain C, Abramowicz M, Kadler K. Chemical chaperone treatment influences the cellular phenotype of a COL4A2 mutation that causes haemorrhagic stroke. Cerebrovasc Dis. 2014;37:535. (Abstract)
354. Van der Knaap MS, Smit LM, Barkhof F, Pijnenburg YA, Zweegman S, Niessen HW, Imhof S, Heutink P. Ann Neurol. 2006;59:504-511.
355. Van Eyck L, Hershfield MS, Pombal D, Kelly SJ, Ganson NJ, Moens L, Frans G, Schaballie H, De Hertogh G, Dooley J et al. Hematopoietic stem cell transplantation rescues the immunologic phenotype and prevents vasculopathy in patients with adenosine deaminase 2 deficiency. J Allergy Clin Immunol. 2015;135:283-287.e5.
356. Van Eyck L, Hershfield MS, Pombal D, Kelly SJ, Ganson NJ, Moens L, Frans G, Schaballie H, DeHertogh G, Dooley J et al. HSCT rescues the immunological and vascular phenotype of ADA2-deficiency. J Clin Immunol. 2014;34:S196-197. (Abstract)
357. Van Eyck L, Liston A, Meyts I. Mutant ADA2 in vasculopathies. NEJM. 2014;371:478-479.
358. Van Eyck L, Liston A, Wouters C. Mutant ADA2 in vasculopathies (NEJM letter 2). NEJM. 2014;371:480.
359. Van Montfrans J, Hartman E, Braun K, Hennekam F, Hak A, Nederkoorn P, Westerndorp W, Bredius R, Kollen W, Scholvinck E et al. Phenotypic variability in patients with ADA2 deficiency due to identical homozygous R169Q mutations. Pediatr Rheumatol. 2015;13:1. (Abstract)
360. Van Montfrans J, Van Royen- Kerkhof A, Bierings M, Aksentijevich I, Zavialov A, Zhou Q. Hematological stem cell transplantation in ADA2 deficiency. J Clin Immunol. 2014;34:S230-231. (Abstract)
361. Van Montfrans JM, Hartman EA, Braun KP, Hennekam EA, Hak EA, Nederkoorn PJ, Westendorp WF, Bredius RG, Kollen WJ, Schölvinck EH et al. Phenotypic variability in patients with ADA2 deficiency due to identical homozygous R169Q mutations. Rheumatology. 2016;55:902-910.

362. Van Nieuwenhove E, Humblet-Baron S, Van Eyck L, De Somer L, Dooley J, Tousseyn T, Hershfield M, Liston A, Wouters C. ADA2 Deficiency Mimicking Idiopathic Multicentric Castleman Disease. Pediatrics. 2018;142:e20172266

363. van Well GTJ, Kant B, van Nistelrooij A, Sirma Ekmekci S, Henriet SV, Hoppenreijs E, van Deuren M, van Montfrans J, Nierkens S, Gul A, et al. Phenotypic variability including Behcet's disease-like manifestations in DADA2 patients due to a homozygous c.973-2A>G splice site mutation. Clin. Exp. Rheumatol. 2019;121:142–146.

364. Verbeek E, Meuwissen ME, Verheijen FW, Govaert PP, Licht DJ, Kuo DS, Poulton CJ, Schot R, Lequin MH, Dudink J et al. COL4A2 mutation associated with familial porencephaly and small-vessel disease. Eur J Hum Genet. 2012;20:844-851.

365. Verdura E, Hervé D, Bergametti F, Jacquet C, Morvan T, Prieto-Morin C, Mackowiak A, Manchon E, Hosseini H, Cordonnier C et al. Disruption of a miR-29 binding site leading to COL4A1 upregulation causes pontine autosomal dominant microangiopathy with leukoencephalopathy. Ann Neurol. 2016;80:741-753.

366. Verdura E, Hervé D, Scharrer E, Amador M, Guyant-Maréchal L, Philippi A, Corlobé A, Bergametti F, Gazal S, Prieto-Morin C et al. Heterozygous HTRA1 mutations are associated with autosomal dominant cerebral small vessel disease. Brain. 2015;138:2347- 2358.

367. Vermeulen RJ, Peeters-Scholte C, Van Vugt J, Barkhof F, Rizzu P, Van Der Schoor SR, Van Der Knaap MS. Fetal origin of brain damage in two infants with a COL4A1 mutation: Fetal and neonatal neuroimaging. Dev Med Child Neurol. 2012;54:197. (Abstract)

368. Vermeulen RJ, Peeters-Scholte C, Van Vugt JJ, Van Vught JJ, Barkhof F, Rizzu P, van der Schoor SR, van der Knaap MS. Fetal origin of brain damage in 2 infants with a COL4A1 mutation: fetal and neonatal MRI. Neuropediatrics. 2011;42:1-3.

369. Viana-Baptista M, Cruz-E-Silva V, Caetano A, Marto JP, Azevedo E., Ferreira C, Pinho-E-Melo T, Silva F, Ros Forteza FJ, Inacio N, et al. Vascular White Matter Lesions in Young Adults: A Neurology Outpatient Clinic Registry. Eur. Neurol. 2019;82:23–31.

370. Viana-Baptista M, De Silva VC, Caetano A, Azevedo E, Ferreira C, De Melo TP, Silva F, Ros J, Inacio NMO, Veiga A, et al. PORTYWHITE-Portuguese registry on incidental white matter lesions of presumed vascular etiology in young adults: Preliminary results. Eur. J. Neurol. 2017;24:89. (Abstract)

371. Vilain C, Van Regemorter N, Verloes A, David P, Van Bogaert P. Neuroimaging fails to identify asymptomatic carriers of familial porencephaly. Am J Med Genet. 2002;112:198- 202.

372. Vitale G, Pichiecchio A, Ormitti F, Tonduti D, Asaro A, Farina L, Piccolo B, Percesepe A, Bastianello S, Orcesi S. Cortical malformations and COL4A1 mutation: Three new cases. Eur J Ped Neurol. 2019;23:410-417.

373. Vodopivec I, Oakley DH, Perugino CA, Venna N, Hedley-Whyte ET, Stone JH. A 44-year-old man with eye, kidney, and brain dysfunction. Ann Neurol. 2016;79:507-519.

374. Wang QH, Zou LP, Zhang MN, Wang YY, Lu Q, Shen YW, He W, Chen HM, Luo XM, Wang J, et al. Phenotypic characterization of COL4A1-related West syndrome. Epilepsy Res. 2020;164:106349.

375. Wang XL, Li CF, Guo HW, Cao BZ. A novel mutation in the HTRA1 gene identified in Chinese CARASIL pedigree. CNS Neuroscience and Therapeutics. 2012;18:867-869.

376. Watanabe J, Okamoto K, Ohashi T, Natsumeda M, Hasegawa H, Oishi M, Miyatake S, Matsumoto N, Fujii Y. Malignant Hyperthermia and Cerebral Venous Sinus Thrombosis After Ventriculoperitoneal Shunt in Infant with Schizencephaly and COL4A1 Mutation. World Neurosurg. 2019;1:446–450.
377. Weng YC, Sonni A, Labelle-Dumais C, de Leau M, Kauffman WB, Jeanne M, Biffi A, Greenberg SM, Rosand J, Gould DB. COL4A1 mutations in patients with sporadic late-onset intracerebral hemorrhage. Ann Neurol. 2012;71:470-477.
378. Wikan TO, Tzoulis C, Hogenesch RI. A mother and her daughter with small vessel disease associated with COL4A1 mutations. Eur. J. Neurol. 2019;26:889.
379. Winkler DT, Lyrer P, Probst A, Devys D, Haufschild T, Haller S, Willi N, Mihatsch MJ, Steck AJ, Tolnay M. Hereditary systemic angiopathy (HSA) with cerebral calcifications, retinopathy, progressive nephropathy, and hepatopathy. J Neurol. 2008;255:77-88.
380. Wu X, Li C, Mao J, Li L, Liu Y, Hou Y. Heterozygous HTRA1 missense mutation in CADASIL-like family disease. Braz J Med Biol Res. 2018;51:e6632.
381. Xia XY, Li N, Cao X, Wu QY, Li TF, Zhang C, Li WW, Cui YX, Li XJ, Xue CY. A novel COL4A1 gene mutation results in autosomal dominant non-syndromic congenital cataract in a Chinese family. BMC Med Genet. 2014;15:97.
382. Xie F, Zhang LS. A Chinese CARASIL Patient Caused by Novel Compound Heterozygous Mutations in HTRA1. J Stroke Cerebrovasc Dis. 2018;27:2840-2842.
383. Yamashita T, Nozaki H, Wakutani Y, Tadokoro K, Nomura E, Takahashi Y, Sato K, Hishikawa N, Takemoto M, Shang J, et al. A Japanese family of autosomal dominant cerebral small vessel disease with heterozygous HTRA1 mutation showing dementia, gait disturbance and subarachnoid hemorrhage. Japenese Soc. Vasc. Cogn. Impair. 2019;5:20–26.
384. Yanagawa S, Ito N, Arima K, Ikeda S. Cerebral autosomal recessive arteriopathy with subcortical infarcts and leukoencephalopathy. Neurology. 2002;58:817-820.
385. Yang M, Li S, Liu J, Qin W, Li G, Shi Y, Zang W, Zhang J. Pedigree study of hereditary small cerebral vascular disease caused by c.821G>A heterozygous mutation of HtrA serine protease-1 gene. Chin. J. Neurol. 2019;52:478–486.
386. Yaramis A, Lochmüller H, Töpf A, Sonmezler E, Yilmaz E, Hiz S, Yis U, Gungor S, Ipek Polat A, Edem P, et al. COL4A1-related autosomal recessive encephalopathy in 2 Turkish children. Neurol. Genet. 2020;6:e392.
387. Yoneda Y, Haginoya K, Arai H, Yamaoka S, Tsurusaki Y, Doi H, Miyake N, Yokochi K, Osaka H, Kato M et al. De novo and inherited mutations in COL4A2, encoding the type IV collagen α2 chain cause porencephaly. Am J Hum Genet. 2012;90:86-90.
388. Yoneda Y, Haginoya K, Kato M, Osaka H, Yokochi K, Arai H, Kakita A, Yamamoto T, Otsuki Y, Shimizu S et al. Phenotypic spectrum of COL4A1 mutations: porencephaly to schizencephaly. Ann Neurol. 2013;73:48-57.
389. Yu Y, Qi X. Uncommon stroke disorders / difficult cases a complicated case of herns. Int J Stroke. 2018;13:28. (Abstract) (a)
390. Yu Y. A Complicated Case of HERNS. Alzheimer's and Dementia. 2018;14:P1295. (Abstract) (b)
391. Yu Z, Cao S, Wu A, Yue H, Zhang C, Wang J, Xia M, Wu J. Genetically Confirmed CARASIL: Case Report with Novel HTRA1 Mutation and Literature Review. World Neurosurg. 2020;143:121–128.

392. Zagaglia S, Selch C, Nisevic JR, Mei D, Michalak Z, Hernandez-Hernandez L, Krithika S, Vezyroglou K, Varadkar SM, Pepler A. Neurologic phenotypes associated with COL4A1/2 mutations: Expanding the spectrum of disease. Neurology. 2018;91:e2078- 2088.
393. Zenteno JC, Crespí J, Buentello-Volante B, Buil JA, Bassaganyas F, Vela-Segarra JI, Diaz-Cascajosa J, Marieges MT. Next generation sequencing uncovers a missense mutation in COL4A1 as the cause of familial retinal arteriolar tortuosity. Graefes Arch Clin Exp Ophthalmol. 2014;252:1789-1794.
394. Zhang WY, Xie F, Lu PL. Two novel heterozygous HTRA1 mutations in two pedigrees with cerebral small vessel disease families. Neurol Sci. 2018;39:497-501.
395. Zhao YY, Duan RN, Ji L, Liu QJ, Yan CZ. Cervical Spinal Involvement in a Chinese Pedigree With Pontine Autosomal Dominant Microangiopathy and Leukoencephalopathy Caused by a 3′ Untranslated Region Mutation of COL4A1 Gene. Stroke. 2019;50:2307–2313.
396. Zhou Q, Chae J, Hershfield M, Sood R, Burgess S, Zavialov A, Chin D, Gadina M, Goldbach-Mansky R, Ombrello A et al. OR13-001 loss-of-function mutations in CECR1, encoding adenosine deaminase 2 (ADA2), cause recurrent fevers and early onset strokes. Pediatr Rheumatol. 2013;11. (Abstract)
397. Zhou Q, Yand D, Ombrello A, Kuehn H, Chae JJ, Zavialov A, Chin D, Stone D, Toro C, Milner J et al. Intermittent fever, immune dysregulation, and systemic vasculopathy due to loss-of-function mutations in adenosine deaminase 2. Arthritis and Rheumatism. 2013;65:S383-384. (Abstract)
398. Zhou Q, Yang D, Ombrello AK, Zavialov AV, Toro C, Stone DL, Chae JJ, Rosenzweig SD, Bishop K, Barron KS et al. Early-onset stroke and vasculopathy associated with mutations in ADA2. N Engl J Med. 2014;370:911-920.
399. Zhuo Z, Cong L, Zhang J, Zhao X. A novel heterozygous HTRA1 mutation is associated with autosomal dominant hereditary cerebral small vessel disease. Mol. Genet. Genomic Med. 2020;8:e1111.
400. Ziaei A, Xu X, Dehghani L, Bonnard C, Reversade B, Shaygannejad V, Pouladi MA. Novel mutation in HTRA1 identified in a family with diffuse demyelination lesions. J Neurochem. 2017;142:94. (Abstract)
401. Ziaei A, Xu X, Dehghani L, Bonnard C, Zellner A, Jin Ng AY, Tohari S, Venkatesh B, Haffner C, Reversade B, et al. Novel mutation in HTRA1 in a family with diffuse white matter lesions and inflammatory features. Neurol. Genet. 2019;5:e345.
402. Zlamy M, Heugenhauser K, Scholl-Buergi S, Zoeggeler T, Sailer-Hoeck M, Brunner J, Karall D. Myalgia and dystrophic gait results in a diagnosis of deaminase 2 deficiency. J. Inherit. Metab. Dis. 2019;42:296–297. (Abstract)
